# CXCL13 suppresses liver regeneration through the negative regulation of HGF signaling

**DOI:** 10.1038/s41419-025-07568-2

**Published:** 2025-05-05

**Authors:** Qun Zhao, Jingyi Wu, Mengyuan Feng, Anjie Zhang, Liwei Fu, Jinglin Chen, Lian Li, Fangzhou Li, Tingting Li, Shu Jin, Shengbao Li, Xianjun Yu

**Affiliations:** 1 Department of Gastroenterology, Taihe Hospital, School of Basic Medical Sciences, Hubei Key Laboratory of Embryonic Stem Cell Research, Hubei University of Medicine, Shiyan, China; 2 Laboratory of Inflammation and Molecular Pharmacology, Biomedical Research Institute, Inflammation-Cancer Transformation and Wudang Chinese Medicine Research, Hubei Talent Introduction and Innovation Demonstration Base, Hubei Provincial Clinical Research Center for Umbilical Cord Blood Hematopoietic Stem Cells, Hubei University of Medicine, Shiyan, China; 3 Department of Clinical Laboratory, Renmin Hospital, Hubei University of Medicine, Shiyan, China

**Keywords:** Cell signalling, Medical research

## Abstract

Insufficient liver regeneration increases the risk of postoperative liver failure following liver transplantation or partial hepatectomy (PHx). Numerous growth factors and cytokines are related to liver regeneration; however, the underlying mechanisms have not been fully elucidated. In this study, CXCL13 was identified as a key factor delaying liver regeneration after PHx. We observed that CXCL13 expression was upregulated in PHx mice and patients following liver resection. CXCL13 deficiency accelerated liver regeneration, whereas these effects were abolished by recombinant murine CXCL13 administration. Moreover, proteomics analyses indicated that HGF levels in the serum after PHx were significantly greater in *Cxcl13*^*−/−*^ mice than in WT mice. Further analysis revealed that CXCL13 deficiency promoted liver regeneration via elevated HGF expression in reparative macrophages and subsequent activated the HGF/c-MET axis in hepatocytes. Additionally, deficiency of macrophage CXCR5, the receptor for CXCL13, augmented liver regeneration and elevated HGF expression after PHx. Mechanistically, CXCL13 inhibited HGF expression in reparative macrophages via CXCR5-mediated AKT/FoxO3a signaling. We further determined that noncanonical NF-κB signaling activation induced CXCL13 expression in hepatic macrophages. Importantly, treatment with CXCL13-neutralizing antibody effectively improved liver regeneration in mice PHx model. Overall, our findings revealed a novel function of CXCL13 in negatively regulating liver regeneration. The underlying mechanism involved CXCL13/CXCR5-mediated FoxO3a signaling, which downregulated HGF expression in reparative macrophages and subsequently attenuated hepatocyte proliferation through inactivating HGF/c-MET signaling. These data suggest that therapeutic targeting of the CXCL13 signaling axis might decrease the risk of postoperative liver failure.

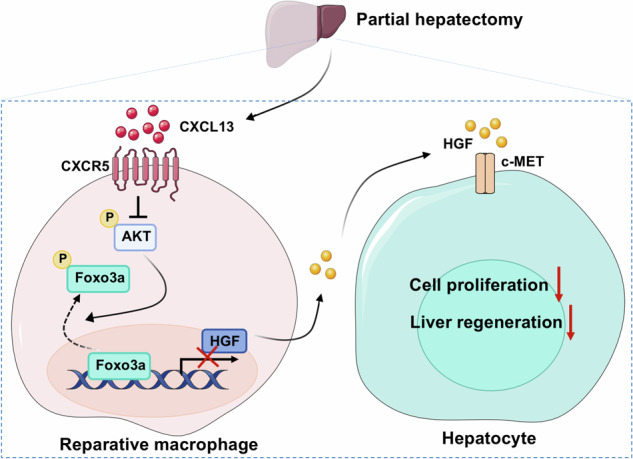

## Introduction

The liver is important for regulating metabolism, immunity, protein synthesis and other physiological functions. After acute injury, partial hepatectomy (PHx) and liver transplantation, the liver has the ability to regenerate and repair itself [1]. Liver failure is caused by insufficient liver regeneration and is associated with increased morbidity and mortality [2]. Accumulating evidence has shown that liver regeneration is closely related to a variety of signaling pathways, including those involving cytokines, growth factors and metabolism [3]. Although many therapeutic strategies improve the ability of the liver to regenerate, the occurrence of liver failure remains high. It is therefore important to delineate the exact mechanisms of liver regeneration and develop treatment strategies for accelerating liver regeneration and improving liver failure.

Immune cells are central players in liver homeostasis and the progression of liver diseases. Abnormalities in immune regulation are reportedly associated with liver injury, liver failure, and other conditions [4–6]. Intrahepatic macrophage populations regulate the expression of secretory factors and play important roles in liver regeneration [7, 8]. The macrophage population consists of both Ly6C^high^ and Ly6C^low^ macrophages. Ly6C^high^ cells are proinflammatory, whereas Ly6C^low^ cells release anti-inflammatory and reparative cytokines [8, 9]. Thus, targeting macrophage dynamics and reparative macrophage polarization might constitute a promising strategy to accelerate liver recovery and regeneration.

Chemokine C-X-C motif ligand 13 (CXCL13), which acts as a chemoattractant by binding to the C-X-C chemokine receptor type 5 (CXCR5), recruits leukocytes into inflammatory sites and is linked with various pathological types [10, 11]. Our previous findings and studies conducted by others have shown that CXCL13 exerted its functions through its cognate receptor CXCR5 [12–14]. CXCL13 is essential for the development of most lymph nodes and is associated with autoimmunity and inflammatory conditions [11, 15]. The aberrant regulation of CXCL13 is related to many inflammatory-mediated diseases, including colitis, sepsis and pulmonary fibrosis [16–18]. Accumulating evidence indicates that CXCL13 is involved in various liver diseases, such as hepatitis B virus-related acute hepatitis and hepatocellular carcinoma [19, 20]. However, the function of CXCL13 in liver regeneration remains largely unclear.

Here, we investigated the function of CXCL13 signaling in liver regeneration and explored its potential mechanism. This study revealed that CXCL13 was significantly upregulated in PHx mice and in patients following liver resection. Functional studies demonstrated that CXCL13 deficiency enhanced liver regeneration by activating HGF/c-MET signaling. Mechanistically, CXCL13 inhibited HGF expression in reparative macrophages via CXCR5-mediated AKT/FoxO3a signaling. We also revealed that the expression of CXCL13 was upregulated in hepatic macrophages by triggering noncanonical NF-κB activity. Notably, neutralization of CXCL13 accelerated liver regeneration. These findings demonstrated that CXCL13 signaling mediates crosstalk between reparative macrophages and hepatocytes to delay liver regeneration through the HGF/c-MET signaling pathway. Thus, our findings provide new insights to better understand the physiological functions of CXCL13 and suggest the blockade of CXCL13 signaling might be an effective strategy to improve liver regeneration.

## Materials and methods

### Samples

Blood samples used in this study were obtained from Renmin Hospital Affiliated to Hubei University of Medicine. Patient’s blood was collected before liver resection and after liver resection. All experiments involving blood samples were approved by the Ethics Committee of Hubei University of Medicine (No. 2023-EER-06).

### Mice

*Cxcl13*^*−/−*^ mice were generated as previously described [12]. Experimental *Cxcl13*^*−/−*^ mice and wild-type (WT) littermate mice were generated by crossing *Cxcl13*^*+/-*^ mice. CXCR5-floxed mice (Cxcr5^F/F^ mice) bearing two loxP sites flanking the entire exon 4 of the CXCR5 gene were generated by GemPharmatech Co. Ltd. (Nanjing, China) using CRISPR/Cas9 gene editing system. CXCR5 knockout (*Cxcr5 cKO*) mice with a deletion of CXCR5 in myeloid cells were generated by crossing *Cxcr5*^*F/F*^ mice with lysozyme M (Lys M)-Cre mice. Cxcr5-overexpressing mice (Cxcr5^oe/+^) were generated by GemPharmatech Co. Ltd. CXCR5-overexpression mice (*Cxcr5 cOE*) were generated by crossing the floxed Cxcr5^oe/+^ mice with Lys M-Cre mice. All the mice in this study were maintained at the Animal Center of Hubei University of Medicine and kept on a 12 h:12 h light/dark cycle with controlled temperature and humidity. Animal experimental were approved by the Institutional Animal Care and Use Committee of Hubei University of Medicine (No. 2023-064).

### Reagents

Fetal Bovine Serum (FBS) was purchased from QmSuero/Tsingmu Biotechnology, Wuhan (Wuhan, China). 100×penicillin-streptomycin solution (JF1100, JSENB) and Dl-Dithiothreitol (JS0070, JSENB) were purchased from HongKong JiSiEnBei International Trade Co., Limited. ELISA kits for CXCL13 (cat.no. CHE0211) and HGF(cat.no. CHE0069) were purchased from 4 A Biotech (Wuhan, China).

### Induction of PHx mouse model

For the 2/3 partial hepatectomy (PHx) model, the left lateral and median hepatic lobes of male mice (8 week of age) were ligated and cut after anesthetizing the mice with inhaled isoflurane. After surgery, the PHx mice were placed on a heating pad until they fully recovered from surgery. Body and liver weights were measured. The mice were then sacrificed, blood and liver samples were collected for further analysis. For the 85% hepatectomy model, the mice were anesthetized, approximately 85% of the liver was cut, and the survival rate of the mice was recorded.

### Induction of CCl_4_-induced liver injury in mice

For CCl_4_-induced liver injury, WT mice and *Cxcl13*^*−/−*^ mice were administrated CCl_4_ (10% in olive oil, 1 mL/kg). Blood and liver tissues were collected at 36 h after CCl_4_ injection for further examination. The survival rate of the mice was recorded.

### Design for the animal experiments

For the anti-HGF neutralizing antibody (HGF Ab)-treated 2/3 PHx mouse model, WT and *Cxcl13*^*−/−*^ mice were injected intraperitoneally with daily HGF Ab or vehicle. For the crizotinib-treated 2/3 PHx mouse model, WT and *Cxcl13*^*−/−*^ mice were injected intraperitoneally with daily crizotinib (2 mg/kg/day) or vehicle. For the recombinant murine CXCL13-treated 2/3 PHx mouse model, *Cxcl13*^*−/−*^ mice were injected daily with recombinant CXCL13 or vehicle. For the NF-κB inhibitor-treated 2/3 PHx mouse model, WT mice were intraperitoneally injected daily with PS1145 (50 mg/kg/day) or vehicle. For therapeutic treatment, WT mice were injected daily with CXCL13 neutralization antibody (anti-CXCL13, 50 μg per mice) or vehicle. At designated time points after PHx, the mice were sacrificed, and samples were collected for further analysis.

### Bone marrow chimeras

Six-week-old recipient mice (WT or *Cxcl13*^*−/−*^) were lethally irradiated with 950 cGy. Bone marrow cells from donor WT mice and *Cxcl13*^*−/−*^ mice were injected via the tail vein of recipient WT mice and *Cxcl13*^*−/−*^ mice to generate chimeric mice: WT → WT, WT→ *Cxcl13*^*−/−*^, *Cxcl13*^*−/−*^→WT and *Cxcl13*^*−/−*^→*Cxcl13*^*−/−*^ mice. Eight weeks after bone marrow transplantation, chimeric mice were subjected to 2/3 PHx.

### ALT/AST measurement

Blood was collected from each mouse and centrifuged at 2000 × *g* at 4 °C for 20 min to separate the serum. The serum ALT and AST levels in the mice were measured using commercial assay kits (231229, Shensuoyoufu, China).

### Isolation and culture of primary mice hepatocytes

Hank’s balanced salt solution (HBSS) was used to perfuse the liver until it turned pale, after which it was perfused with HBSS containing collagenase IV (50 mg/100 mL) for 30 min. The liver was scraped with sterile tweezers and filtered through a 70 mm cell filter. Then, the hepatocytes were resuspended in 45% Percoll (GE Healthcare, Pittsburgh, PA, USA) density gradients, and centrifuged at speed 50 g for 10 min, washed and resuspended. Primary hepatocytes were seeded in collagen-coated plates and then cultured with DMEM containing 5% fetal bovine serum (FBS) for 4 h. The medium was changed to serum-free DMEM, and the cells were grown overnight for further experiments.

### Histological and immunofluorescence

Liver sections were stained with hematoxylin and eosin (H&E) for structural evaluation. For immunofluorescence staining, the sections were dehydrated, and antigens were retrieved and then incubated with the following primary antibodies overnight at 4 °C: anti-CXCL13 (ab112521, Abcam), anti-Ki67 (GB121141, Servicebio, Wuhan, China), anti-HNF4α (ab201460, Abcam) and anti-F4/80 (sc-377009, SantaCruz). Next, the slides were incubated with fluorescence-conjugated secondary antibodies (ZF-0513/ZF-0511, ZSGB-BIO, Beijing, China) after being washed with PBS three times. Finally, the sections were stained with DAPI (MCE, Shanghai, China), and fluorescence images were visualized by fluorescence microscopy (Olympus, BX53 + DP74) or confocal microscopy (Olympus, FV3000RS).

### Quantitative real-time PCR

Total RNA from tissues and cells was extracted using TRIzol (Vazyme, China), and synthesized into cDNA using a HiScript III RT SuperMix Kit (Vazyme, China). Quantitative real-time PCR was carried out using SYBR Green PCR master mix (Takara, Dalian, China) on a CFX96 touch real-time system (Bio-Rad Laboratories).

### Western blotting

Total protein was concentrated, separated by SDS‒PAGE, and then transferred to nitrocellulose membranes. The membranes were blocked with 5% nonfat milk and then probed with the indicated primary antibodies at 4 °C overnight under shaking conditions. The primary antibodies used were: anti-CXCL13 (ab112521, Abcam), anti-GAPDH (GB11002, Servicebio), anti-PCNA (GB11010, Servicebio), anti-Cyclin D1 (2978, Cell Signaling Technology), anti-phospho-MET (Thy1234/1235) (sc-377548, SantaCruz), anti-MET (sc-8057, SantaCruz), anti-phospho-AKT (4060, Cell Signaling Technology), anti-AKT (sc-8312, SantaCruz), anti-phospho-FoxO3a (AP0684, ABclonal, Wuhan, China), rabbit anti-FoxO3a (A9270, ABclonal), HGF(A1193, ABclonal), Tubulin(sc-9168, SantaCruz), LAMIN B1 (sc-6216, SantaCruz), NF-κB2 (4882, Cell Signaling Technology). The next day, the nitrocellulose membranes were washed and then incubated with secondary antibodies (115-035-003, 111-035-003 Jackson ImmunoResearch Laboratories). The membranes were developed with chemiluminescence reagent, and the signals were captured with a ChemiDoc Touch Imaging System (Bio-Rad Laboratories).

### Flow cytometry analysis

Liver immune cells from the livers of PHx mice were purified by centrifugation at 500 × g with 35% Percoll, after which red blood cells were removed. The cells were subsequently incubated with fixable viability dye and then incubated with Fc block and the following fluorophore-conjugated antibodies for 1 h: anti-CD45, anti-CD11b, anti-Ly6G, anti-Ly6C (Biolegend, San Diego, USA). Flow cytometry of cells was carried out by flow cytometry (SONY SA 3800, San Jose, CA, United States).

### RNA sequencing (RNA-seq) analysis

RNA-seq was performed by GeneRead Biotechnology to screen the differentially expressed genes. Briefly, total RNA was isolated from hepatic macrophages of WT and *Cxcl13*^*−/−*^ mice after 2/3 PHx. RNA sequencing was performed on the Illumina HiSeq platform. The raw data can be downloaded from PRJNA1212805.

### Statistical analysis

The data are expressed as the mean ± SEM. SPSS 23.0 software and GraphPad Prism software were used for the statistical analysis by two-tailed Student’s t test, Mann–Whitney’s U test, ANOVA test, Pearson correlation or log-rank. For all statistical tests, P values below 0.05 were deemed to be statistically significant.

## Results

### CXCL13 expression is increased in mice after PHx and in patients following liver resection

**Fig. 1:**
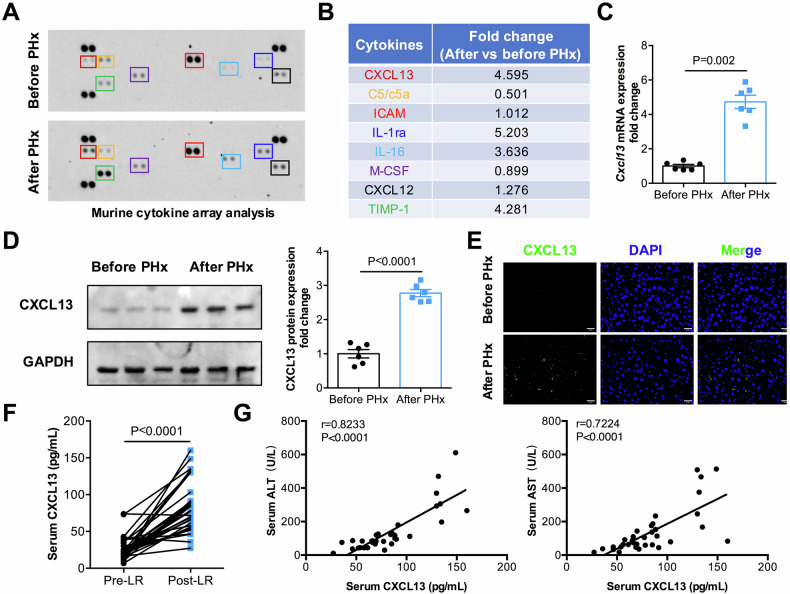
CXCL13 is increased in mice after PHx and in patients with liver resection. **A**, **B** Evaluation of cytokine levels in the serum of mice at 6 h after 2/3 PHx using a cytokine array. Representative images of membrane and the relative signal intensity of indicated cytokines were shown. **C** CXCL13 mRNA expression in liver tissues before and after PHx (n = 6). Statistical significance was made with the Mann–Whitney U test. **D** Western blot analysis of CXCL13 expression in liver tissues before and after 2/3 PHx, and the band intensity was quantified by densitometry (n = 6). Statistical significance was made with the Student’s t test. **E** Representative immunofluorescence images of CXCL13 (green) and DAPI (blue) in mouse livers before and after 2/3 PHx. **F** ELISA analysis of serum CXCL13 levels in patients pre- and post-LR (n = 34). Statistical significance was made with Student’s t test. **G** The correlations between the serum ALT, AST and CXCL13 levels in patients who underwent liver resection were evaluated by Spearman correlation analysis. Correlations were calculated by the Spearman correlation coefficient (n = 34).

To identify potential differential cytokines involved in liver regeneration after PHx, we performed a cytokine array to examine the levels of 40 murine cytokines and chemokines in the serum of mice after 2/3 PHx at 6 h compared with those in sham mice (Fig. 1A). Several cytokines, including the chemokines IL-1, IL-16 and TIMP-1, were shown to be elevated in the serum of the 2/3 PHx model. Interestingly, CXCL13 was increased after 2/3 PHx (Fig. 1B). To address whether increased serum levels of CXCL13 reflect changes in liver tissues, quantitative real-time PCR and western blot analysis of liver tissues revealed that the expression levels of CXCL13 were also significantly elevated after 2/3 PHx (Fig. 1C, D). Immunofluorescence staining of liver tissues revealed increased CXCL13 expression after 2/3 PHx (Fig. 1E). To examine whether CXCL13 is associated with hepatectomy in the clinic, we examined CXCL13 levels in the serum before and after liver resection (LR). Notably, patients post-LR showed significantly higher levels of serum CXCL13 than detected in pre-LR serum (Fig. 1F). Spearman correlation analysis revealed that the serum levels of CXCL13 were positively correlated with ALT and AST levels in patients following LR (Fig. 1G). These findings indicate that CXCL13 levels are significantly increased after partial hepatectomy and suggest a causal role of CXCL13 in liver regeneration.

### CXCL13 deficiency accelerates liver regeneration

**Fig. 2:**
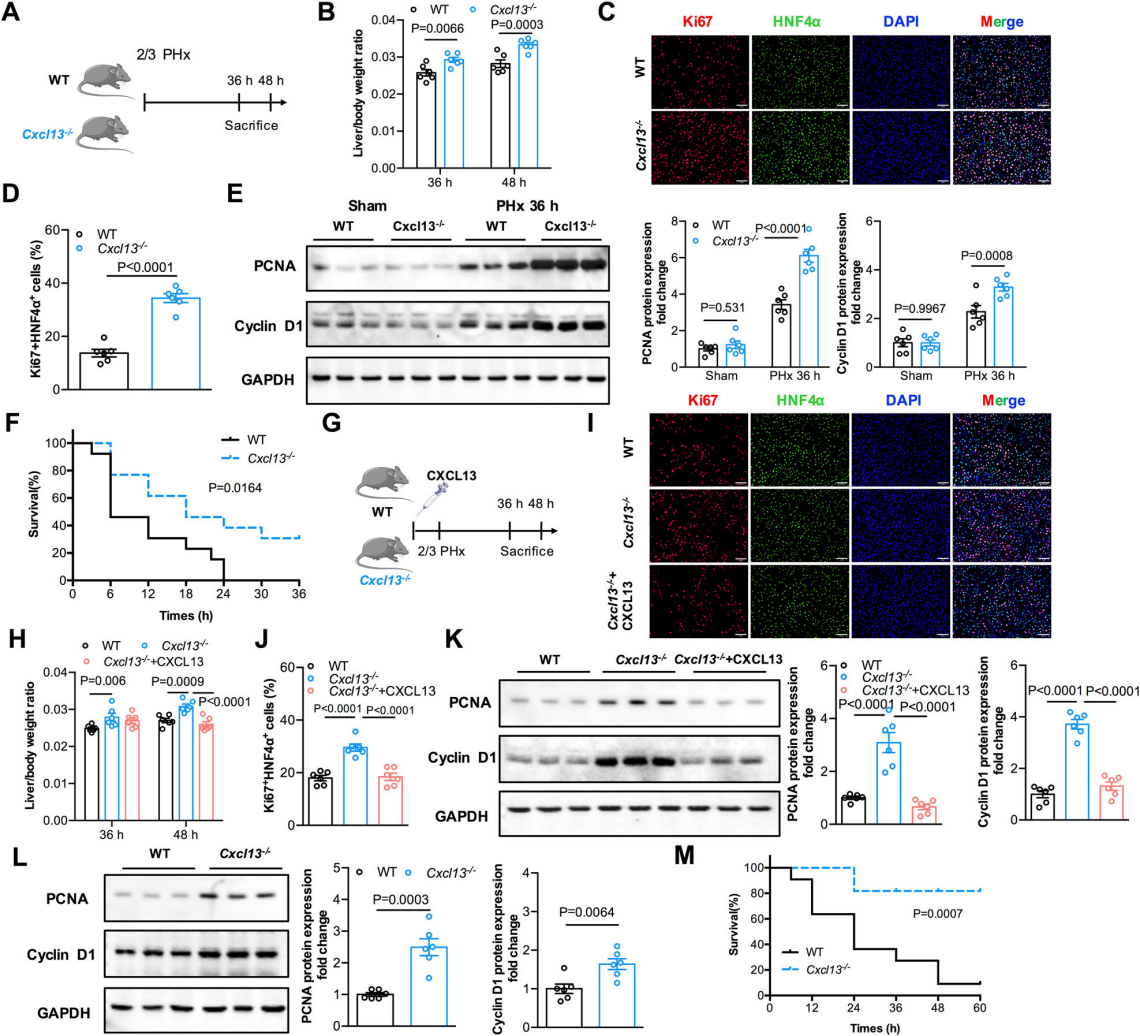
CXCL13 deficiency accelerates liver regeneration. **A** Diagram of the experimental model of WT and *Cxcl13*^*−/−*^ mice with 2/3 PHx. **B** Liver/body weight ratios at the indicated time points after 2/3 PHx (n = 6). Statistical significance was made with the ANOVA test. **C**, **D** Representative immunofluorescence images of Ki67^+^HNF4α^+^ (Ki67: red, HNF4α: green, and DAPI: blue) in the livers of WT and *Cxcl13*^*−/−*^ mice at 36 h, and the percentage of Ki67^+^HNF4α^+^ cells was quantified (n = 6). Statistical significance was made with the Mann–Whitney U test. **E** Western blot analysis of PCNA and Cyclin D1 expression levels in WT and *Cxcl13*^*−/−*^ livers after 2/3 PHx, and the band intensity was quantified by densitometry (n = 6). Statistical significance was made with the ANOVA test. **F** Survival curves of WT and *Cxcl13*^*−/−*^ mice after lethal 85% PHx (WT, n = 13; *Cxcl13*^*−/−*^, n = 13). Statistical significance was made with the Log rank test. **G** Diagram of the experimental model of recombinant CXCL13 treatment in WT and *Cxcl13*^*−/−*^ mice. **H** Liver/body weight ratios of the indicated mice after 2/3 PHx (n = 6). Statistical significance was made with the ANOVA test. **I, J** Representative immunofluorescence costaining images of Ki67^+^HNF4α^+^ liver cells in the indicated mice at 36 h and quantification of the percentage of Ki67^+^HNF4α^+^ cells (n = 6). Statistical significance was made with the ANOVA test. **K** Western blot analysis of PCNA and Cyclin D1 expression in liver tissues after 2/3 PHx, and the band intensity was quantified by densitometry (n = 6). Statistical significance was made with the ANOVA test. **L** Western blot analysis of PCNA and Cyclin D1 expression in WT and *Cxcl13*^*−/−*^ livers after CCl_4_ injection, and the band intensity was quantified by densitometry (n = 6). Statistical significance was made with the Student’s t test. **M** Survival curves of WT and *Cxcl13*^*−/−*^ mice (WT, n = 11; *Cxcl13*^*−/−*^, n = 11) after CCl_4_ injection. Statistical significance was made with the Log rank test.

*Cxcl13*^*−/−*^ mice and littermate mice were used to investigate the role of CXCL13 in liver regeneration. The genotypes of the mice were detected by PCR (Supplementary Fig. 1A). Under normal conditions, no significant changes in body weight, liver weight, or the liver/body weight ratio were observed between *Cxcl13*^*−/−*^ and WT mice (Supplementary Fig. 1B). Next, *Cxcl13*^*−/−*^ and WT mice were subjected to 2/3 PHx to investigate the effects of CXCL13 on liver regeneration (Fig. 2A). Notably, a significant increase in the liver/body weight ratio was detected in the *Cxcl13*^*−/−*^ mice compared with the WT mice (Fig. 2B). Moreover, the serum levels of ALT and AST were lower in *Cxcl13*^*−/−*^ mice than in WT mice after 2/3 PHx (Supplementary Fig. 1C). To explore the effect of CXCL13 deficiency on hepatocyte proliferation, hepatocyte nuclear factor 4α (HNF4α) and Ki67 double staining was performed for immunofluorescence analysis. In line with rapid liver restoration, *Cxcl13*^*−/−*^ mice presented an increased number of Ki67^+^HNF4α^+^ cells after 2/3 PHx (Fig. 2C, D). Consistently, the protein expression levels of PCNA and Cyclin D1 were higher in *Cxcl13*^*−/−*^ livers than in WT livers (Fig. 2E). In agreement with these results, Cyclin A2, Cyclin B1, Cyclin D1 and Cyclin E1 expression levels were significantly elevated in *Cxcl13*^*−/−*^ mice (Supplementary Fig. 1D). Because greater than 80% hepatectomy increased mortality due to damaged regeneration of small-for-size liver remnants [21], we performed 85% PHx and found that, compared with WT mice, *Cxcl13*^*−/−*^ mice presented an increased survival rate (Fig. 2F). Together, these data indicate that CXCL13 delays liver regeneration after PHx.

To explore the effect of reintroduction of CXCL13 in the PHx model, we reintroduced CXCL13 back into the livers of *Cxcl13*^*−/−*^ mice by injecting recombinant mouse CXCL13 (Fig. 2G). CXCL13 treatment reduced the liver/body weight ratios and increased ALT and AST levels in *Cxcl13*^*−/−*^ mice after 2/3 PHx (Fig. 2H and Supplementary Fig. 1E). Moreover, CXCL13 treatment decreased the number of Ki67^+^HNF4α^+^ cells in *Cxcl13*^*−/−*^ mice (Fig. 2I, J). Consistently, the protein expression levels of PCNA and Cyclin D1 were inhibited in *Cxcl13*^*−/−*^ mice after CXCL13 treatment following 2/3 PHx (Fig. 2K). These results indicate that the re-expression of CXCL13 dampens the increase in liver regeneration in *Cxcl13*^*−/−*^ mice after PHx.

To further confirm the inhibitory role of CXCL13 in liver regeneration, another murine model of liver regeneration involving CCl_4_ exposure was used (Supplementary Fig. 1F). CXCL13 deficiency decreased liver necrosis and reduced the levels of ALT and AST in the serum after CCl_4_ exposure (Supplementary Fig. 1G, H). Notably, the expression levels of PCNA and Cyclin D1 were significantly increased in the livers of *Cxcl13*^*−/−*^ mice (Fig. 2L). Furthermore, we found that *Cxcl13*^*−/−*^ mice presented significantly lower mortality rates than did WT mice following CCl_4_ exposure (Fig. 2M). Together, our findings suggest that CXCL13 delays liver regeneration after PHx or CCl_4_-induced injury.

### CXCL13 deficiency enhances liver regeneration by promoting the HGF/c-MET pathway after PHx

To determine whether CXCL13 directly facilitates hepatocellular proliferation, WT and *Cxcl13*^*−/−*^ primary hepatocytes were stimulated with or without EGF/HGF. There were no significant differences in PCNA or Cyclin D1 expression between WT and *Cxcl13*^*−/−*^ hepatocytes (Supplementary Fig. 2A, B). Moreover, WT and *Cxcl13*^*−/−*^ hepatocytes were stimulated with EGF/HGF and recombinant mouse CXCL13. Notably, there was no difference in the expression levels of Cyclin D1 and PCNA between WT and *Cxcl13*^*−/−*^ hepatocytes treated with EGF/HGF or recombinant CXCL13 (Supplementary Fig. 2C, D). Taken together, these data indicate that CXCL13 does not directly influence hepatocyte proliferation, suggesting that CXCL13 deficiency likely facilitates hepatocellular proliferation during liver regeneration through other mechanism.

**Fig. 3:**
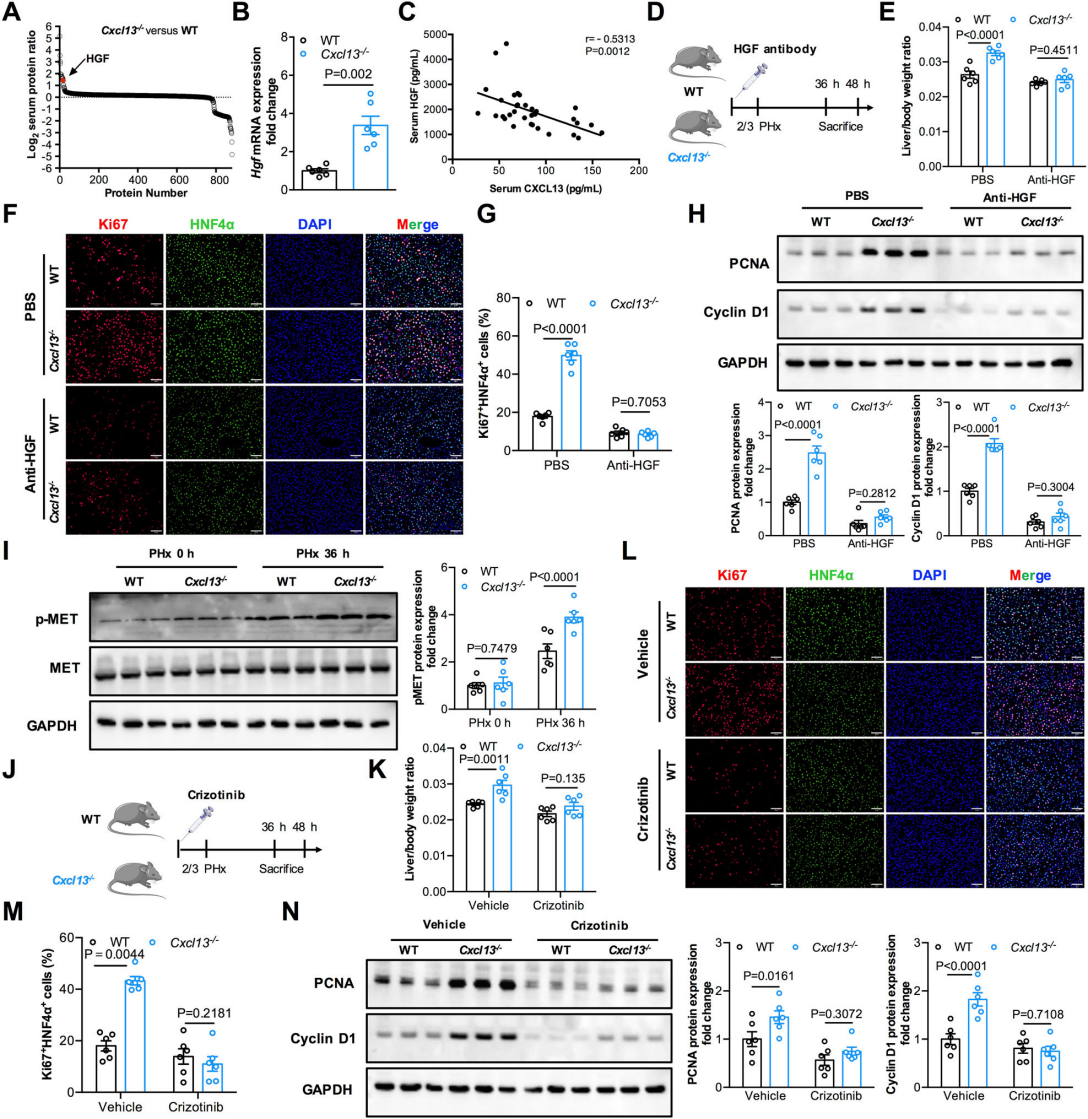
CXCL13 deficiency enhances liver recovery by promoting the HGF/c-MET pathway after PHx. **A** Ratio plot of the changes in protein abundance in the serum proteomes of WT and *Cxcl13*^*−/−*^ mice at 36 h after 2/3 PHx (n = 3). **B** Relative levels of HGF mRNA transcripts in WT and *Cxcl13*^*−/−-*^ livers at 36 h after 2/3 PHx (n = 6). Statistical significance was made with the Mann–Whitney U test. **C** The correlations between the serum CXCL13 and HGF levels in patients post-LR were evaluated by Spearman correlation analysis. Correlations were calculated by the Spearman correlation coefficient (n = 34). **D** Diagram of the experimental model of WT and *Cxcl13*^*−/−*^ mice treated with or without HGF antibodies in PHx mice. **E** Liver/body weight ratios of WT and *Cxcl13*^*−/−*^ mice treated with or without HGF antibodies (n = 6). Statistical significance was made with the ANOVA test. **F**, **G** Representative immunofluorescence images of liver Ki67^+^HNF4α^+^ cells in the indicated mice at 36 h and quantification of the percentage of Ki67^+^HNF4α^+^ cells (n = 6). Statistical significance was made with the ANOVA test. **H** Western blot analysis of PCNA and Cyclin D1 expression levels in liver tissues from the indicated mice at 2/3 PHx, and the band intensity was quantified by densitometry (n = 6). Statistical significance was made with the ANOVA test. **I** Western blot analysis of the phosphorylation of c-MET (p-MET) and total c-MET in the livers of the indicated mice, and the pMET/MET band intensity was quantified by densitometry (n = 6). Statistical significance was made with the ANOVA test. **J** Diagram of the experimental model of WT and *Cxcl13*^*−/−*^ mice treated with or without the c-MET inhibitor crizotinib in the 2/3 PHx model. **K** Liver/body weight ratios of the indicated mice after 2/3 PHx (n = 6). Statistical significance was made with the ANOVA test. **L**, **M** Representative immunofluorescence images of Ki67^+^HNF4α^+^ liver cells in the indicated mice at 36 h and quantification of the percentage of Ki67^+^HNF4α^+^ cells (n = 6). Statistical significance was made with the ANOVA test. **N** Western blot analysis of PCNA and Cyclin D1 expression in liver tissues, and the band intensity was quantified by densitometry (n = 6). Statistical significance was made with the ANOVA test.

To investigate which and how CXCL13 delays liver regeneration, we performed proteomics analyses of serum samples from *Cxcl13*^*−/−*^ and WT mice after 2/3 PHx (Supplementary Fig. 2E). HGF, a secreted protein involved in the pathogenesis of hepatocyte proliferation and liver regeneration, was significantly increased in the serum of *Cxcl13*^*−/−*^ mice compared with that in the serum of WT mice after 2/3 PHx (Fig. 3A and Supplementary Fig. 2F**)**. Consistently, the levels of HGF were also greater in the livers of *Cxcl13*^*−/−*^ mice than in those of WT mice after 2/3 PHx (Fig. 3B). By using Spearman correlation analysis, we observed a significant negative correlation between the serum CXCL13 and HGF levels in patients after liver resection (Fig. 3C**)**, but no correlation has been found before liver resection (Supplementary Fig. 2G**)**. We thus speculated that HGF is involved in CXCL13 deficiency-promoted liver regeneration. To further verify that CXCL13 deficiency facilitates liver regeneration by increasing HGF expression, an anti-HGF neutralizing antibody (HGF Ab) was administered to WT and *Cxcl13*^*−/−*^ mice after 2/3 PHx (Fig. 3D). HGF Ab treatment abolished the augmented liver/body weight ratios in *Cxcl13*^*−/−*^ mice (Fig. 3E). HGF Ab treatment increased the ALT and AST levels in *Cxcl13*^*−/−*^ mice (Supplementary Fig. 2H). Similarly, HGF Ab treatment also decreased the number of Ki67^+^HNF4α^+^ cells in *Cxcl13*^*−/−*^ mice after 2/3 PHx (Fig. 3F, G). In addition, HGF Ab treatment eliminated the differences in the protein expression levels of PCNA and Cyclin D1 in *Cxcl13*^*−/−*^ mice compared with those in WT mice (Fig. 3H). The above results further confirmed that CXCL13 deficiency accelerates liver regeneration by upregulating HGF expression.

Substantial activation of HGF/c-MET signaling is associated with hepatocyte proliferation and liver regeneration, and inhibition of these signaling pathways results in liver failure [22–24]. Compared with those of WT littermates, the levels of phosphorylated c-MET (pMET) were significantly increased in the livers of *Cxcl13*^*−/−*^ mice after 2/3 PHx (Fig. 3I). To further determine whether c-MET activation is critical for CXCL13 deficiency-induced liver regeneration, WT and *Cxcl13*^*−/−*^ mice were pretreated with crizotinib, a clinically available c-MET inhibitor, followed by 2/3 PHx (Fig. 3J). Crizotinib treatment attenuated the increase in the liver/body weight ratios in *Cxcl13*^*−/−*^ mice (Fig. 3K). Crizotinib treatment eliminated the differences in the serum levels of ALT and AST in *Cxcl13*^*−/−*^ and WT mice (Supplementary Fig. 2I). Similarly, crizotinib treatment eliminated the increase in the number of Ki67^+^HNF4α^+^ cells and increased PCNA and Cyclin D1 expression levels in the livers of *Cxcl13*^*−/−*^ and WT mice (Fig. 3L–N). Taken together, these data demonstrate that CXCL13 deficiency enhances liver regeneration by promoting the activation of HGF/c-MET signaling pathway.

### CXCL13 deficiency upregulates HGF expression in reparative macrophages during liver regeneration

Given the increased proliferation rate of hepatocytes in *Cxcl13*^*−/−*^ mice after PHx, HGF expression was measured in WT and *Cxcl13*^*−/−*^ mouse primary hepatocytes. No significant changes in HGF expression were detected between WT and *Cxcl13*^*−/−*^ mice (Supplementary Fig. 3A). Additionally, primary hepatocytes were treated with recombinant CXCL13, and no overt difference in HGF expression was detected between the control and recombinant CXCL13 treatment groups **(**Supplementary Fig. 3B**)**. Moreover, the levels of HGF expression in hepatocytes from WT and *Cxcl13*^*−/−*^ mice after 2/3 PHx were not significantly different, implying that CXCL13 did not alter HGF expression in hepatocytes (Supplementary Fig. 3C). Hence, these data suggest that CXCL13 downregulates HGF expression through other mechanisms during liver regeneration.

**Fig. 4:**
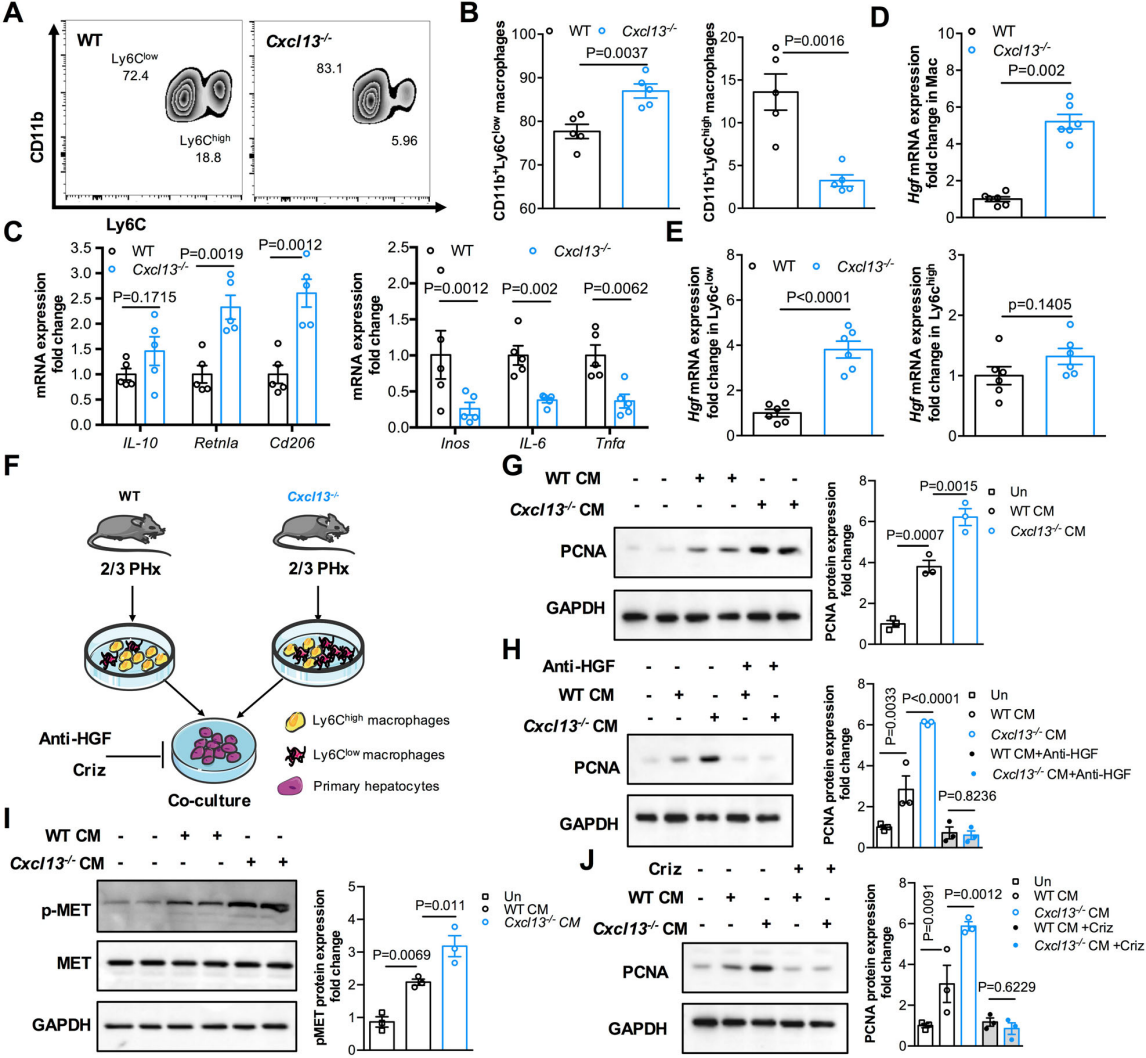
CXCL13 deficiency upregulates HGF expression in reparative macrophages during liver regeneration. **A**, **B** Representative flow cytometric plots and quantification of Ly6C^low^ and Ly6C^high^ hepatic macrophages from WT and *Cxcl13*^*−/−*^ mice at 36 h after 2/3 PHx (n = 5). Statistical significance was made with the Student’s t test. **C** Relative levels of Ly6C^low^ and Ly6C^high^ macrophage marker mRNA transcripts in hepatic macrophages from WT and *Cxcl13*^*−/−*^ livers (n = 5). Statistical significance was made with the Student’s t test. **D** Relative levels of HGF mRNA transcripts in hepatic macrophages from WT and *Cxcl13*^*−/−*^ livers at 36 h after 2/3 PHx (n = 6). Statistical significance was made with the Mann–Whitney U test. **E** Relative levels of HGF mRNA transcripts in Ly6C^low^ and Ly6C^high^ hepatic macrophages from WT and *Cxcl13*^*−/−*^ livers at 36 h after 2/3 PHx (n = 6). Statistical significance was made with the Student’s t test. **F** Diagram of the experiments involving the coculture of primary hepatocytes and hepatic macrophages. **G** The levels of PCNA protein expression in cocultured primary hepatocytes and hepatic macrophages from WT and *Cxcl13*^*−/−*^ mice after 2/3 PHx, and the band intensity was quantified by densitometry (n = 3). Statistical significance was made with the ANOVA test. **H** PCNA expression in cocultured cells as indicated, and the band intensity was quantified by densitometry (n = 3). Statistical significance was made with the ANOVA test. **I** Western blot analysis of p-Met expression in cocultured primary hepatocytes and hepatic macrophages from WT and *Cxcl13*^*−/−*^ mice after 2/3 PHx, and the pMET/MET band intensity was quantified by densitometry (n = 3). Statistical significance was made with the ANOVA test. **J** PCNA expression in cocultured cells as indicated, and the band intensity was quantified by densitometry (n = 3). Statistical significance was made with the ANOVA test.

Because CXCL13 can recruit inflammatory cells, we examined whether CXCL13 deficiency affects immune cell infiltration during liver regeneration after 2/3 PHx. Flow cytometry assays revealed that the infiltration of neutrophil populations was similar in the livers of WT and *Cxcl13*^*−/−*^ mice after 2/3 PHx (Supplementary Fig. 3D). Macrophage polarization plays a critical role in liver regeneration after acute injury [25, 26]. Compared with those in WT mice, there were more anti-inflammatory Ly6C^low^ macrophages and fewer proinflammatory Ly6C^high^ macrophages in *Cxcl13*^*−/−*^ livers after 2/3 PHx **(**Fig. 4A, B**)**. Consistently, the expression levels of Ly6C^low^ macrophage markers (*Il-10, Retnla, Cd206*) were increased, whereas the expression levels of Ly6C^high^ macrophage markers (*Inos, Il-6, Tnf-α*) were decreased in the hepatic macrophages of *Cxcl13*^*−/−*^ mice after 2/3 PHx **(**Fig. 4C**)**. Interestingly, hepatic macrophages from *Cxcl13*^*−/−*^ mice after 2/3 PHx presented higher HGF expression than did those from WT mice **(**Fig. 4D**)**. Furthermore, increased HGF expression was observed mainly in Ly6C^low^ hepatic macrophages from *Cxcl13*^*−/−*^ livers after 2/3 PHx (Fig. 4E**)**. Therefore, we suspect that reparative macrophages are responsible for the upregulation of HGF expression in *Cxcl13*^*−/−*^ mice after PHx.

To further investigate the effect of *Cxcl13*^*−/−*^ hepatic macrophages on the proliferation of hepatocytes in vitro, primary mouse hepatocytes were cocultured with hepatic macrophages from WT and *Cxcl13*^*−/−*^ livers after 2/3 PHx (Fig. 4F). Compared with that in WT hepatocytes, the expression of PCNA in hepatocytes was greater under the *Cxcl13*^*−/−*^ hepatic macrophage coculture conditions (Fig. 4G). To further validate that HGF signaling is necessary for CXCL13 deficiency-promoted hepatocyte proliferation, HGF Ab was added to the cocultured conditions. As expected, HGF Ab treatment abolished the increased levels of PCNA in *Cxcl13*^*−/−*^ hepatic macrophage coculture conditions (Fig. 4H**)**. Consistent with the in vivo findings, the levels of pMET were increased in the *Cxcl13*^*−/−*^ hepatic macrophage coculture conditions compared with those in the WT hepatic macrophage coculture conditions (Fig. 4I). The c-MET inhibitor crizotinib also decreased the PCNA protein levels in the *Cxcl13*^*−/−*^ and WT hepatic macrophage coculture conditions (Fig. 4J). These data suggest that CXCL13 deficiency facilitates the production of HGF from reparative macrophages to promote hepatocyte proliferation after PHx.

### CXCR5 deficiency in macrophages promotes liver regeneration after PHx

**Fig. 5:**
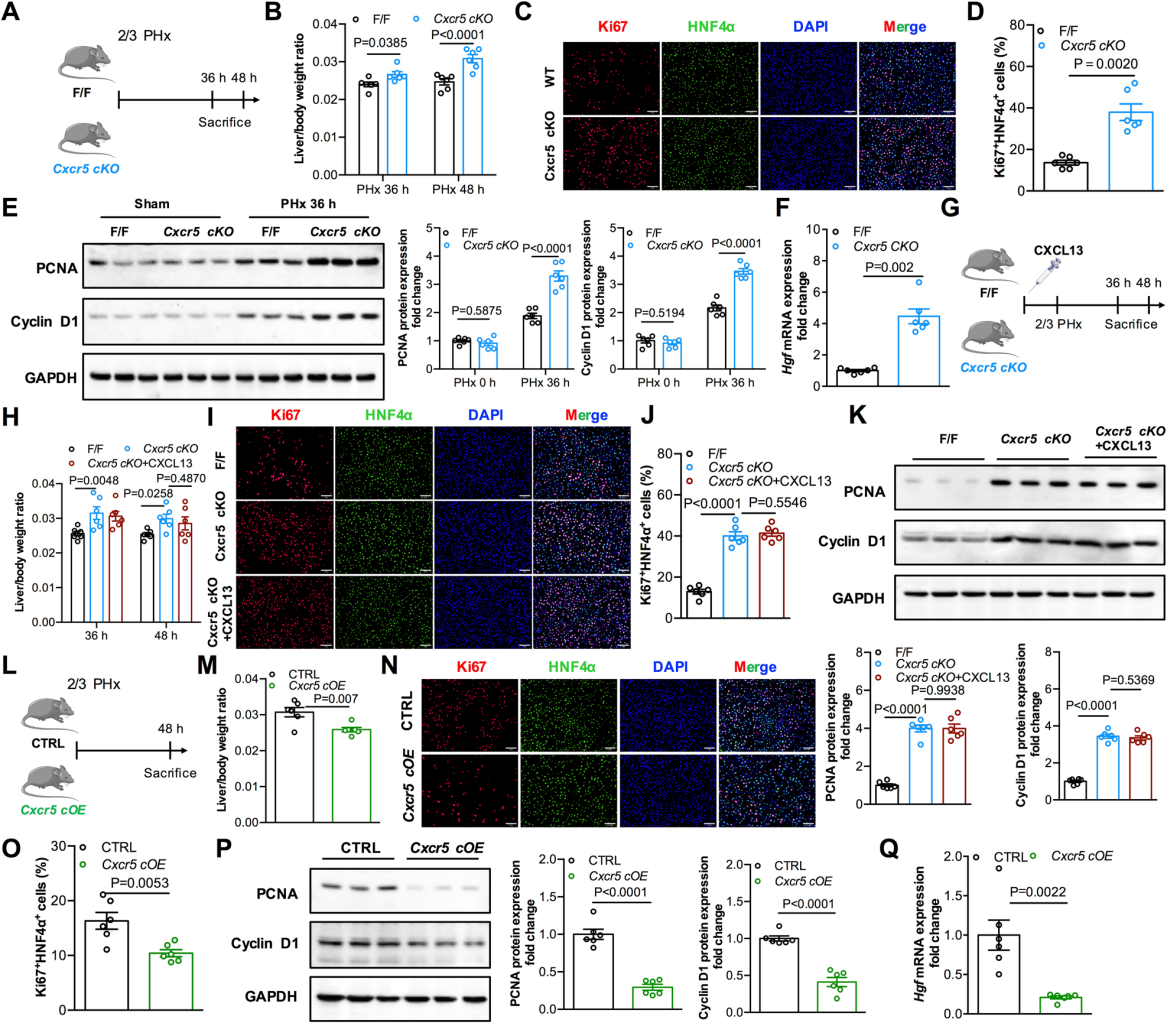
Macrophage CXCR5 suppresses liver regeneration after PHx. **A** Diagram of the experimental model of myeloid-specific LysM-Cre Cxcr5 knockout (*Cxcr5 cKO*) mice and littermate control (*Cxcr5*^*F/F*^, F/F) in PHx mice model. **B** Liver/body weight ratios of *Cxcr5 cKO* and F/F mice at 36 h and 48 h after 2/3 PHx (n = 6). Statistical significance was made with the ANOVA test. **C**, **D** Representative immunofluorescence images of Ki67^+^HNF4α^+^ liver cells in the indicated mice at 36 h after 2/3 PHx and quantification of the percentage of Ki67^+^HNF4α^+^ cells (n = 6). Statistical significance was made with the Mann–Whitney U test. **E** Western blot analysis of PCNA and Cyclin D1 expression in *Cxcr5 cKO* and F/F livers after 2/3 PHx, and the band intensity was quantified by densitometry (n = 6). Statistical significance was made with the ANOVA test. **F** Relative levels of HGF mRNA transcripts in F/F and *Cxcr5 cKO* livers at 36 h after 2/3 PHx (n = 6). Statistical significance was made with the Mann–Whitney U test. **G** Diagram of the experimental model of recombinant CXCL13 treatment in *Cxcr5 CKO* *mice*. **H** Liver/body weight ratios of *Cxcr5 cKO* mice treated with and without recombinant CXCL13 after 2/3 PHx (n = 6). Statistical significance was made with the ANOVA test. **I**, **J** Representative immunofluorescence images of Ki67^+^HNF4α^+^ liver cells in the indicated mice at 36 h and quantification of Ki67^+^HNF4α^+^ cells (n = 6). Statistical significance was made with the ANOVA test. **K** Western blot analysis of PCNA and Cyclin D1 expression levels in liver tissues after 2/3 PHx, and the band intensity was quantified by densitometry (n = 6). Statistical significance was made with the ANOVA test. **L** Diagram of the experimental model of CTRL and *Cxcr5 cOE* mice at 2/3 PHx. **M** Liver/body weight ratios in CTRL and *Cxcr5 cOE* mice (n = 6). Statistical significance was made with the Student’s t test. **N**, **O** Representative immunofluorescence images of liver Ki67^+^HNF4α^+^ cells in CTRL and *Cxcr5 cOE* mice at 48 h and quantification of the percentage of Ki67^+^HNF4α^+^ cells (n = 6). Statistical significance was made with the Student’s t test. **P** Western blot analysis of PCNA and Cyclin D1 expression levels in CTRL and *Cxcr5 cOE* livers after 2/3 PHx, and the band intensity was quantified by densitometry (n = 6). Statistical significance was made with the Student’s t test. **Q** Relative levels of HGF mRNA transcripts in CTRL and *Cxcr5 cOE* livers after 2/3 PHx (n = 6). Statistical significance was made with the Mann–Whitney U test.

CXCL13 is the ligand for the chemokine receptor CXCR5 and mediates inflammation and disease pathogenesis [11]. Considering the significant role of CXCL13 in macrophages during liver regeneration, we were interested in investigating the function of CXCR5 in macrophages with respect to liver regeneration. We crossbred *Cxcr5*^*F/F*^ mice with LysM-Cre mice in which CXCR5 in the myeloid lineage, including macrophages, was specifically deleted; these mice are referred to as *Cxcr5 cKO* mice (Supplementary Fig. 4A**)**. The genotypes of the mice were confirmed by PCR (Supplementary Fig. 4B**)**. There were no significant differences in body weights, liver weights, or liver/body weight ratios between *Cxcr5 cKO* and Cxcr5^F/F^ mice under normal healthy conditions (Supplementary Fig. 4C**)**. Next, *Cxcr5 cKO* mice and Cxcr5^F/F^ mice were subjected to 2/3 PHx to assess the role of CXCR5 in liver regeneration (Fig. 5A). The results indicated that a significantly improved liver/body weight ratios in *Cxcr5 cKO* mice (Fig. 5B). Moreover, *Cxcr5 cKO* mice presented lower ALT and AST levels after 2/3 PHx (Supplementary Fig. 4D). In agreement with rapid liver regeneration, the number of Ki67^+^HNF4α^+^ cells were greater in *Cxcr5 cKO* livers than in Cxcr5^F/F^ livers after 2/3 PHx (Fig. 5C, D). Consistent with these findings, *Cxcr5 cKO* mice presented increased expression levels of PCNA and Cyclin D1 proteins in the liver compared with Cxcr5^F/F^ mice (Fig. 5E). Remarkably, HGF expression was upregulated in *Cxcr5 cKO* hepatic macrophages compared with that in Cxcr5^F/F^ mice after 2/3 PHx (Fig. 5F). Previous studies have shown that LysM-Cre is expressed in both macrophages and neutrophils [27]. To assess whether the effects found in *Cxcr5 cKO* mice are dependent on neutrophils, we next used an anti-Ly6G antibody to delete neutrophils in the PHx mouse model (Supplementary Fig. 4E). In the absence of neutrophils, the acceleration of liver regeneration in *Cxcr5 cKO* mice remained greater than that in Cxcr5^F/F^ mice after 2/3 PHx, as evidenced by an increase in the liver weight/body weight ratios and a greater percentage of Ki67^+^HNF4α^+^ cells (Supplementary Fig. 4F-H). Collectively, these results indicate that CXCR5 deficiency in macrophages contributes to improve liver regeneration after PHx.

To further explore whether CXCL13 delays liver regeneration through CXCR5, we treated *Cxcr5 cKO* mice with recombinant mouse CXCL13 after 2/3 PHx (Fig. 5G). Notably, treatment of *Cxcr5 cKO* mice with recombinant CXCL13 did not delay liver regeneration, as demonstrated by the lack of significant differences in liver weight/body weight ratios (Fig. 5H), levels of ALT and AST (Supplementary Fig. 4I), percentages of Ki67^+^HNF4α^+^ cells (Fig. 5I, J), and levels of PCNA and Cyclin D1 (Fig. 5K), compared with *Cxcr5 cKO* mice. Taken together, these results demonstrated that the role of CXCL13 in the inhibition of liver regeneration is mediated by CXCR5 receptor.

We next explored whether elevated CXCR5 expression in macrophages could delay liver regeneration. We generated conditional CXCR5-overexpressing mice, and then crossed them with LysM-Cre mice to obtain CXCR5 conditional overexpression mice, referred to as *Cxcr5 cOE* mice (Supplementary Fig. 4J). The genotypes of the mice were confirmed by PCR (Supplementary Fig. 4K). There were no obvious differences in body weights, liver weights, or liver/body weight ratios between the *Cxcr5 cOE* mice and their control mice (Supplementary Fig. 4L). Compared with control mice, *Cxcr5 cOE* mice presented lower liver/body weight ratios at 48 h after 2/3 PHx (Fig. 5L, M). The number of Ki67^+^HNF4α^+^ cells was lower in the livers of *Cxcr5 cOE* mice than in those of control mice after 2/3 PHx (Fig. 5N, O). Similarly, the protein expression levels of PCNA and Cyclin D1 were decreased in *Cxcr5 cOE* mice (Fig. 5P). Notably, the expression of HGF in *Cxcr5 cOE* hepatic macrophages was much lower than that in control littermates after PHx (Fig. 5Q). Taken together, these results suggest that CXCL13/CXCR5 signaling in hepatic macrophages inhibits HGF production, leading to delayed liver regeneration after PHx.

### CXCL13 signaling downregulates HGF expression via modulation of the AKT/FoxO3a axis in reparative macrophages

**Fig. 6:**
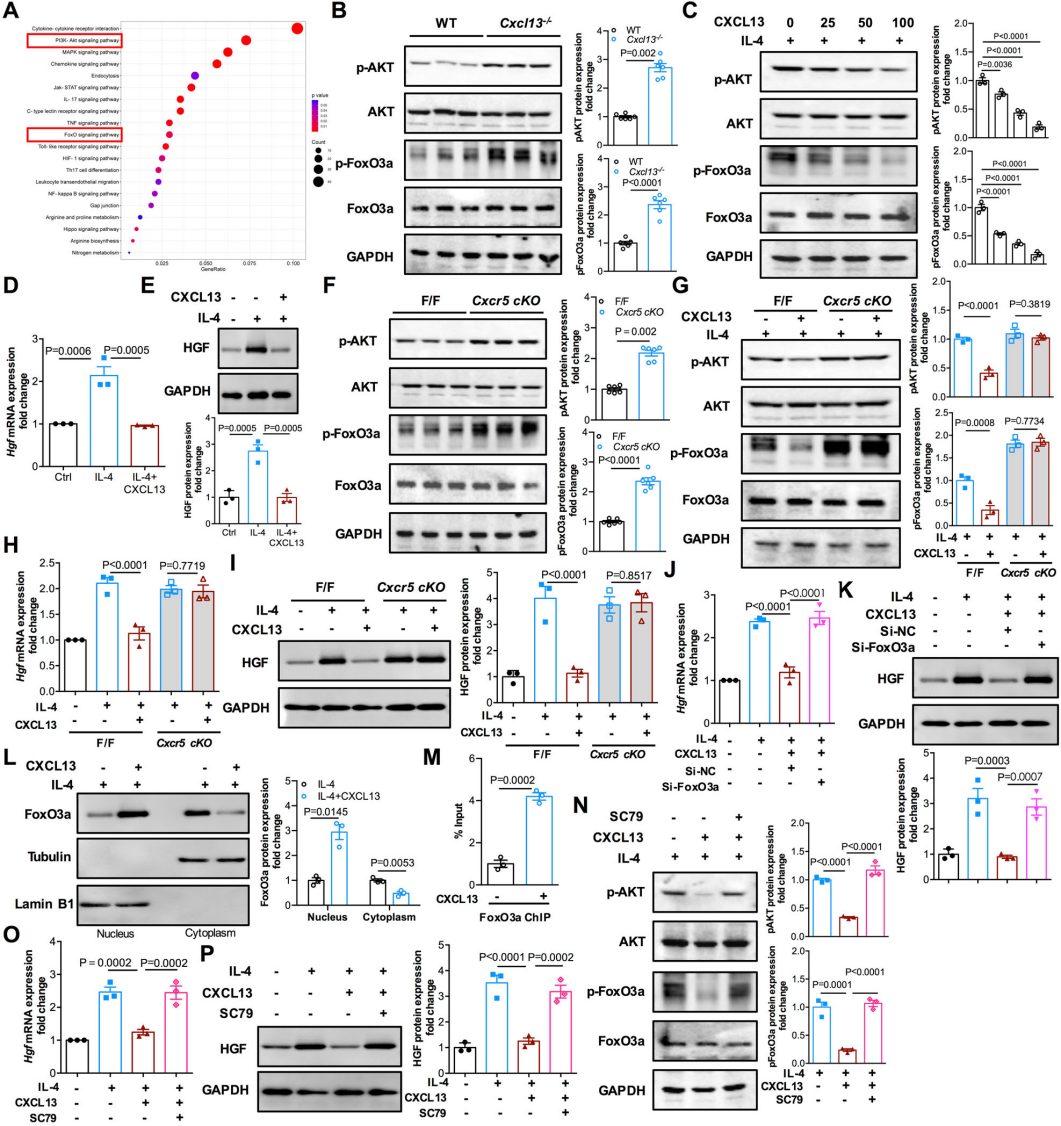
CXCL13 downregulates HGF expression via AKT/FoxO3a signaling in reparative macrophages. **A** Hepatic macrophages from *Cxcl13*^*−/−*^ and WT mice at 36 h after 2/3 PHx were subjected to RNA-seq (n = 3). Kyoto Encyclopedia of Genes and Genomes enrichment analyses of enriched signaling pathways. **B** Western blot analysis of the phosphorylation and total expression of AKT and FoxO3a in hepatic macrophages from *Cxcl13*^*−/−*^ and WT livers at 36 h after 2/3 PHx, and the pAKT/AKT and pFoxO3a/FoxO3a band intensities were quantified by densitometry (n = 6). Statistical significance was made with the Mann–Whitney U test in pAKT and the Student’s t test in pFoxO3a. **C** BMDMs were pretreated with IL-4 for 2 h and then stimulated with recombinant CXCL13 (50 ng/mL) at the indicated concentrations for 1 h. The levels of AKT and FoxO3a phosphorylation were detected, and the pAKT/AKT and pFoxO3a/FoxO3a band intensities were quantified by densitometry (n = 3). Statistical significance was made the ANOVA test. **D** BMDMs were pretreated with IL-4 for 2 h and then stimulated with recombinant CXCL13 for 24 h. The relative levels of HGF mRNA transcripts were measured (n = 3). Statistical significance was made with the ANOVA test. **E** The relative levels of HGF protein expression were measured, and the HGF band intensities were quantified by densitometry (n = 3). Statistical significance was made with the ANOVA test. **F** Western blot analysis of the phosphorylation and total expression of AKT and FoxO3a in hepatic macrophages from *Cxcr5 cKO* and *Cxcr5*^*F/F*^ livers at 36 h after 2/3 PHx, and the pAKT/AKT and pFoxO3a/FoxO3a band intensities were quantified by densitometry (n = 6). Statistical significance was made with the Mann–Whitney U test in pAKT and the Student’s t test in pFoxO3a. **G** BMDMs from *Cxcr5*^*F/F*^ and Cxcr5 cKO mice were pretreated with IL-4 for 2 h and then stimulated with recombinant CXCL13 for 1 h. The levels of AKT and FoxO3a phosphorylation were detected, and the pAKT/AKT and pFoxO3a/FoxO3a band intensities were quantified by densitometry (n = 3). Statistical significance was made the ANOVA test. **H** BMDMs from *Cxcr5*^*F/F*^ and *Cxcr5 cKO* mice were pretreated with IL-4 for 2 h and then stimulated with recombinant CXCL13 for 24 h. The relative levels of HGF mRNA transcripts were measured (n = 3). Statistical significance was made with the the ANOVA test. **I** The relative levels of HGF protein expression were measured, and the HGF band intensities were quantified by densitometry (n = 3). Statistical significance was made with the ANOVA test. **J** BMDMs were transfected with siNC or siFoxO3a for 24 h, were then treated with IL-4 for 2 h and subsequently stimulated with recombinant CXCL13 for 24 h. The relative levels of HGF mRNA transcripts were measured (n = 3). Statistical significance was made with the ANOVA test. **K** The relative levels of HGF protein expression were measured, and the HGF band intensities were quantified by densitometry (n = 3). Statistical significance was made with the ANOVA test. **L** BMDMs were pretreated with IL-4 for 2 h and then stimulated with recombinant CXCL13 for 12 h. Western blot analysis of the nuclear and cytoplasmic levels of FoxO3a (n = 3). Statistical significance was made with the Mann–Whitney U test. **M** ChIP analysis of FoxO3a occupancy at HGF promoter fragments (n = 3). Statistical significance was made with the Student’s t test. **N** BMDMs were pretreated with IL-4 with or without SC79 for 2 h and then stimulated with recombinant CXCL13 for 1 h. The levels of AKT and FoxO3a phosphorylation were detected, and the pAKT/AKT and pFoxO3a/FoxO3a band intensities were quantified by densitometry (n = 3). Statistical significance was made with the ANOVA test. **O** Relative levels of HGF mRNA transcripts in BMDMs treated as indicated. Statistical significance was made with the ANOVA test. **P** The relative levels of HGF protein expression were measured, and the HGF band intensities were quantified by densitometry (n = 3). Statistical significance was made with the ANOVA test.

To further explore how CXCL13 signaling regulates HGF expression during liver regeneration, RNA sequencing (RNA-seq) analysis was performed on hepatic macrophages from *Cxcl13*^*−/−*^ and WT livers after 2/3 PHx. KEGG analysis of the differentially expressed genes revealed that AKT and FoxO signaling pathways were significantly enriched in the *Cxcl13*^*−/−*^ group compared with the WT group (Fig. 6A). Thus, we measured the levels of key phosphorylation-dependent signaling pathways by western blot analyses. Significant increases in p-AKT and p-FoxO3a were observed in hepatic macrophages from *Cxcl13*^*−/−*^ livers compared with those from WT livers after 2/3 PHx (Fig. 6B). To further confirm the effects of CXCL13 on AKT and FoxO3a signaling in vitro, IL-4-activated reparative macrophages were stimulated with recombinant CXCL13. In contrast, western blotting analysis demonstrated that the expression levels of p-AKT and p-FoxO3a were decreased in IL-4-activated reparative macrophages treated with recombinant CXCL13 (Fig. 6C). Moreover, the mRNA and protein levels of HGF were decreased in IL-4-activated macrophages after recombinant CXCL13 treatment (Fig. 6D, E). Furthermore, we observed that the levels of p-AKT and p-FoxO3a were also increased after 2/3 PHx in *Cxcr5 cKO* hepatic macrophages (Fig. 6F). To determine whether CXCL13, through CXCR5, directly triggers macrophages to influence AKT/FoxO3a activity and HGF expression, we treated IL-4-activated reparative macrophages from Cxcr5^F/F^ and *Cxcr5 cKO* mice with recombinant CXCL13. The data revealed that CXCL13 dramatically decreased p-AKT and p-FoxO3a protein levels and the mRNA and protein levels of HGF in the Cxcr5^F/F^ group, but not in *Cxcr5 cKO* group (Fig. 6G–I). These results suggest that CXCL13/CXCR5 signaling is critical for the AKT/FoxO3a signaling axis and HGF production by reparative macrophages.

We next determined whether CXCL13 inhibited HGF expression through the AKT/FoxO3a axis. siRNA was used to knock down FoxO3a expression to evaluate the role of FoxO3a in CXCL13-induced inhibition of HGF expression. We knocked down FoxO3a and found that CXCL13 could dramatically inhibit the mRNA and protein levels of HGF in the Si-NC cells but not the FoxO3a knockdown (Si-FoxO3a) cells (Fig. 6J, K). Furthermore, CXCL13 caused the nuclear translocation of FoxO3a in IL-4-activated macrophages (Fig. 6L). FoxO3a has also been reported to negatively regulate reparative cytokine expression [28, 29]. Indeed, we performed a ChIP assay and found that CXCL13 significantly increased the binding of FoxO3a to the HGF promoter (Fig. 6M). AKT participates in the regulation of FoxO3a phosphorylation and nuclear localization [30]. We used the a unique specific AKT activator SC79 to hyperactivate AKT. Our data showed that SC79 treatment reversed the inhibitory effects of CXCL13 on p-AKT and p-FoxO3a levels (Fig. 6N). Moreover, SC79 reversed the inhibition of HGF mRNA and protein expression induced by CXCL13 treatment in IL-4-activated macrophages (Fig. 6O, P). Collectively, these results demonstrated that CXCL13 suppresses HGF expression in reparative macrophages via CXCR5-mediated AKT/FoxO3a signaling.

### Activation of noncanonical NF-κB induces CXCL13 expression in macrophages to inhibit liver regeneration

**Fig. 7:**
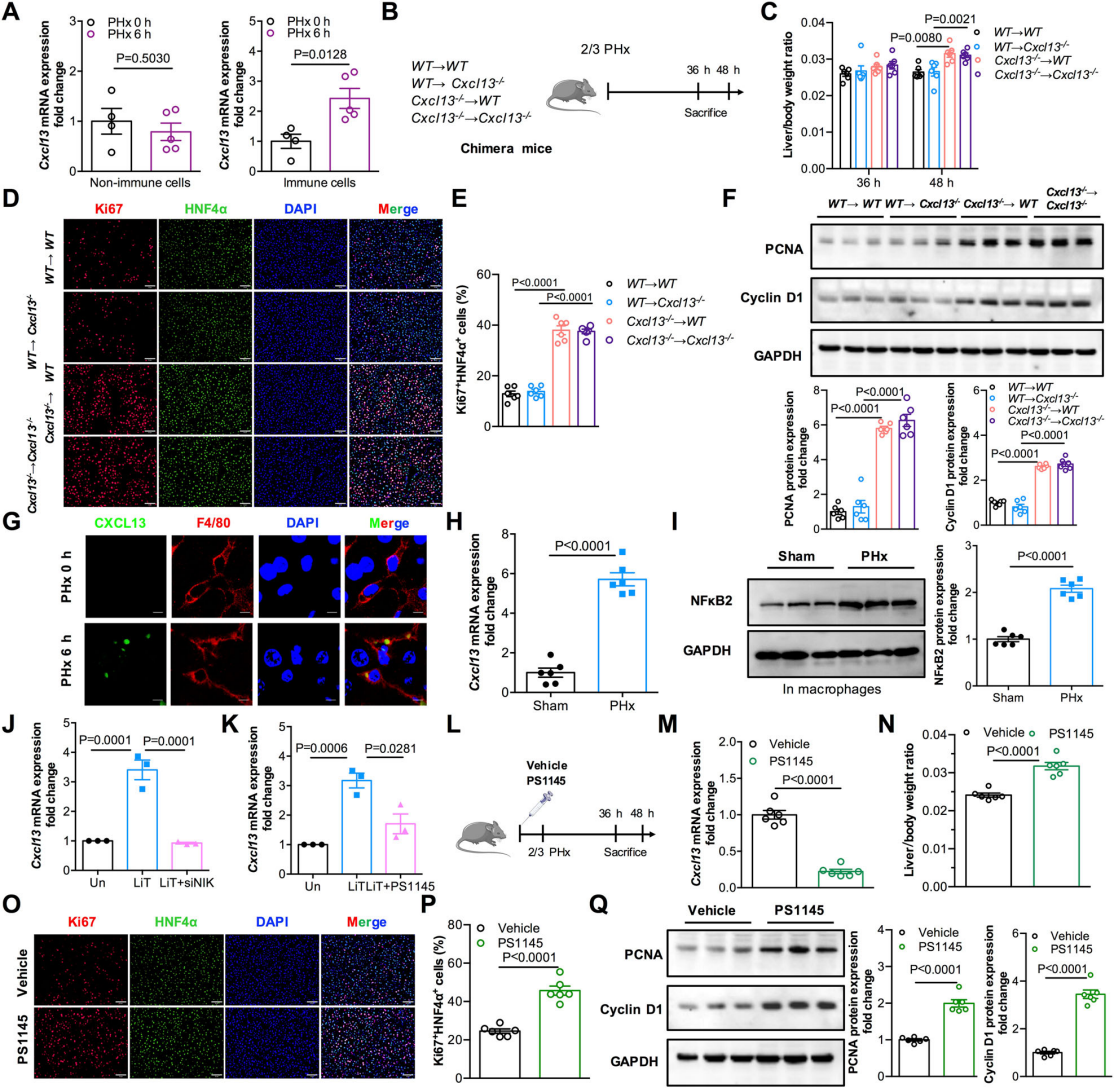
Activation of noncanonical NF-κB induces CXCL13 expression in macrophages to inhibit liver regeneration. **A** Relative levels of CXCL13 mRNA transcripts in immune cells and nonimmune cells isolated from mouse livers after 2/3 PHx at 0 h and 6 h (PHx at 0 h, n = 4; PHx at 6 h, n = 5). Statistical significance was made with the Student’s t test. **B** Diagram of the experimental model of chimeric bone marrow mice with 2/3 PHx. **C** Liver/body weight ratios in chimeric mice at 36 h and 48 h (n = 6). Statistical significance was made with the ANOVA test. **D**, **E** Representative immunofluorescence images of liver Ki67^+^HNF4α^+^ cells in chimeric mice at 36 h and quantification of the percentage of Ki67^+^HNF4α^+^ cells (n = 6). Statistical significance was made with the ANOVA test. **F** Western blot analysis of PCNA and Cyclin D1 expression levels in the livers of chimeric mice after 2/3 PHx, and the band intensity was quantified by densitometry (n = 6). Statistical significance was made with the ANOVA test. **G** Representative immunofluorescence images of CXCL13 (green) and F4/80 (red) in mouse livers after 2/3 PHx. **H** Relative levels of CXCL13 mRNA transcripts in hepatic macrophages after 2/3 PHx (n = 6). Statistical significance was made with the Student’s t test. **I** Western blot analysis of NF-κB2 expression in hepatic macrophages after 2/3 PHx, and the band intensity was quantified by densitometry (n = 6). Statistical significance was made with the Student’s t test. **J** BMDMs transfected with NC siRNA or NIK siRNA were stimulated with LTα1β2 for 24 h, and the relative levels of CXCL13 mRNA were detected (n = 3). Statistical significance was made with the ANOVA test. **K** BMDMs were pretreated with or without PS1145 and then stimulated with LTα1β2 for 24 h, the relative levels of CXCL13 mRNA were detected (n = 3). Statistical significance was made with the ANOVA test. **L** Diagram of the experimental model of PS1145 administration in mice subjected to 2/3 PHx. **M** Relative levels of CXCL13 mRNA at 24 h in livers from mice treated with or without PS1145 after 2/3 PHx (n = 6). Statistical significance was made with the Student’s t test. **N** Liver/body weight ratios were determined in mice treated with or without PS1145 after 2/3 PHx (n = 6). Statistical significance was made with the Student’s t test. **O**, **P** Representative immunofluorescence costaining images of Ki67^+^HNF4α^+^ liver cells in the indicated mice at 36 h after 2/3 PHx and quantification of the percentage of Ki67^+^HNF4α^+^ cells (n = 6). Statistical significance was made with the Student’s t test. **Q** Western blot analysis of PCNA and Cyclin D1 expression levels in liver tissues from mice treated with or without PS1145 at 36 h after 2/3 PHx, and the band intensity was quantified by densitometry (n = 6). Statistical significance was made with the Student’s t test.

To define the cellular source of CXCL13 during liver regeneration, we isolated immune and nonimmune cells from mouse livers after PHx to detect CXCL13 expression. The result showed that CXCL13 was induced in immune cells (Fig. 7A). To further determine whether bone marrow-derived CXCL13 is responsible for its role in liver regeneration, WT → WT, WT→ *Cxcl13*^*−/−*^, *Cxcl13*^*−/−*^→WT, *Cxcl13*^*−/−*^→*Cxcl13*^*−/−*^ chimeric mice were subjected to 2/3 PHx (Fig. 7B and Supplementary Fig. 5A). Compared with WT → WT mice, *Cxcl13*^*−/−*^→*Cxcl13*^*−/−*^ mice retained greater liver/body weight ratios and increased hepatocyte proliferation. Interestingly, *Cxcl13*^*−/−*^→WT mice displayed more rapid liver recovery than WT → WT mice did, whereas WT→ *Cxcl13*^*−/−*^ mice exhibited slower liver recovery than *Cxcl13*^*−/−*^→*Cxcl13*^*−/−*^ mice did (Fig. 7C–F,and Supplementary Fig. 5B). These results strongly suggest that immune cells are important CXCL13 producers. More importantly, elevated CXCL13 expression was observed mainly in F4/80^+^ macrophages after 2/3 PHx (Fig. 7G). Consistently, increased levels of CXCL13 were detected in hepatic macrophages after 2/3 PHx (Fig. 7H). These data indicate that hepatic macrophages may represent an important source of CXCL13 after PHx.

We next investigated the molecular mechanism by which CXCL13 was upregulated in hepatic macrophages after 2/3 PHx. To address whether the transcription factors involved in CXCL13 expression in hepatic macrophages, we examined the transcription factors known to the CXCL13 promoter, including *Hif1α*, *Nf-κb1*, *Nf-κb2* and *Sp1* [31–34]. The results revealed that only *Nf-κb2* was significantly enhanced in hepatic macrophages after PHx (Supplementary Fig. 5C). Moreover, the protein levels of NF-κB2 were elevated in hepatic macrophages and liver tissues after 2/3 PHx (Fig. 7I and Supplementary Fig. 5D). LTα1β2 is known to activate noncanonical NF-κB2 [35]. Indeed, LTα1β2 treatment markedly augmented the levels of NF-κB2 and CXCL13 expression in macrophages (Supplementary Fig. 5E, F). We next determined whether noncanonical NF-κB2 activation is involved in the induction of CXCL13 expression. NF-κB-inducing kinase (NIK) is required for the activation of the noncanonical NF-κB2 pathway [36], NIK knockdown inhibited the expression of CXCL13 induced by LTα1β2 in macrophages (Fig. 7J). In parallel, the induction of CXCL13 expression could be abrogated by PS1145, an NF-κB inhibitor (Fig. 7K). These results indicate that the activation of noncanonical NF-κB signaling is responsible for the upregulation of CXCL13 in hepatic macrophages.

To explore the roles of activated NF-κB in CXCL13 expression and liver regeneration in vivo, the mice were treated with PS1145 and then subjected to 2/3 PHx (Fig. 7L). These results indicated that PS1145 administration suppressed CXCL13 expression in liver tissues after 2/3 PHx (Fig. 7M). Furthermore, PS1145 administration significantly promoted liver regeneration after 2/3 PHx, as evidenced by a significantly greater liver/body weight ratios (Fig. 7N), a greater percentage of Ki67^+^HNF4α^+^ cells (Fig. 7O, P), higher PCNA and Cyclin D1 expression levels (Fig. 7Q), and lower ALT and AST levels (Supplementary Fig. 5G), as compared to those in the vehicle group. Therefore, these results demonstrate that noncanonical NF-κB signaling activation leads to increased CXCL13 production in hepatic macrophages to inhibit liver regeneration.

### Neutralization of CXCL13 promotes liver regeneration after PHx

**Fig. 8:**
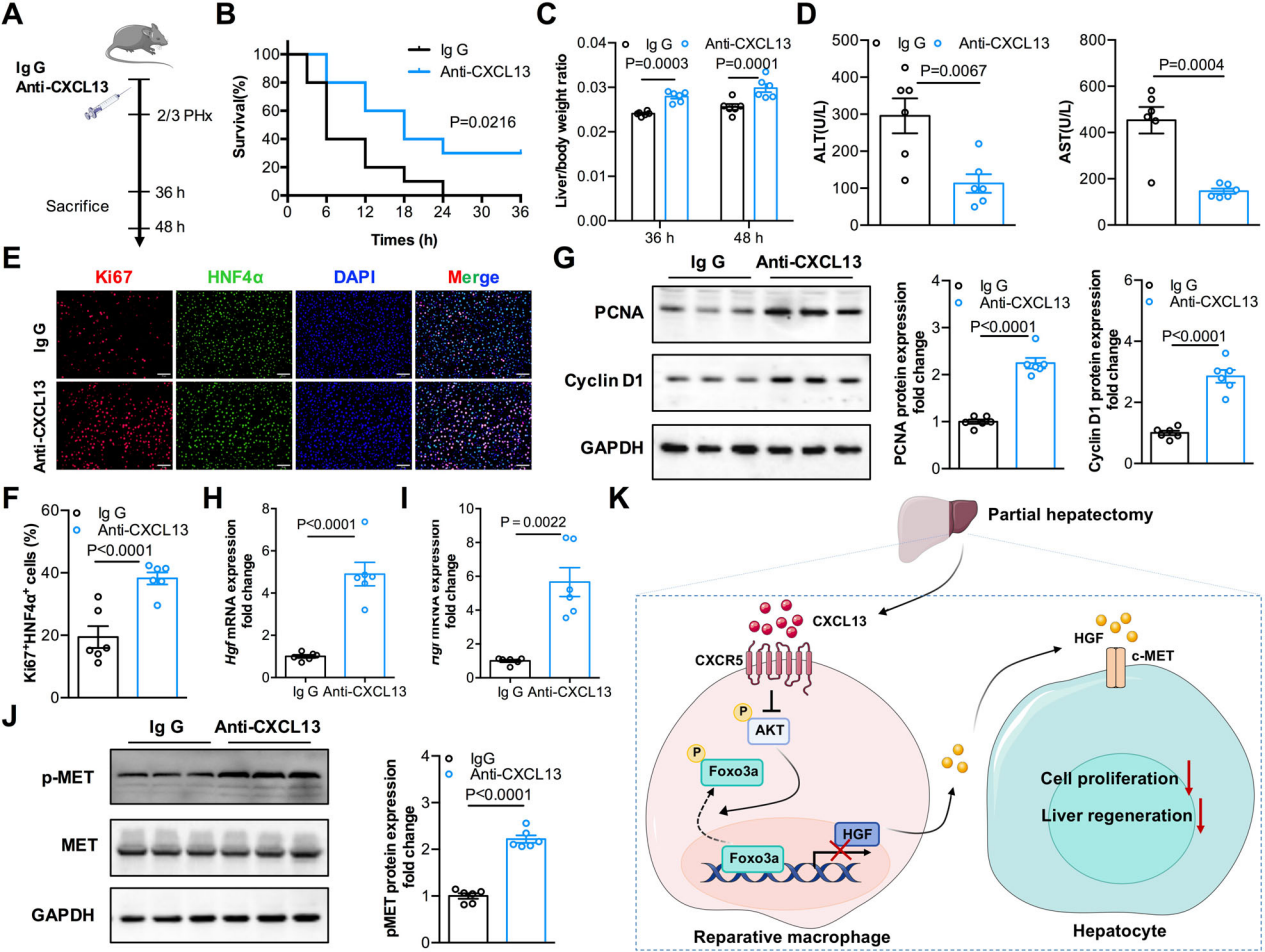
Neutralization of CXCL13 promotes liver regeneration after PHx. **A** Diagram of the experimental PHx model in which mice were treated with CXCL13 neutralization antibody (Anti-CXCL13) or Ig G. **B** Survival curves of mice treated with anti-CXCL13 or Ig G after lethal 85% PHx (Ig G, n = 10; Anti-CXCL13, n = 10). Statistical significance was made with the Log rank test. **C** Liver/body weight ratios of the mice treated with the anti-CXCL13 or Ig G at 36 and 48 h after 2/3 PHx (n = 6). Statistical significance was made with the ANOVA test. **D** Serum ALT and AST levels in mice treated with the anti-CXCL13 or Ig G at 36 h after 2/3 PHx (n = 6). Statistical significance was made with the Student’s t test. **E**, **F** Representative immunofluorescence images of Ki67^+^HNF4α^+^ liver cells in the indicated mice at 36 h after 2/3 PHx and quantification of the percentage of Ki67^+^HNF4α^+^ cells (n = 6). Statistical significance was made with the Student’s t test. **G** Western blot analysis of PCNA and Cyclin D1 expression levels in the liver tissues of the mice treated as indicated, and the band intensity was quantified by densitometry (n = 6). Statistical significance was made with the Student’s t test. **H** Relative levels of HGF mRNA transcripts in hepatic macrophages from mice treated as indicated at 36 h after 2/3 PHx (n = 6). Statistical significance was made with the Student’s t test. **I** The relative levels of HGF mRNA transcripts in livers from mice treated with anti-CXCL13 or Ig G at 36 h after 2/3 PHx (n = 6). Statistical significance was made with the Mann–Whitney U test. **J** Western blot analysis of pMET expression in liver tissues of mice treated as indicated after 2/3 PHx, and the band intensity was quantified by densitometry (n = 6). Statistical significance was made with the Student’s t test. **K** Working model of CXCL13 in liver regeneration.

To evaluate the potential application of CXCL13 blockade in liver regeneration, an anti-CXCL13 neutralizing antibody (anti-CXCL13) was administered to PHx model mice (Fig. 8A). Notably, anti-CXCL13 treatment significantly increased the survival rate in the 85% PHx model (Fig. 8B). In the 2/3 PHx model, anti-CXCL13 treatment increased the liver/body weight ratios and reduced the serum levels of ALT and AST (Fig. 8C, D). Compared with those in the control treatment group, mice treated with anti-CXCL13 displayed an increased number of Ki67^+^HNF4α^+^ cells at 36 h after 2/3 PHx (Fig. 8E, F). Consistently, the expression levels of PCNA and Cyclin D1 were also elevated after anti-CXCL13 treatment (Fig. 8G). Moreover, we observed that the levels of HGF expression was significantly greater in hepatic macrophages from anti-CXCL13-treated mice than in those from control mice after 2/3 PHx (Fig. 8H). In addition, anti-CXCL13-treated mice presented higher HGF levels and significantly greater c-MET phosphorylation levels in liver tissues after 2/3 PHx than did the control group (Fig. 8I, J). The above results further confirmed that blockade of CXCL13 promoted liver regeneration after PHx.

## Discussion

Liver regeneration is essential for patients who have undergone donor liver transplantation and partial surgical hepatectomy. Defective hepatocyte regeneration results in severe liver dysfunction and liver failure [2, 37]. Therefore, identifying the adaptor molecules that regulate liver regeneration might provide therapeutic strategies to decrease the incidence of postoperative liver failure. In our study, we demonstrated that CXCL13 signaling delayed liver regeneration through the HGF/c-MET-involved hepatic macrophage‒hepatocyte axis (Fig. 8K). Increased levels of CXCL13 were observed in the PHx mouse model and in patients who underwent liver resection. We further showed that CXCL13 deficiency promoted liver regeneration in PHx mice and CCl_4_-induced liver injury. Mechanistically, CXCL13 inhibits HGF expression in reparative macrophages by regulating CXCR5-mediated FoxO3a signaling, leading to inactivation of HGF/c-MET signaling in hepatocytes and delaying liver regeneration. We further revealed that the activation of noncanonical NF-κB induced CXCL13 expression in macrophages. Importantly, neutralization of CXCL13 accelerated liver regeneration and improved the survival of mice after PHx. These findings reveal the role and underlying mechanisms of CXCL13 in liver regeneration and suggest that targeting CXCL13 signaling decreases the risk of insufficient liver regeneration.

### CXCL13 delays liver regeneration

One of the most important findings of this study was that CXCL13 is a negative factor in regulating liver regeneration. Our interpretation was based on several lines of evidence. First, CXCL13 levels were significantly increased in patients following liver resection and PHx mice. Using genetic deletion of CXCL13, we found that CXCL13 deficiency improved liver regeneration after PHx or CCl_4_ injection, as shown by enhanced hepatocyte proliferation and ameliorated liver damage. In contrast, recombinant CXCL13 treatment dramatically decreased hepatocyte proliferation and liver regeneration in *Cxcl13*^*−/−*^ mice after PHx. Moreover, neutralization of CXCL13 significantly promoted liver regeneration after PHx. Together, these findings provide evidence that CXCL13 is a negative regulator of liver regeneration.

CXCL13 has been reported to regulate tumor development, and patients with high CXCL13 levels exhibit poor outcomes [10, 11]. Accordingly, CXCL13 has been shown to promote tumor cell proliferation [38, 39]. However, here we showed that CXCL13 does not influence primary hepatocyte proliferation in vitro, suggesting that CXCL13 does not directly control hepatocyte proliferation. Macrophage polarization plays a critical role in tissue regeneration after injury, and reparative macrophages are associated with enhanced liver regeneration [5, 7, 40, 41]. Ly6C^low^ reparative macrophages produce anti-inflammatory mediators and reparative molecules to enhance liver regeneration and prevent liver failure. Conversely, Ly6C^high^ proinflammatory macrophages secrete proinflammatory cytokines, leading to an inflammatory response and worse outcomes posthepatectomy [6, 42]. The present data revealed that more anti-inflammatory Ly6C^low^ macrophages and fewer proinflammatory Ly6C^high^ macrophages in *Cxcl13*^*−/−*^ mice after PHx. HGF is secreted by nonparenchymal cells, including macrophages, and activation of the HGF/c-MET pathway plays essential roles in hepatic proliferation and liver regeneration [43–45]. In accordance with these studies, we found that HGF expression was increased in reparative macrophages from *Cxcl13*^*−/−*^ livers after PHx. We also demonstrated that the pro-regenerative effects of CXCL13 deficiency were blocked by an HGF neutralizing antibody as well as the c-MET inhibitor crizotinib. These results indicate that CXCL13 deficiency positively regulates the HGF/c-MET pathway, which controls proliferation signal transduction from reparative macrophages to hepatocytes and ultimately contributes to hepatocyte proliferation and liver regeneration.

### CXCL13 downregulates HGF production via CXCR5- mediated FoxO3a signaling

Our data suggested that CXCL13 deficiency enhanced liver regeneration by upregulating HGF expression in reparative macrophages. However, the molecular mechanism by which CXCL13 regulates HGF expression in reparative macrophages remains unclear. RNA-seq revealed that the AKT and FoxO signaling pathways were enriched in reparative macrophages from *Cxcl13*^*−/−*^ livers after PHx. As a transcription factor, FoxO proteins are downstream effectors of AKT that play pivotal roles in the cell cycle, cell survival, differentiation and transformation [46]. FoxO3a is inactivated through phosphorylation by AKT, which results in decreased nuclear accumulation [47]. Notably, we demonstrated that CXCL13 reduced FoxO3a phosphorylation and subsequently promoted FoxO3a translocation to the nucleus. FoxO3a acts as a transcription factor that increases CXCL16 and TNFα expression, thereby promoting inflammation [48, 49]. Conversely, FoxO3a also suppressed the release of reparative cytokines, including IL-10 and VEGF [28, 29]. We further revealed that CXCL13 negatively regulates HGF production in reparative macrophages through an AKT/FoxO3a-dependent pathway.

The CXCR5 receptor is a candidate receptor for CXCL13 that regulates various biological processes, including nerve regeneration, the inflammatory response, autoimmune diseases and cancer [34, 50, 51]. Our study revealed a previously unknown function of macrophage CXCR5 in delaying liver regeneration after PHx and downregulating HGF expression. CXCR5 deficiency in myeloid cells enhanced liver regeneration in a mouse PHx model. Notably, specific depletion of neutrophils did not influence augmented liver regeneration in myeloid CXCR5-deficient mice. Moreover, we found that the overexpression of myeloid CXCR5 significantly delayed liver regeneration. The results revealed that overexpressing CXCL13 in *Cxcl13*^*−/−*^ mice reversed the increase in liver regeneration observed in *Cxcl13*^*−/−*^ mice. However, this effect was not observed in CXCR5-deficient mice, suggesting that CXCL13 delays liver regeneration in a CXCR5-dependent manner. We further demonstrated that CXCR5 is required for CXCL13-induced inhibition of FoxO3a phosphorylation and HGF expression in reparative macrophages. Together, these results demonstrated that CXCL13/CXCR5 decreased HGF expression by regulating the AKT/FoxO3a axis in reparative macrophages.

CXCL13 is produced by several cell types, including epithelial and immune cells [13, 52, 53]. In our study, we revealed that CXCL13 expression was upregulated in immune cells after PHx. Furthermore, bone marrow-derived CXCL13 is responsible for its role in the inhibition of liver regeneration. We provide evidence that hepatic macrophages are responsible for CXCL13 expression in a mouse PHx model. NF-κB signaling has been shown to induce CXCL13 expression, which in turn affects neurological repair and prostate tumorigenesis [32, 34]. Similarly, in the present study, we found that the activation of noncanonical NF-κB elevates the production of CXCL13 by macrophages. We also found that treatment with the NF-κB inhibitor PS1145 decreased CXCL13 expression both in vitro and in vivo. As expected, we also revealed that the administration of PS1145 significantly facilitated liver regeneration in mice after PHx. Thus, CXCL13 has been identified as a key regulator that links noncanonical NF-κB activation to liver regeneration. However, the contribution of the cell-specific function of CXCL13 to liver regeneration requires further investigation in CXCL13 conditional knockout mice.

In summary, the present study revealed that CXCL13 functions as an important negative regulator of liver regeneration that links hepatic macrophages and hepatocyte proliferation. Further mechanistic exploration revealed that CXCL13 inhibited HGF expression in reparative macrophages via the CXCR5-mediated AKT/FoxO3a signaling axis, resulting in decreased activation of c-MET signaling in hepatocytes, thereby suppressing hepatocyte proliferation and liver regeneration. Notably, neutralization of CXCL13 effectively improved liver regeneration after PHx. Our findings suggest that targeting CXCL13 signaling might be a potential therapeutic strategy for hepatectomized patients with insufficient liver regeneration.

## Supplementary information

Supplemental Material

Raw wb images

original results

## Data Availability

The data generated or analyzed during this study are included in this published article and its supplementary information files.
