# Correction: CXCL13 suppresses liver regeneration through the negative regulation of HGF signaling

**DOI:** 10.1038/s41419-025-07761-3

**Published:** 2025-07-29

**Authors:** Qun Zhao, Jingyi Wu, Mengyuan Feng, Anjie Zhang, Liwei Fu, Jinglin Chen, Lian Li, Fangzhou Li, Tingting Li, Shu Jin, Shengbao Li, Xianjun Yu

**Affiliations:** 1 Department of Gastroenterology, Taihe Hospital, School of Basic Medical Sciences, Hubei Key Laboratory of Embryonic Stem Cell Research, Hubei University of Medicine, Shiyan, China; 2 Laboratory of Inflammation and Molecular Pharmacology, Biomedical Research Institute, Inflammation-Cancer Transformation and Wudang Chinese Medicine Research, Hubei Talent Introduction and Innovation Demonstration Base, Hubei Provincial Clinical Research Center for Umbilical Cord Blood Hematopoietic Stem Cells, Hubei University of Medicine, Shiyan, China; 3 Department of Clinical Laboratory, Renmin Hospital, Hubei University of Medicine, Shiyan, China

**Keywords:** Cell signalling, Medical research

Correction to: *Cell Death and Disease* 10.1038/s41419-025-07568-2, published online 5 May 2025

During the production process Xianjun Yu has been erroneously processed as the only corresponding author.

Xianjun Yu and Shengbao Li are the two originally provided corresponding authors.

The original article has been corrected.

