# Supplementary material for: CXCL13 suppresses liver regeneration through the negative regulation of HGF signaling
Source: Cell Death Dis. 2025 May 5;16(1):361. doi: 10.1038/s41419-025-07568-2 (PMC12052986; doi:10.1038/s41419-025-07568-2)
Supplement: Supplementary file 1 — Supplemental Material [file 41419_2025_7568_MOESM1_ESM.docx]

**Supplementary Information**

**CXCL13 suppresses liver regeneration through the negative regulation of HGF signaling**

Qun Zhao ^1,2 *^, Jingyi Wu ^1,2 *^, Mengyuan Feng ^1,2 *^, Anjie Zhang ^1,2^, Liwei Fu ^1,2^, Jinglin Chen ^3^, Lian Li ^3^, Fangzhou Li ^2^, Tingting Li ^2^, Shu Jin ^1^, Shengbao Li ^1^ ✉, Xianjun Yu ^1,2^ ✉

**Supplementary Figures**

**Supplementary Fig. 1** Deficiency of CXCL13 accelerates liver regeneration after PHx

**Supplementary Fig. 2** CXCL13 delays liver regeneration by inhibiting HGF expression

**Supplementary Fig. 3** CXCL13 deficiency enhances reparative Ly6C^low^ macrophage polarization after PHx

**Supplementary Fig. 4** CXCR5 delays liver regeneration after PHx

**Supplementary Fig. 5** CXCL13 expression in hepatic macrophages requires activation of NF-κB

**
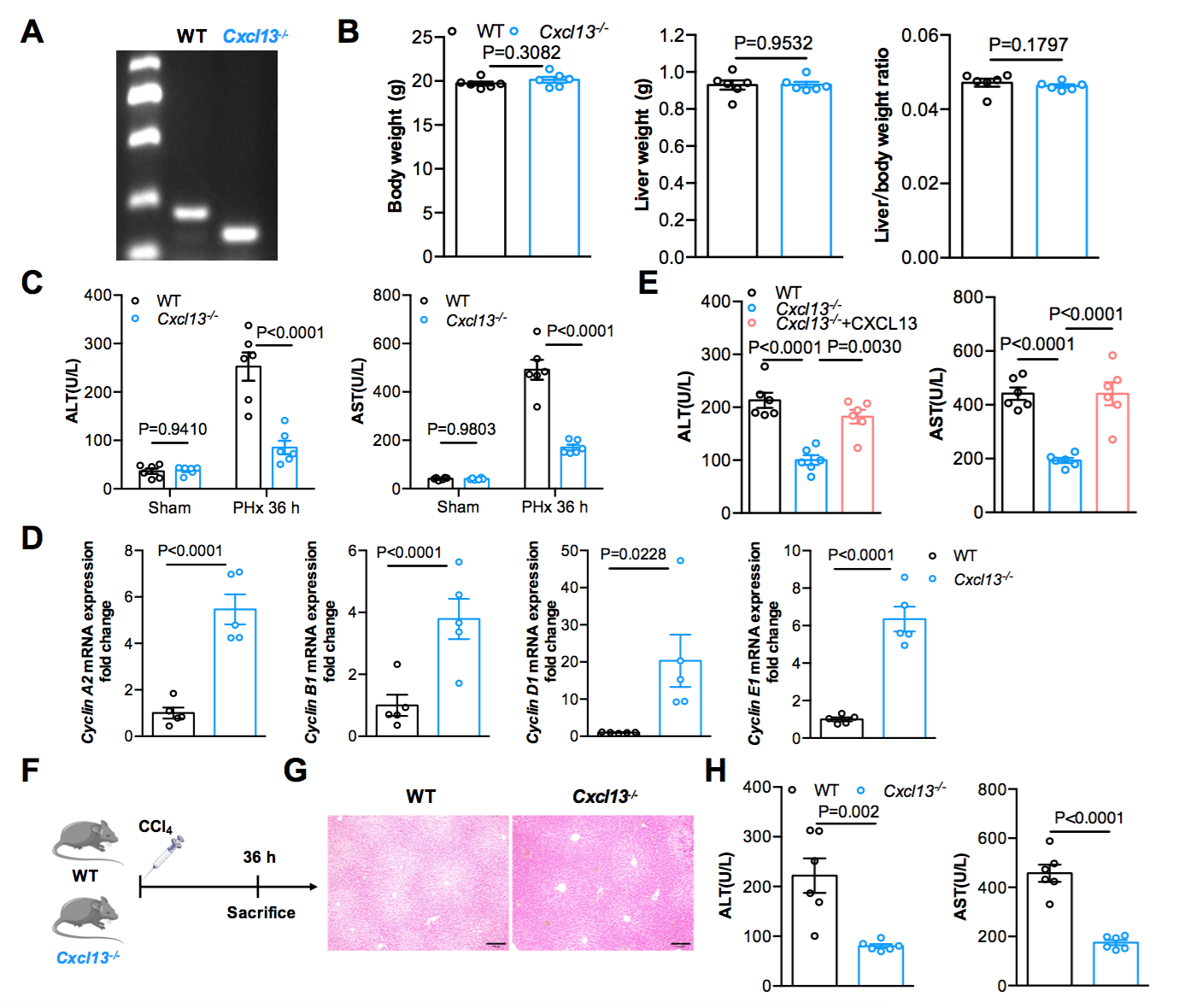
**

**Supplementary Fig. 1 Deficiency of CXCL13 accelerates liver regeneration after PHx**

**A** Genotypes of WT and *Cxcl13^-/-^* mice were determined by PCR amplification. **B** Body weights, liver weights and liver/body weight ratios in WT and *Cxcl13^-/-^* mice (n=6). Statistical significance was made with the Student's t test in body weights and liver weights, and the Mann-Whitney U test in liver/body weight ratios. **C** Serum ALT and AST levels in WT and *Cxcl13^-/-^* mice treated as indicated (n=6). Statistical significance was made with the ANOVA test. **D** Relative levels of Cyclin mRNA in WT and *Cxcl13^-/-^* livers at 36 h after 2/3 PHx (n=5). Statistical significance was made with the Mann-Whitney U test in Cyclin A, Cyclin D, Cyclin E mRNA, and the Student's t test in Cyclin B mRNA. **E** Serum ALT and AST levels in WT and *Cxcl13^-/-^* mice treated with or without recombinant CXCL13 at 36 h after 2/3 PHx (n=6). Statistical significance was made with the ANOVA test. **F** Diagram of the experimental model of liver injury in WT and *Cxcl13^-/-^* mice induced by CCl_4_. **G** Representative images of H&E staining of the livers of mice after CCl_4_ injection. **H** Serum ALT and AST levels in WT and *Cxcl13^-/-^* mice after CCl_4_ injection (n=6). Statistical significance was made with the Mann-Whitney U test for ALT and the Student's t test for AST.

**
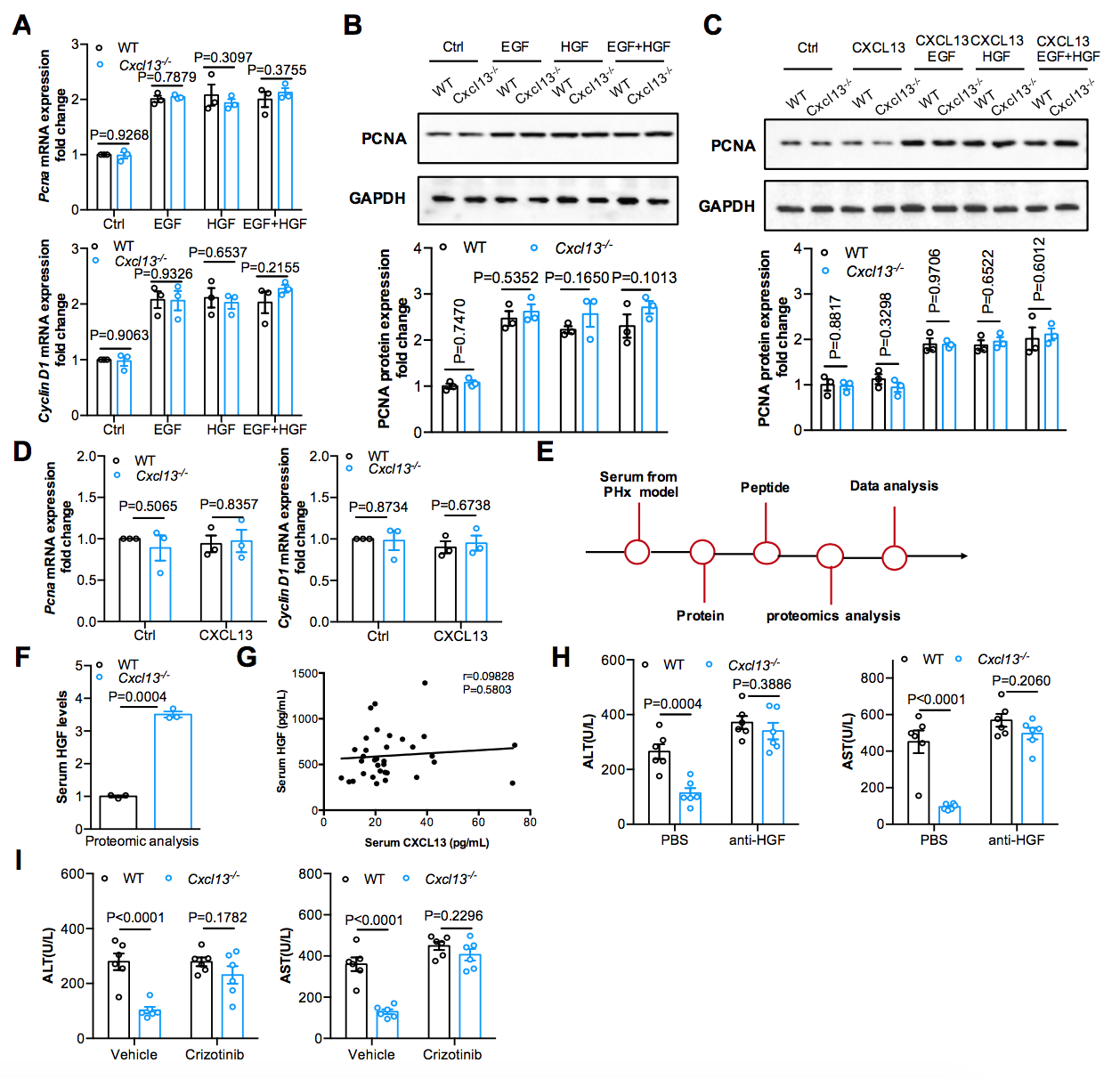
**

**Supplementary Fig. 2 CXCL13 delays liver regeneration by inhibiting HGF expression**

**A** WT and *Cxcl13^-/-^* primary hepatocytes were incubated with EGF or HGF and costimulated for 48 h. The relative levels of PCNA and Cyclin D1 mRNA were measured (n=3). Statistical significance was made with the ANOVA test. **B** WT and *Cxcl13^-/-^* primary hepatocytes were incubated with EGF or HGF and costimulated for 48 h. The relative levels of PCNA protein were measured, and the band intensity was quantified by densitometry (n=3). Statistical significance was made with the ANOVA test. **C** WT and *Cxcl13^-/-^* primary hepatocytes were incubated with recombinant CXCL13, EGF and HGF for 48 h, after which the protein levels of PCNA were detected, and the band intensity was quantified by densitometry (n=3). Statistical significance was made with the ANOVA test. **D** WT and *Cxcl13^-/-^* primary hepatocytes were incubated with recombinant CXCL13. The relative levels of PCNA and Cyclin D1 mRNA were measured (n=3). Statistical significance was made with the ANOVA test. **E** Schematic diagram of the process of serum proteomics analysis. **F** Serum HGF protein levels obtained from serum proteomics (n=3). Statistical significance was made with the Student's t test. **G** The correlations between the serum CXCL13 and HGF levels in patients pre-LR were evaluated by Spearman correlation analysis. Correlations were calculated by the Spearman correlation coefficient (n=34). **H** Serum ALT and AST levels in WT and *Cxcl13^-/-^* mice treated with or without HGF antibodies at 36 h after 2/3 PHx (n=6). Statistical significance was made with the ANOVA test. **I** Serum ALT and AST levels in WT and *Cxcl13^-/-^* mice with and without crizotinib treatment at 36 h after 2/3 PHx (n=6). Statistical significance was made with the ANOVA test.

**
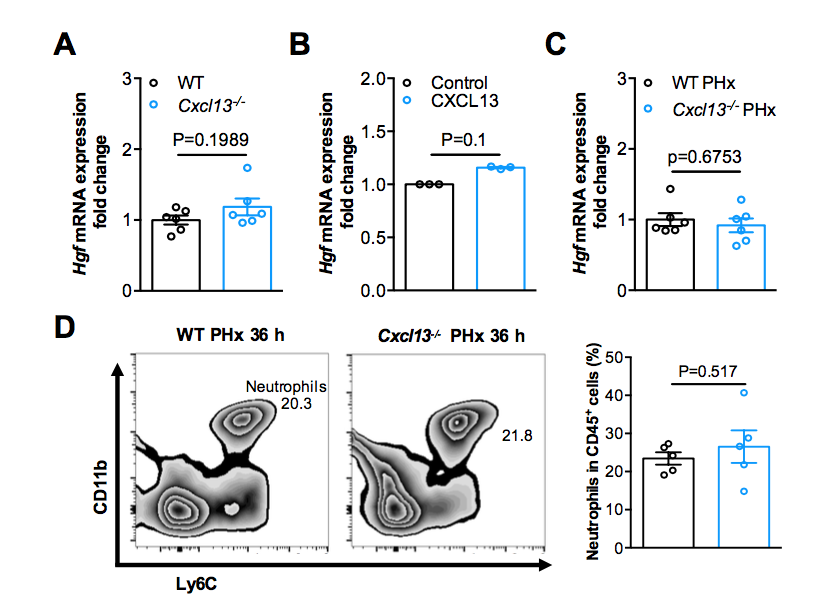
**

**Supplementary Fig. 3 CXCL13 deficiency enhances reparative Ly6C^low^ macrophage polarization after PHx**

**A** Relative levels of HGF mRNA transcripts in primary hepatocytes from WT and *Cxcl13^-/-^* livers (n=6). Statistical significance was made with the Mann-Whitney U test. **B** Relative levels of HGF mRNA transcripts in primary hepatocytes treated with or without recombinant CXCL13 (50 ng/mL) for 48 h (n=3). Statistical significance was made with the Student's t test. **C** Relative levels of HGF mRNA transcripts in primary hepatocytes from WT and *Cxcl13^-/-^* mice at 36 h after 2/3 PHx (n=6). Statistical significance was made with the Mann-Whitney U test. **D** Representative FACS plots of neutrophils from WT and *Cxcl13^-/-^* mice at 36 h after 2/3 PHx (n=5). Statistical significance was made with the Student's t test.

**
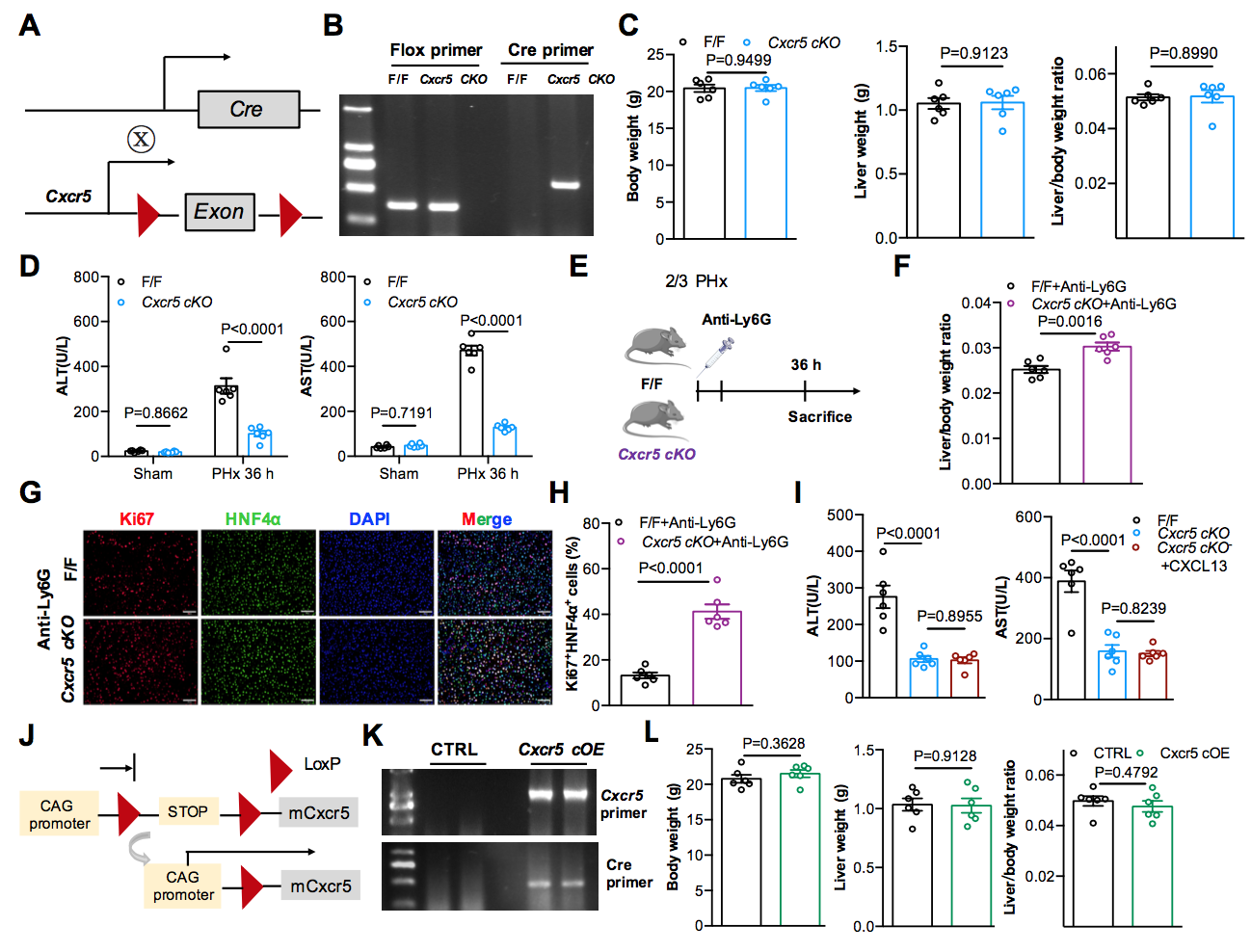
**

**Supplementary Fig. 4 CXCR5 delays liver regeneration after PHx**

**A** Schematic diagram of the generation of LysM-Cre; *Cxcr5^F/F^* mice (*Cxcr5 cKO*). **B** The genotypes of Cxcr5^F/F^ and *Cxcr5 cKO* mice were determined by PCR amplification. **C** Body weights, liver weights and liver/body weight ratios in Cxcr5^F/F^ and *Cxcr5 cKO* mice (n=6). Statistical significance was made with the Student's t test. **D** Serum ALT and AST levels in Cxcr5^F/F^ and *Cxcr5 cKO* mice treated as indicated (n=6). Statistical significance was made with the ANOVA test. **E** Model of the neutrophil depletion assay. Neutrophils from *Cxcr5 cKO* and Cxcr5^F/F^ mice were depleted with an anti-Ly6G antibody, and 2/3 PHx was performed. **F** Liver/body weight ratios in *Cxcr5 cKO* and Cxcr5^F/F^ mice lacking neutrophils (n=6). Statistical significance was made with the Student's t test. **G, H** Representative immunofluorescence images of liver Ki67^+^HNF4α^+^ cells in the indicated mice lacking neutrophils at 36 h after 2/3 PHx. The percentage of Ki67^+^HNF4α^+^ cells was quantified (n=6). Statistical significance was made with the Student's t test. **I** Serum ALT and AST levels in Cxcr5^F/F^ and *Cxcr5 cKO* mice with and without recombinant CXCL13 treatment after 2/3 PHx (n=6). Statistical significance was made with the ANOVA test. **J** Scheme of conditional overexpression of CXCR5 mice. **K** The genotypes of control (CTRL) and *Cxcr5 cOE* mice were determined by PCR amplification. **L** Body weights, liver weights and liver/body weight ratios in CTRL and *Cxcr5 cOE* mice (n=6). Statistical significance was made with the Student's t test.

**
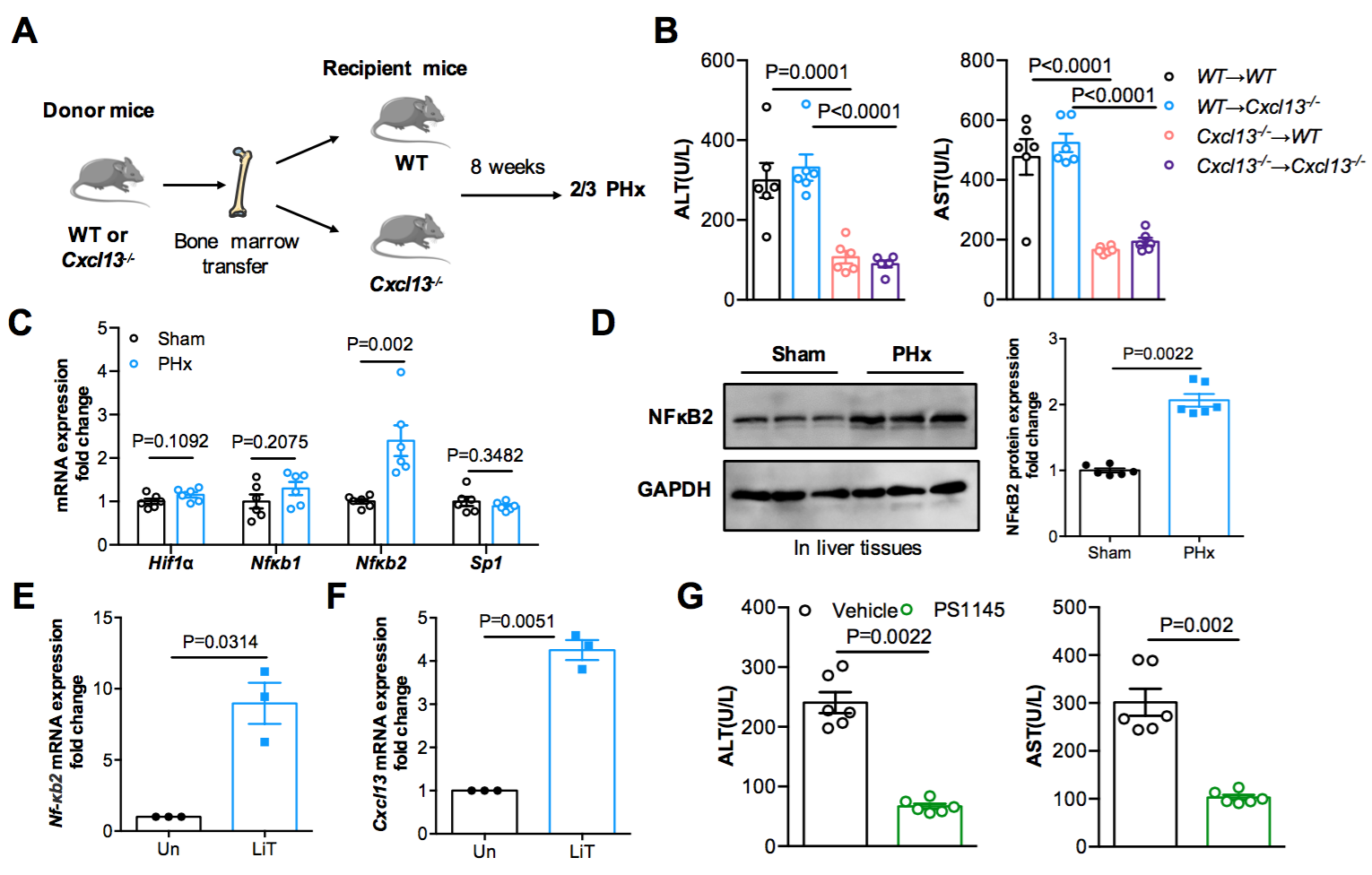
**

**Supplementary Fig. 5 CXCL13 expression in hepatic macrophages requires activation of NF-κB**

**A** Schematic diagram of bone marrow transplantation. **B** Serum ALT and AST levels in bone marrow chimera mice after 2/3 PHx (n=6). Statistical significance was made with the ANOVA test. **C** qPCR analysis of genes expression in hepatic macrophages after 2/3 PHx (n=6). Statistical significance was made with the Student's t test in *Hif1α, Nfκb1, Sp1*, and the Mann-Whitney U test in *Nfκb2*. **D** Western blot analysis of NF-κB2 expression in livers from mice after 2/3 PHx, and the band intensity was quantified by densitometry (n=6). Statistical significance was made with the Mann-Whitney U test. **E, F** BMDMs were stimulated with LTα1β2 for 24 h, and the relative levels of NF-κB2 and CXCL13 were detected (n=3). Statistical significance was made with the Mann-Whitney U test. **G** Serum ALT and AST levels in mice treated as indicated after 2/3 PHx (n=6). Statistical significance was made with the Mann-Whitney U test.
