# Supplementary material for: CXCL13 suppresses liver regeneration through the negative regulation of HGF signaling
Source: Cell Death Dis. 2025 May 5;16(1):361. doi: 10.1038/s41419-025-07568-2 (PMC12052986; doi:10.1038/s41419-025-07568-2)

Fig 1d

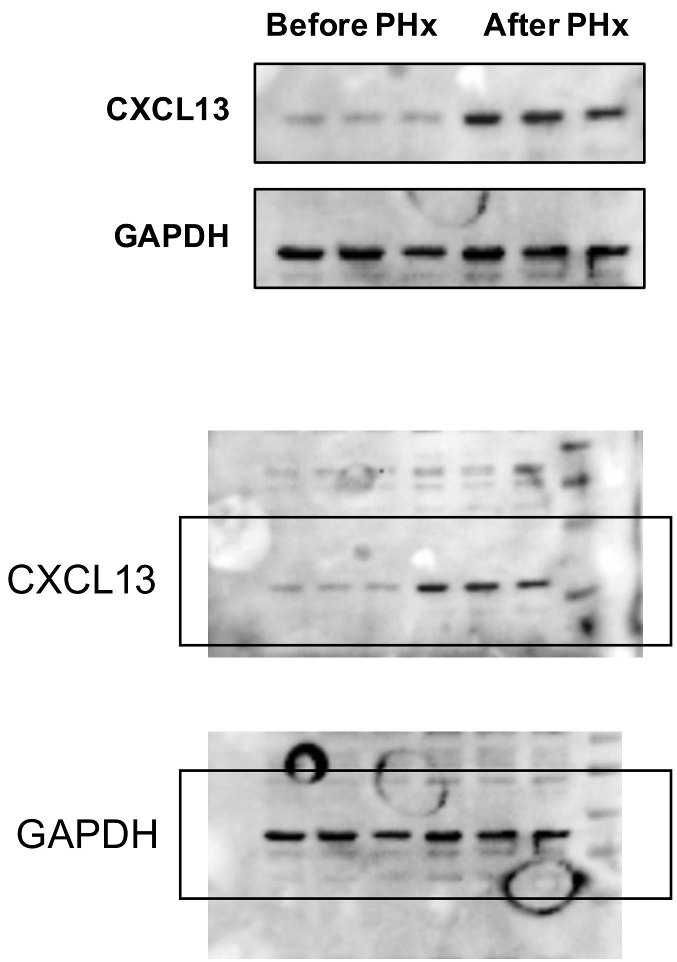

Before PHx
After PHx
CXCL13
GAPDH
CXCL13
GAPDH

Fig 2e

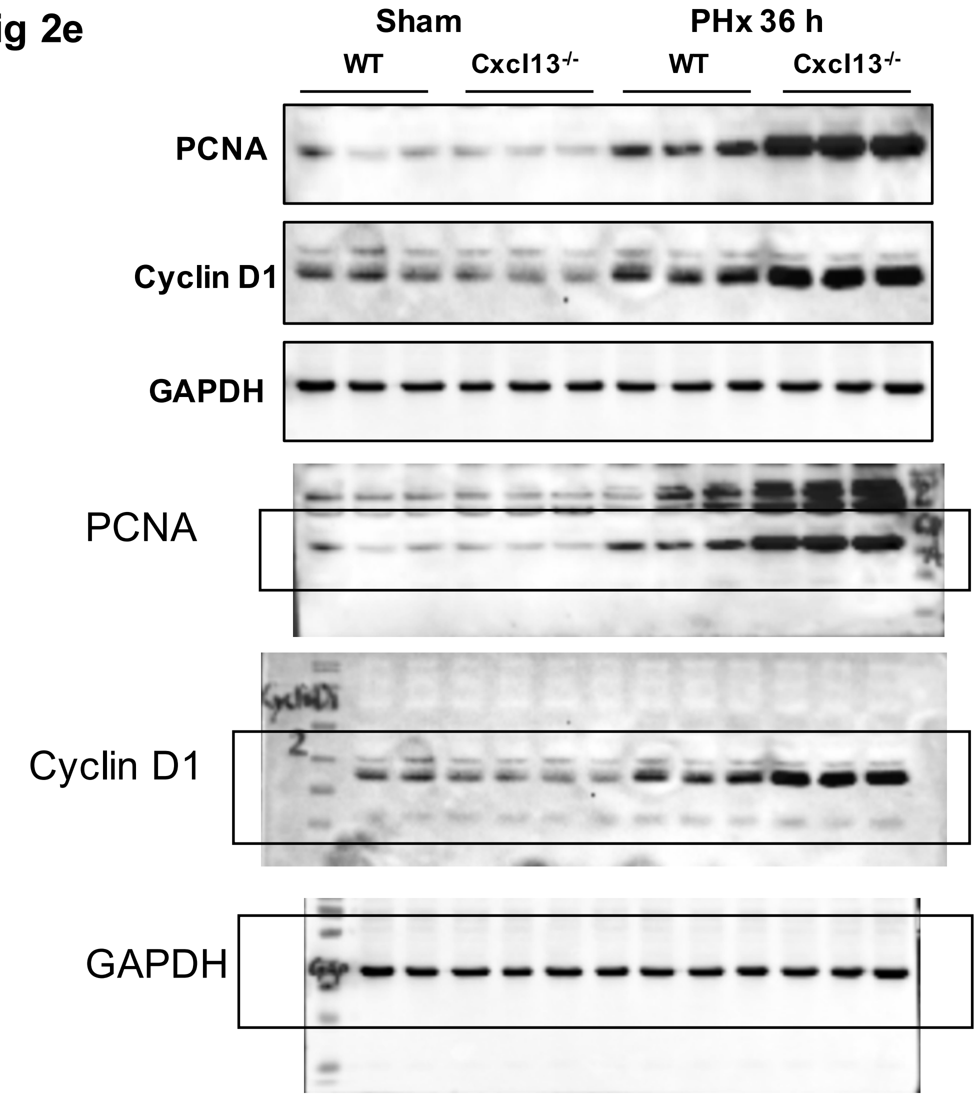

Sham
PHx 36 h
WT
Cxcl13$^{-/-}$
WT
Cxcl13$^{-/-}$
PCNA
Cyclin D1
GAPDH
PCNA
Cyclin D1
GAPDH

Fig 2k

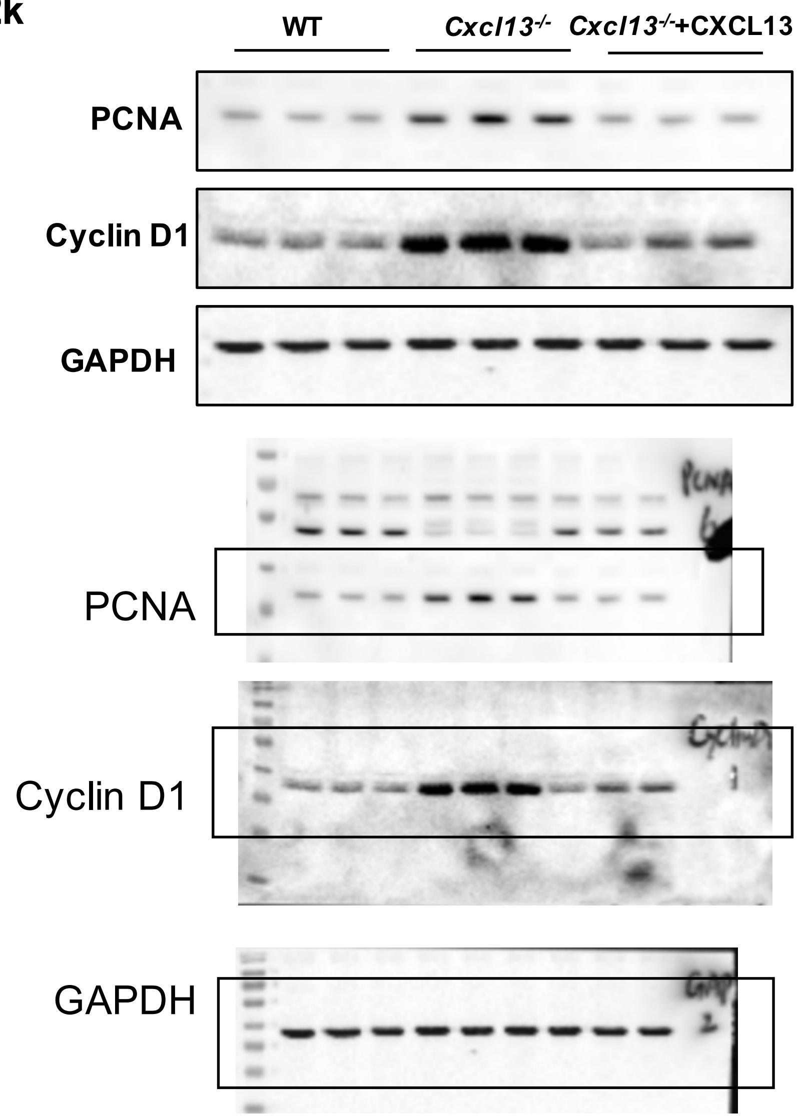
WT
Cxcl13$^{-/-}$
Cxcl13$^{-/-}$+CXCL13
PCNA
Cyclin D1
GAPDH
PCNA
Cyclin D1
GAPDH

Fig. 2l

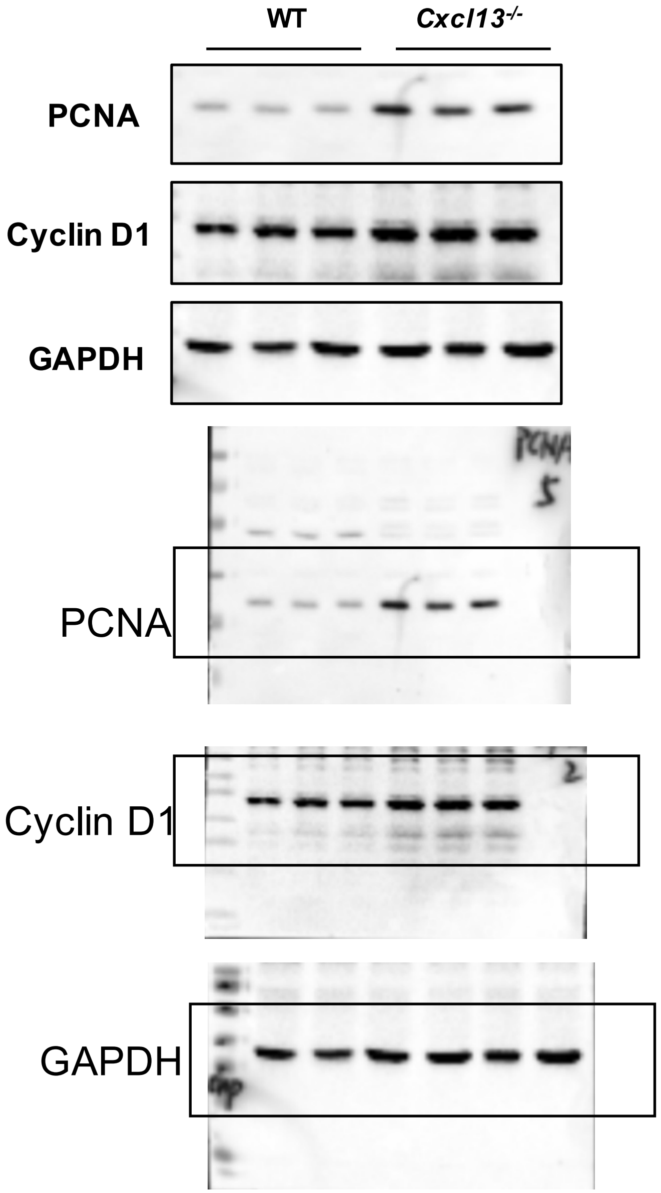
WT
Cxcl13$^{-/-}$
PCNA
Cyclin D1
GAPDH
PCNA
Cyclin D1
GAPDH

Fig. 3h

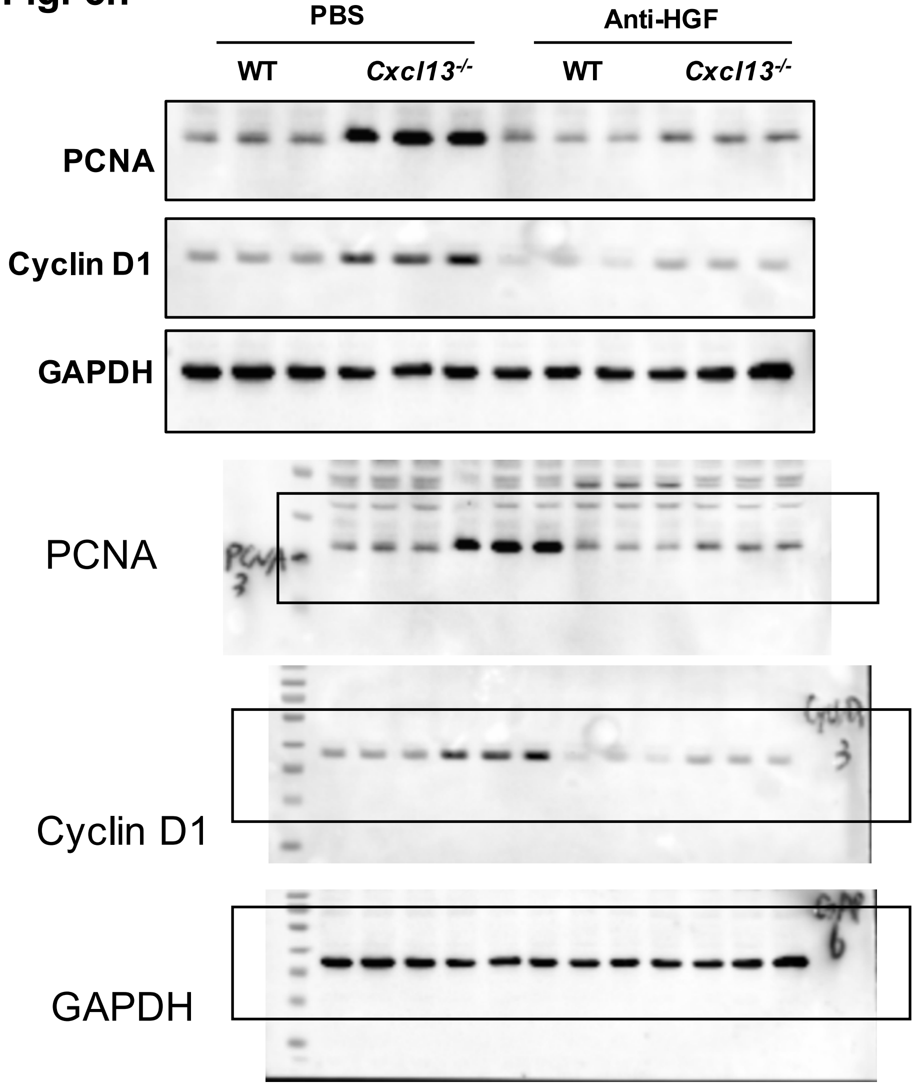
PBS
Anti-HGF
WT
Cxcl13-/-
WT
Cxcl13-/-
PCNA
Cyclin D1
GAPDH
PCNA
Cyclin D1
GAPDH

Fig 3i

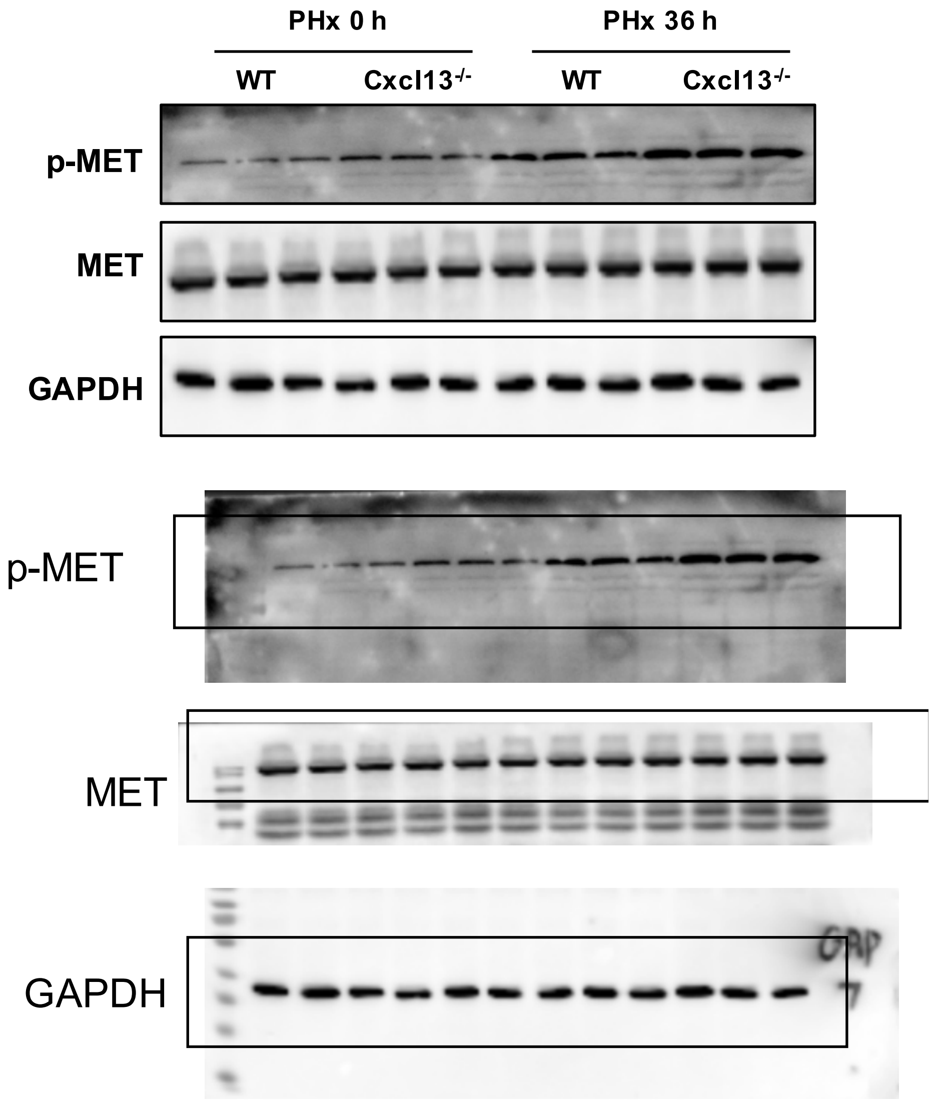
PHx 0 h
PHx 36 h
WT
Cxcl13-/-
WT
Cxcl13-/-
p-MET
MET
GAPDH
p-MET
MET
GAPDH

Fig 3n

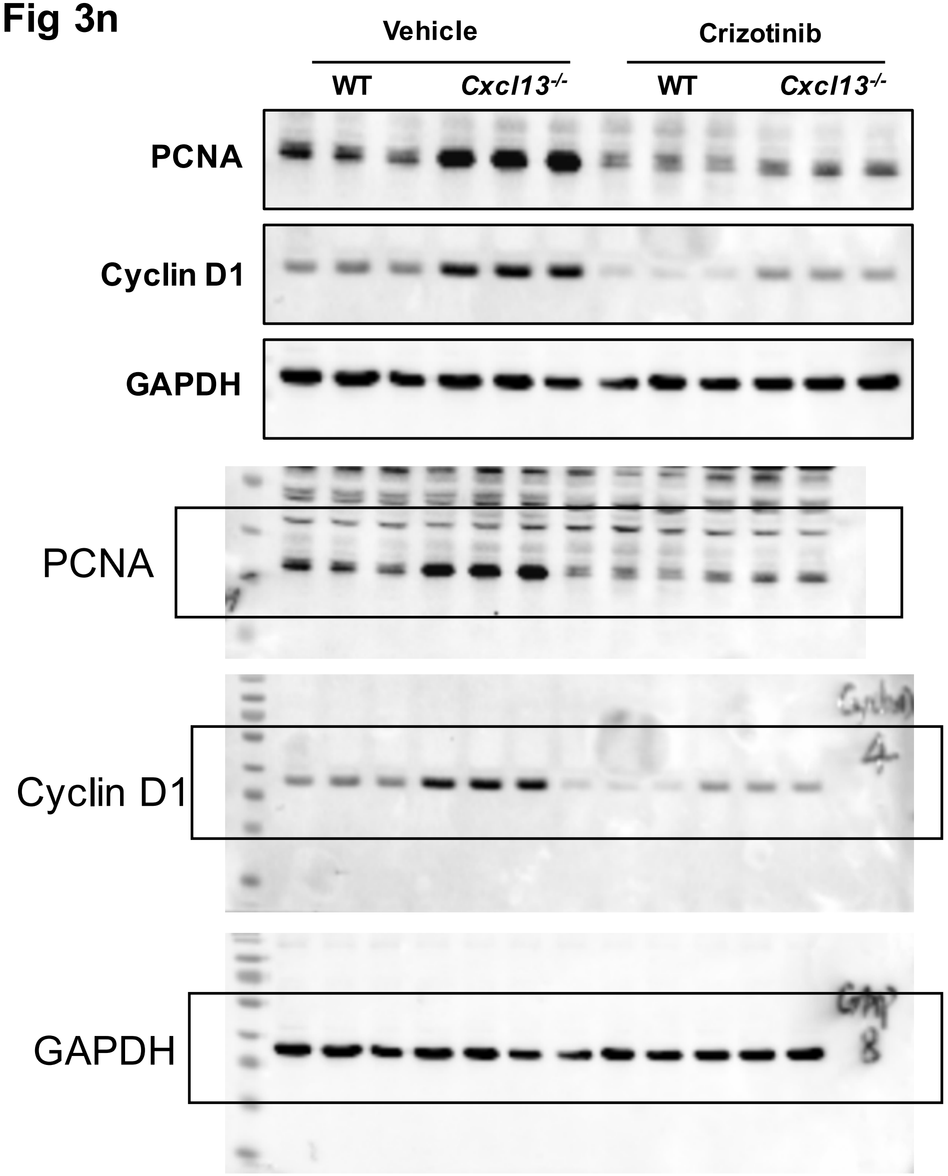
Vehicle
Crizotinib
WT
Cxcl13-/-
WT
Cxcl13-/-
PCNA
Cyclin D1
GAPDH
PCNA
Cyclin D1
GAPDH

Fig 4g

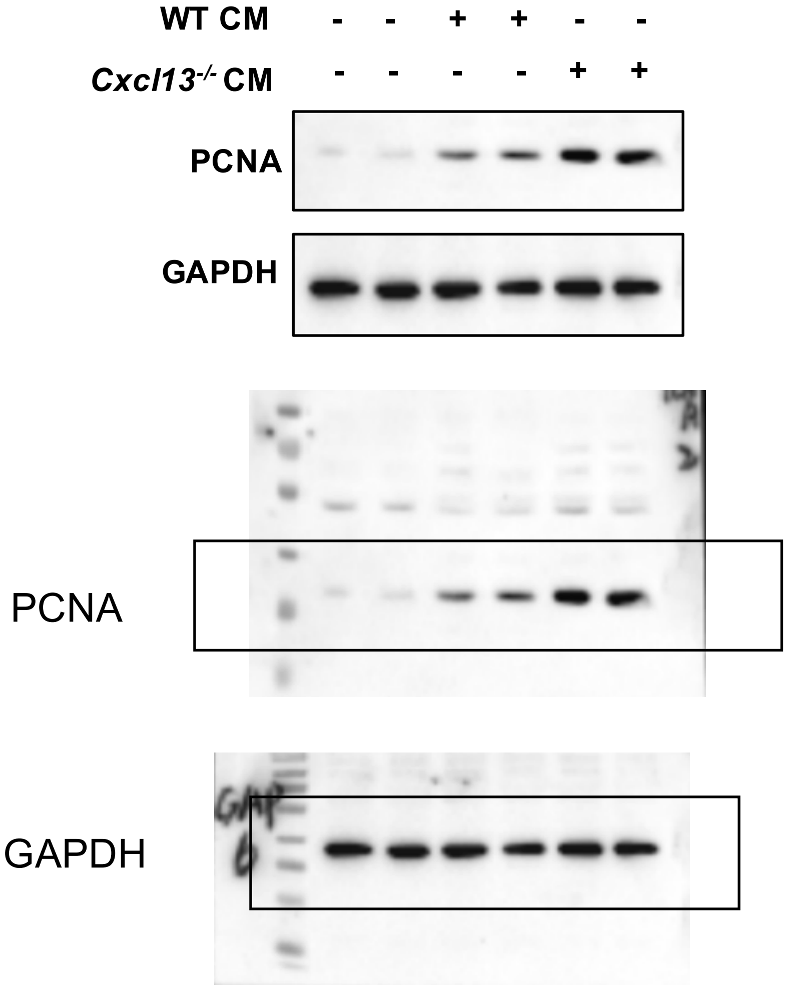
WT CM - - + + - -
Cxcl13-/- CM - - - - + +
PCNA
GAPDH
PCNA
GAPDH

Fig 4h

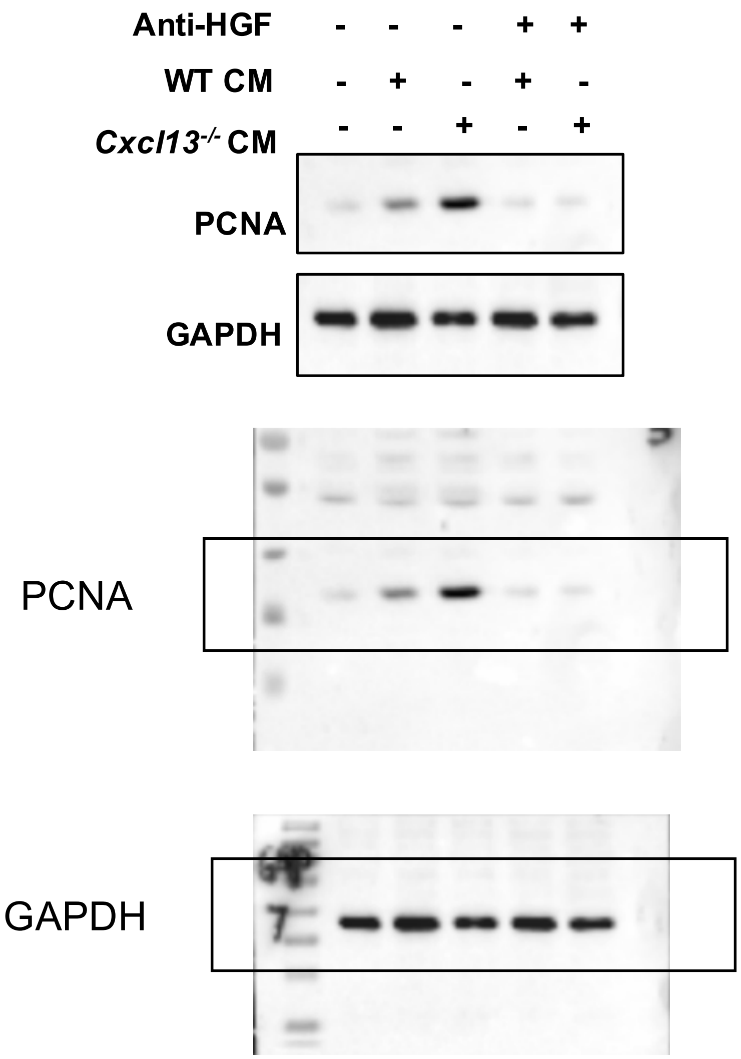
Anti-HGF - - - + +
WT CM - + - + -
Cxcl13-/- CM - - + - +
PCNA
GAPDH
PCNA
GAPDH

Fig 4i

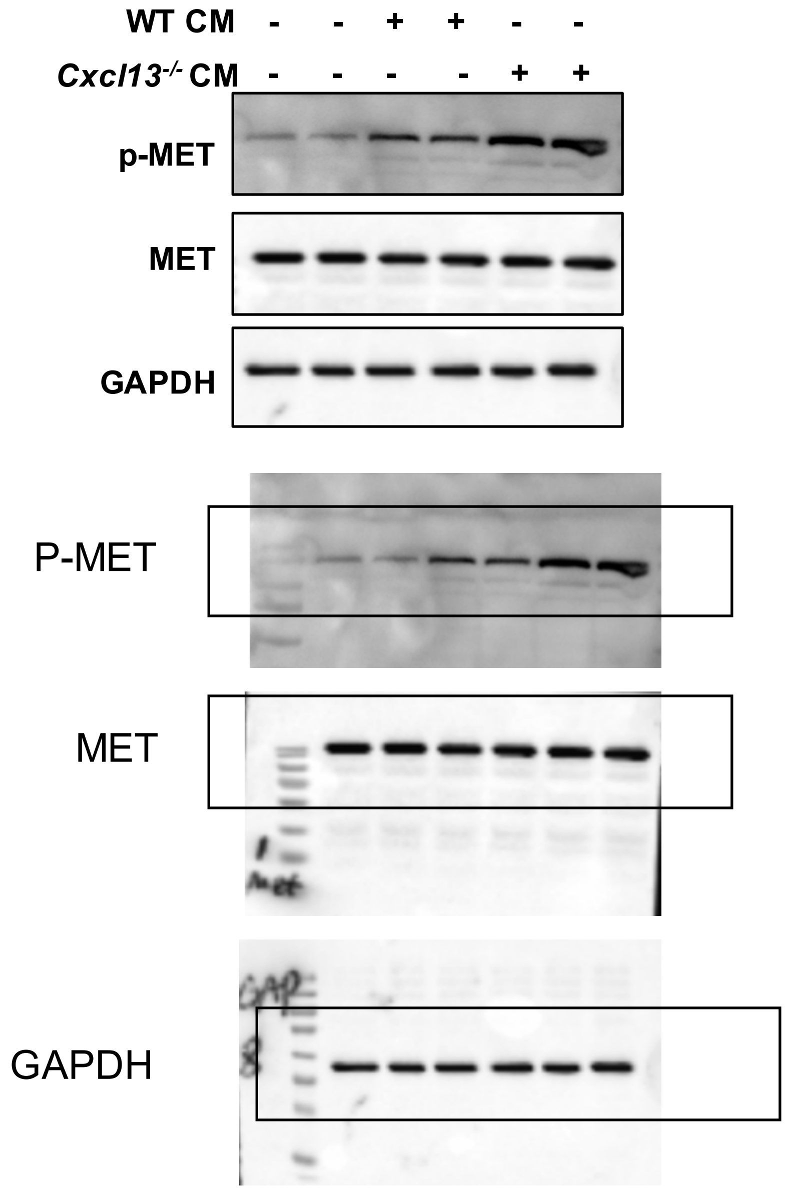
WT CM - - + + - -
Cxcl13-/- CM - - - - + +
p-MET
MET
GAPDH
P-MET
MET
GAPDH

Fig 4j

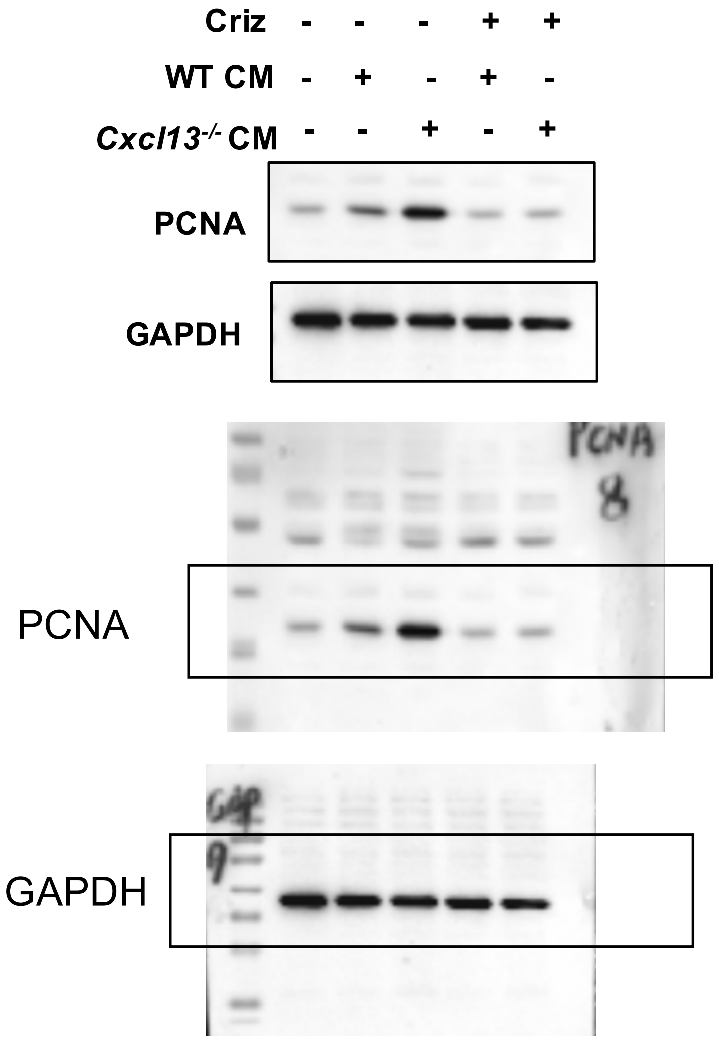
Criz - - - + +
WT CM - + - + -
Cxcl13-/- CM - - + - +
PCNA
GAPDH
PCNA
GAPDH

Fig 5e

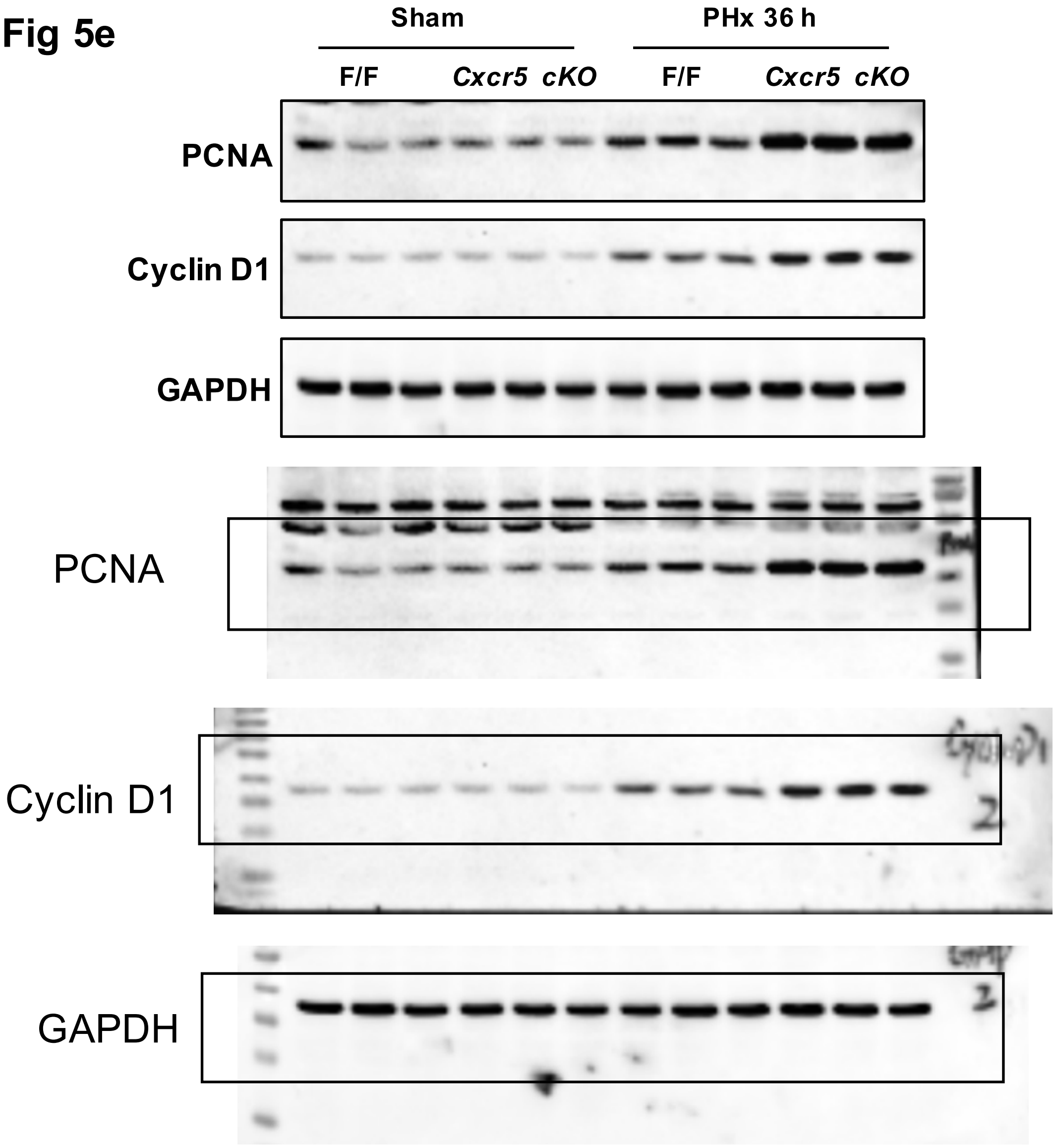
Sham
PHx 36 h
F/F
Cxcr5 cKO
F/F
Cxcr5 cKO
PCNA
Cyclin D1
GAPDH
PCNA
Cyclin D1
GAPDH

Fig 5k

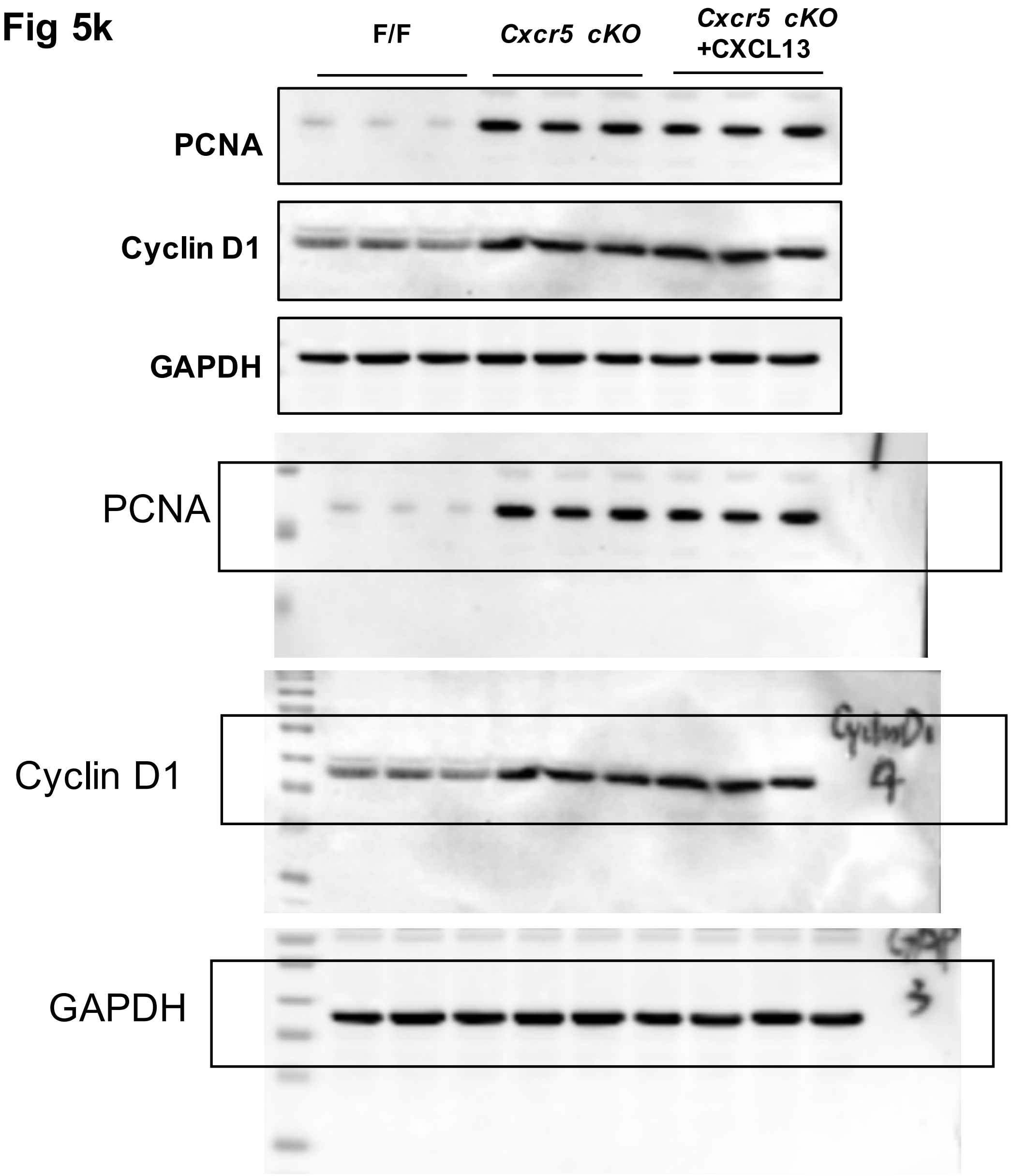
F/F
Cxcr5 cKO
Cxcr5 cKO
+CXCL13
PCNA
Cyclin D1
GAPDH
PCNA
Cyclin D1
GAPDH

Fig 5p

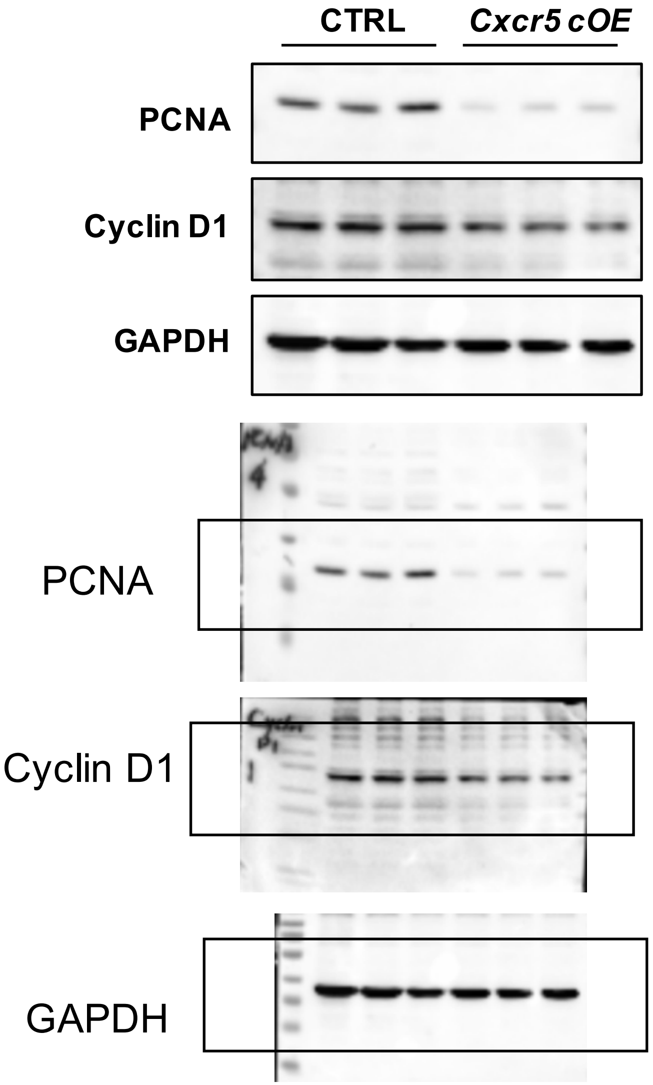
CTRL
Cxcr5 cOE
PCNA
Cyclin D1
GAPDH
PCNA
Cyclin D1
GAPDH

Fig 6b

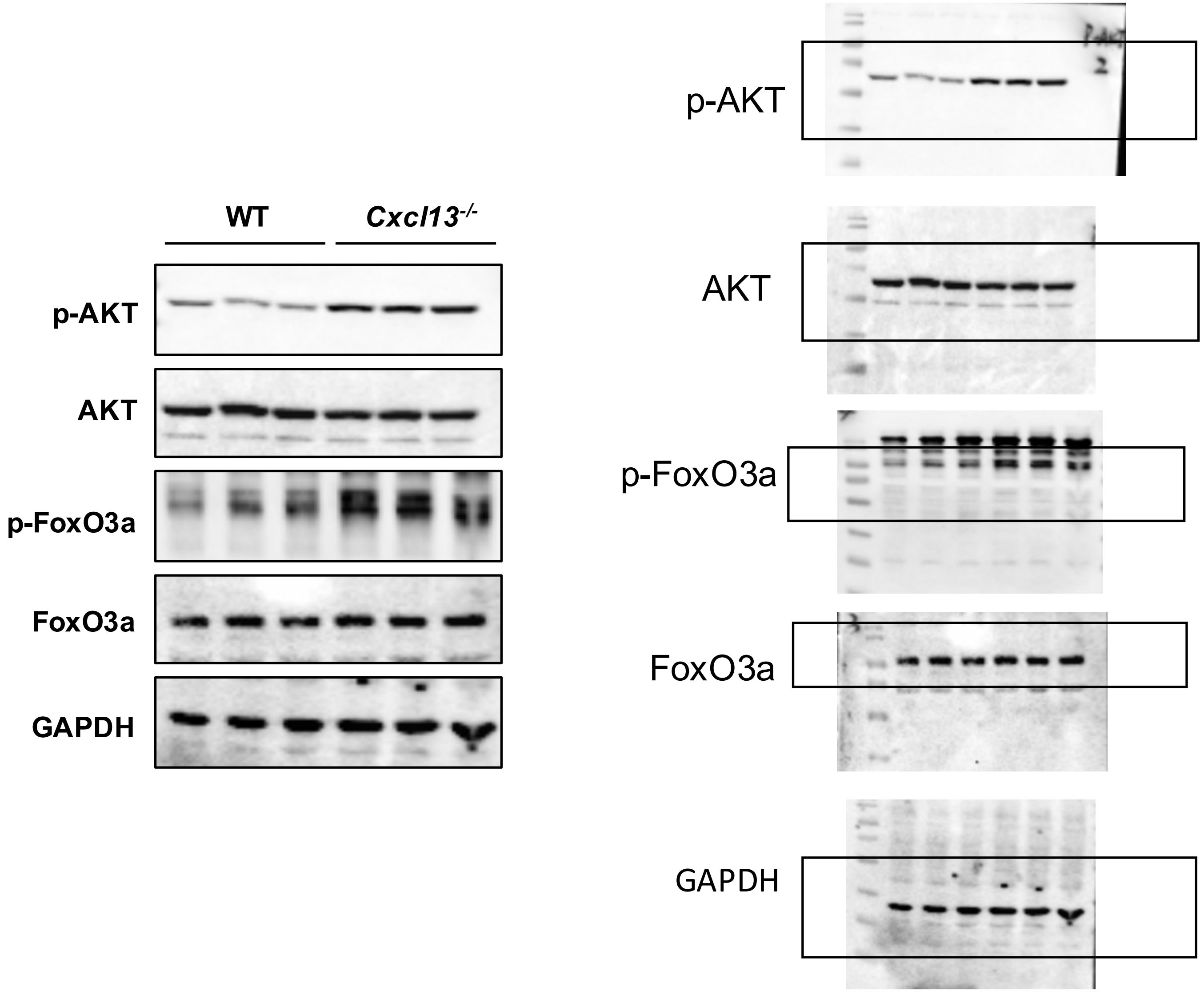
WT
Cxcl13-/-
p-AKT
AKT
p-FoxO3a
FoxO3a
GAPDH
p-AKT
AKT
p-FoxO3a
FoxO3a
GAPDH

Fig 6c

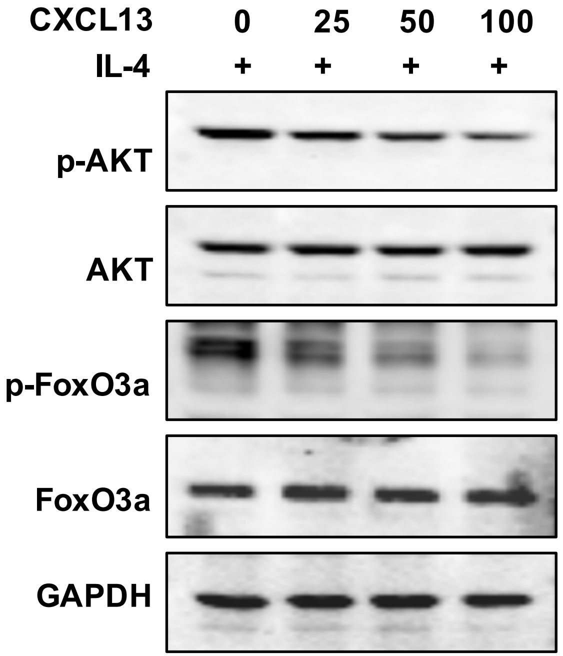
CXCL13 0 25 50 100
IL-4 + + + +
p-AKT
AKT
p-FoxO3a
FoxO3a
GAPDH

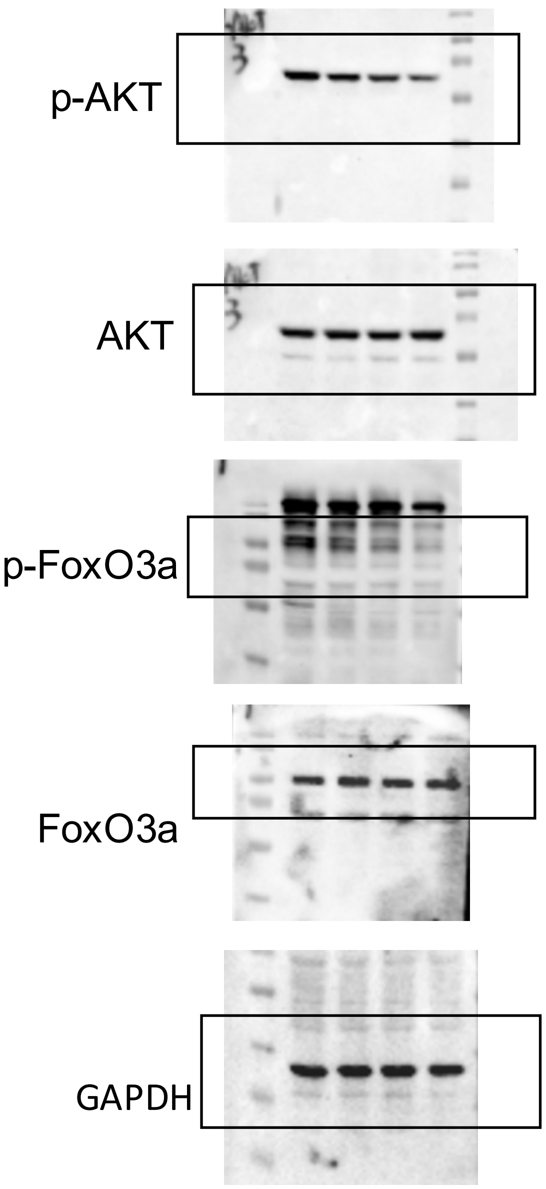
p-AKT
AKT
p-FoxO3a
FoxO3a
GAPDH

Fig 6e

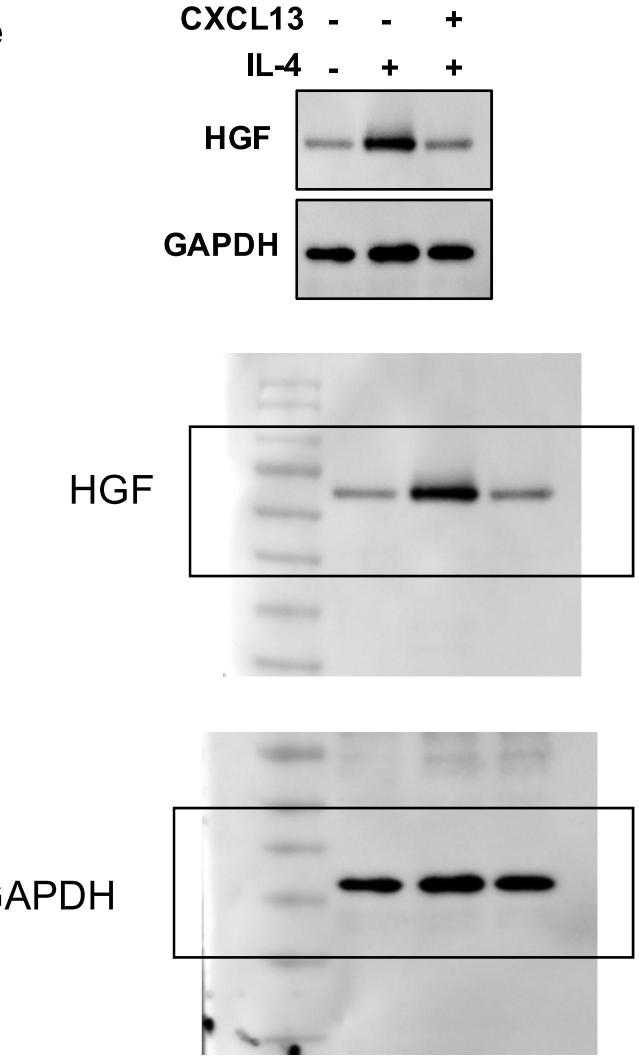
CXCL13 - - +
IL-4 - + +
HGF
GAPDH
HGF
GAPDH

Fig 6f

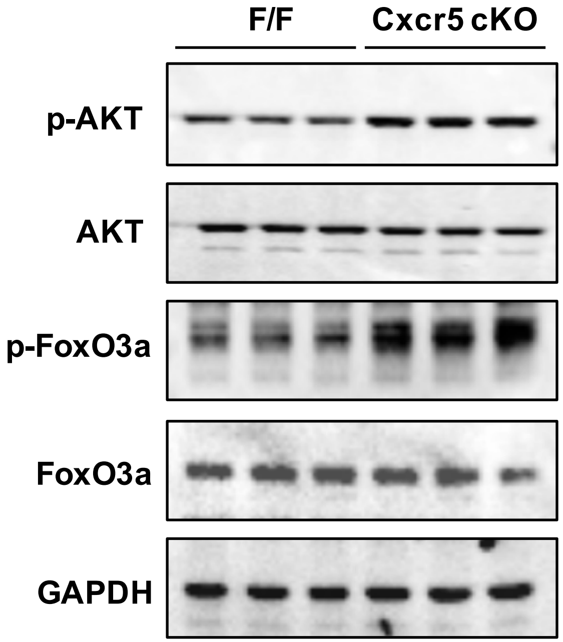
F/F
Cxcr5 cKO
p-AKT
AKT
p-FoxO3a
FoxO3a
GAPDH

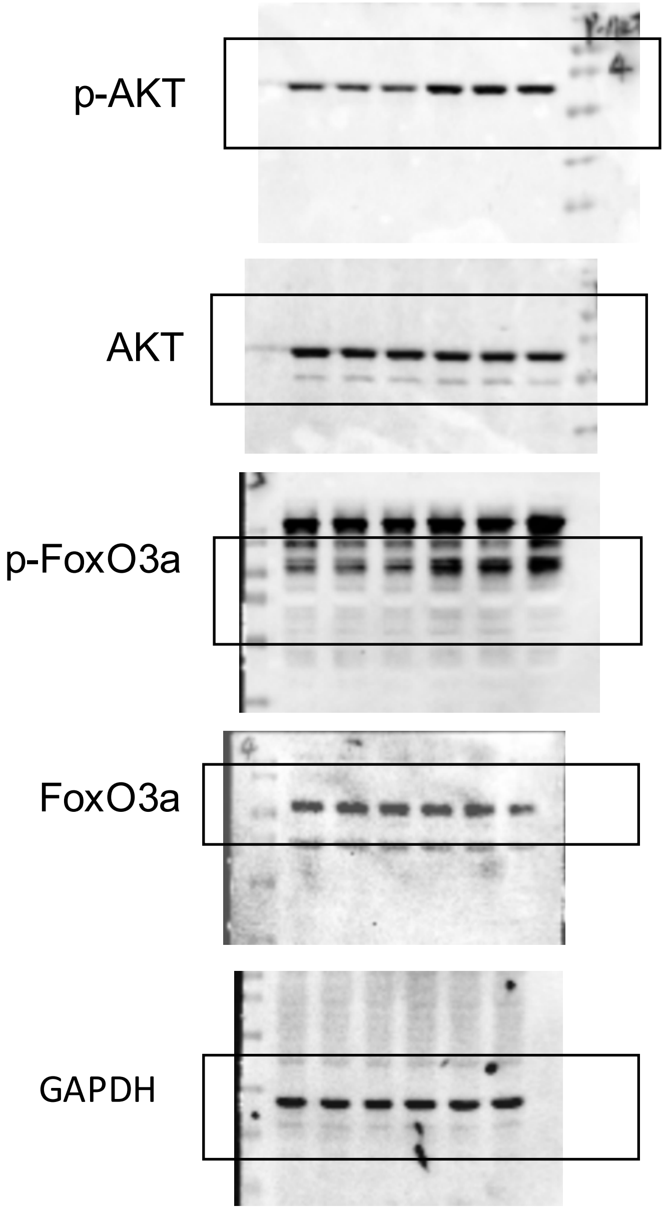
p-AKT
AKT
p-FoxO3a
FoxO3a
GAPDH

Fig 6g

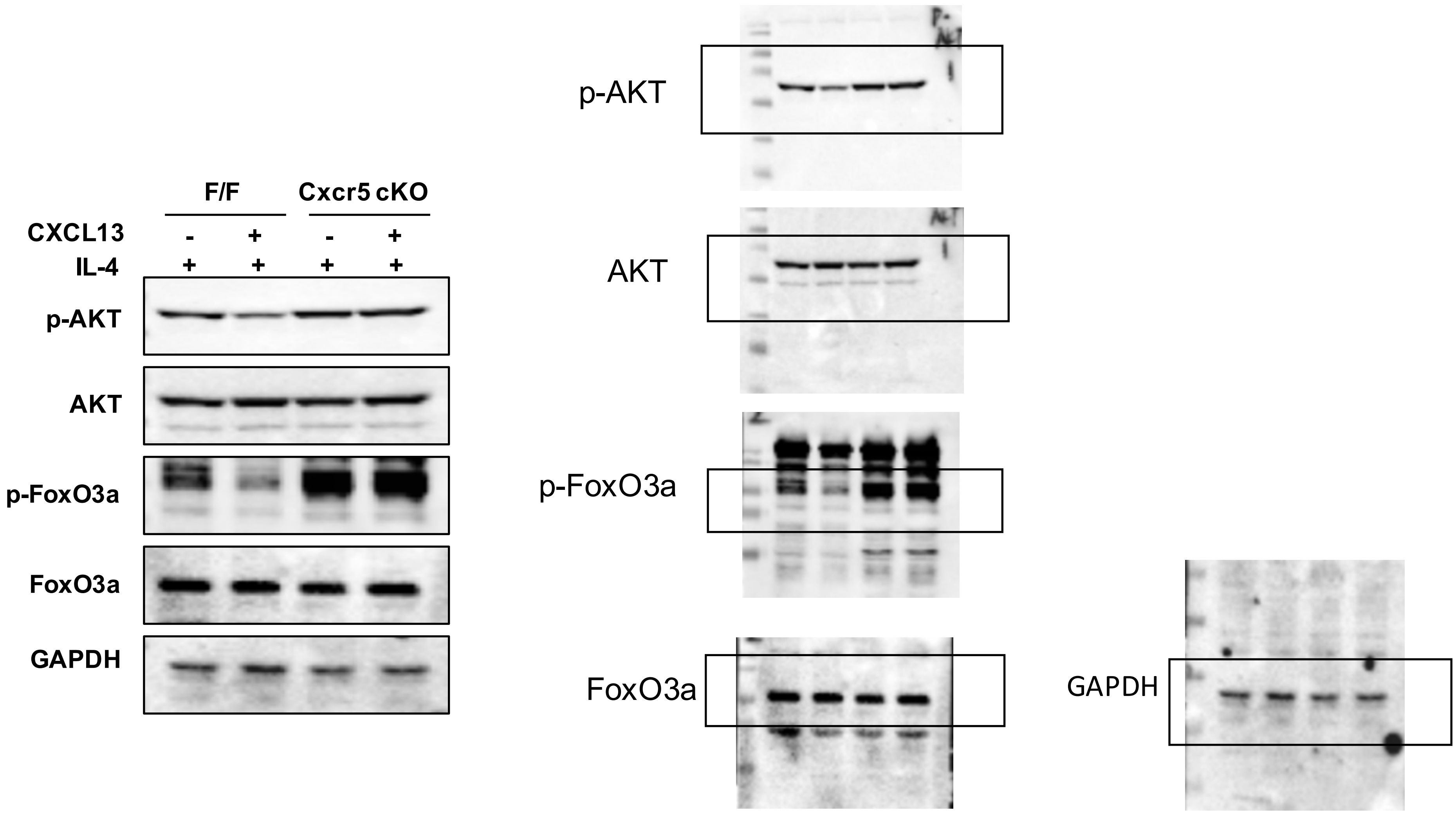

F/F
Cxcr5 cKO
CXCL13 - + - +
IL-4 + + + +
p-AKT
AKT
p-FoxO3a
FoxO3a
GAPDH
p-AKT
AKT
p-FoxO3a
FoxO3a
GAPDH

Fig 6i

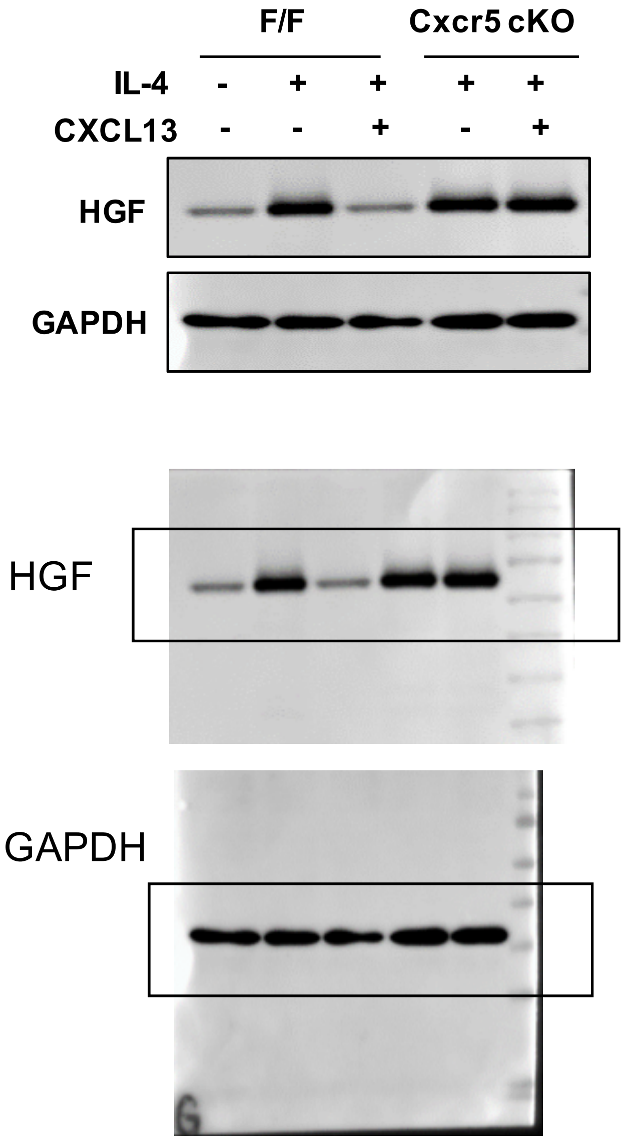

Fig 6k

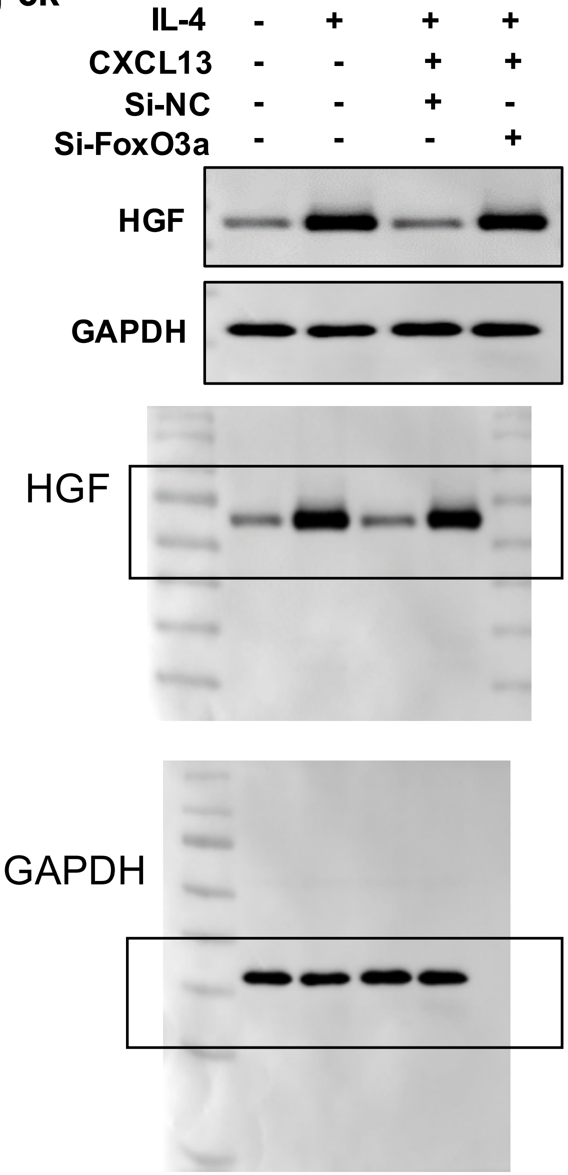

Fig 6l

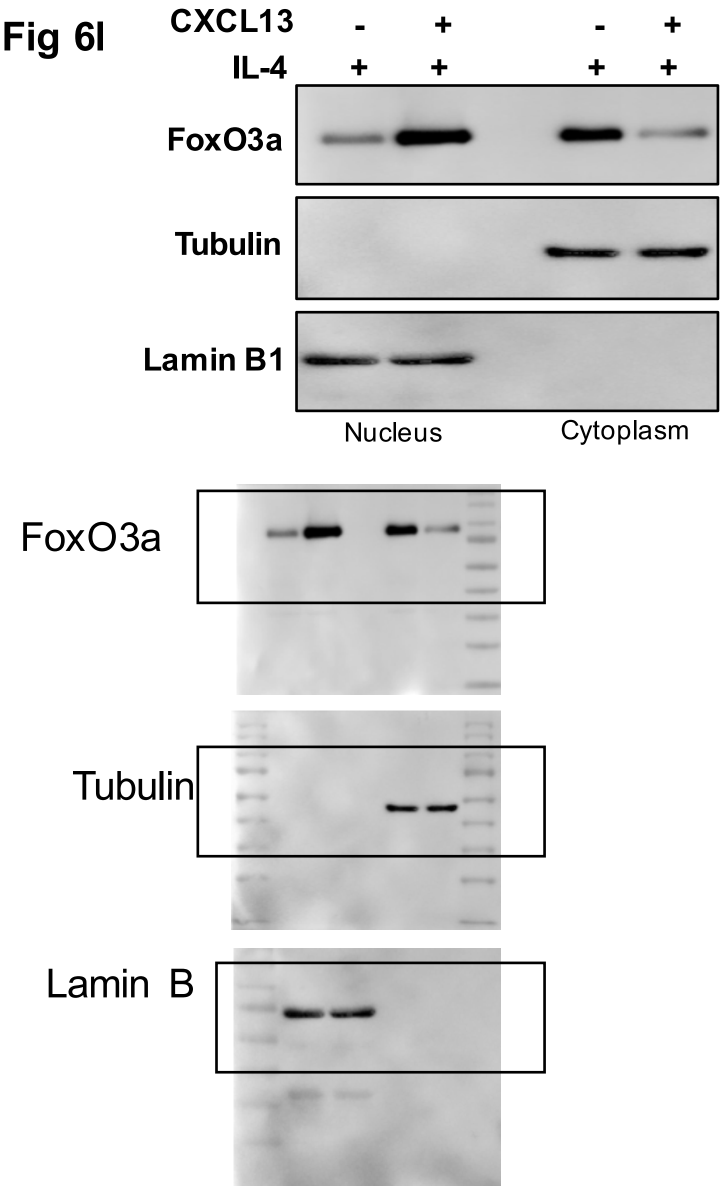

Fig 6n

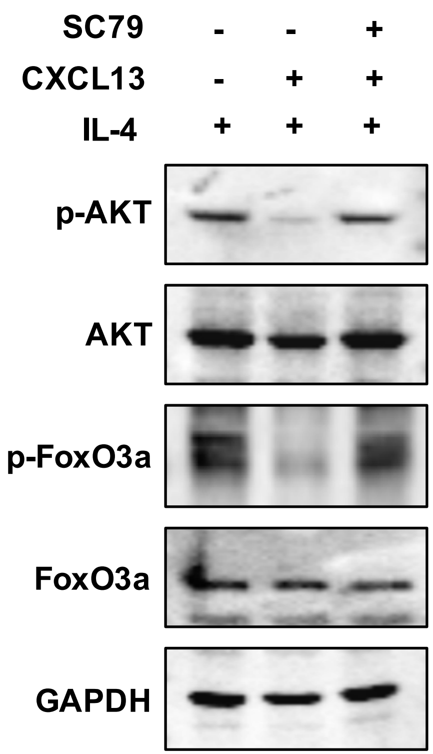
SC79 - - +
CXCL13 - + +
IL-4 + + +
p-AKT
AKT
p-FoxO3a
FoxO3a
GAPDH

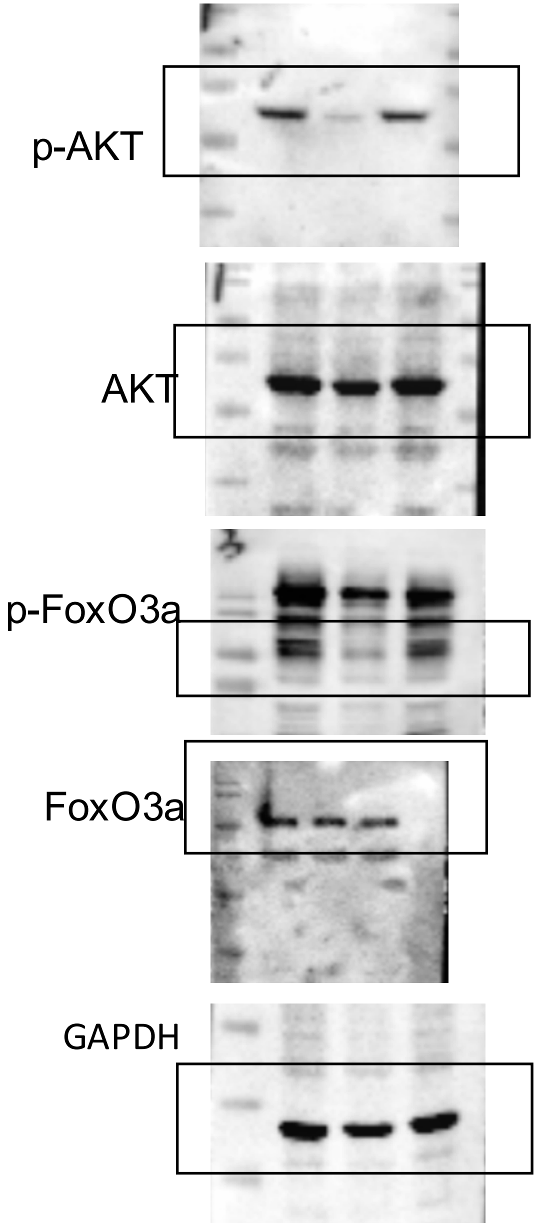
p-AKT
AKT
p-FoxO3a
FoxO3a
GAPDH

Fig 6p

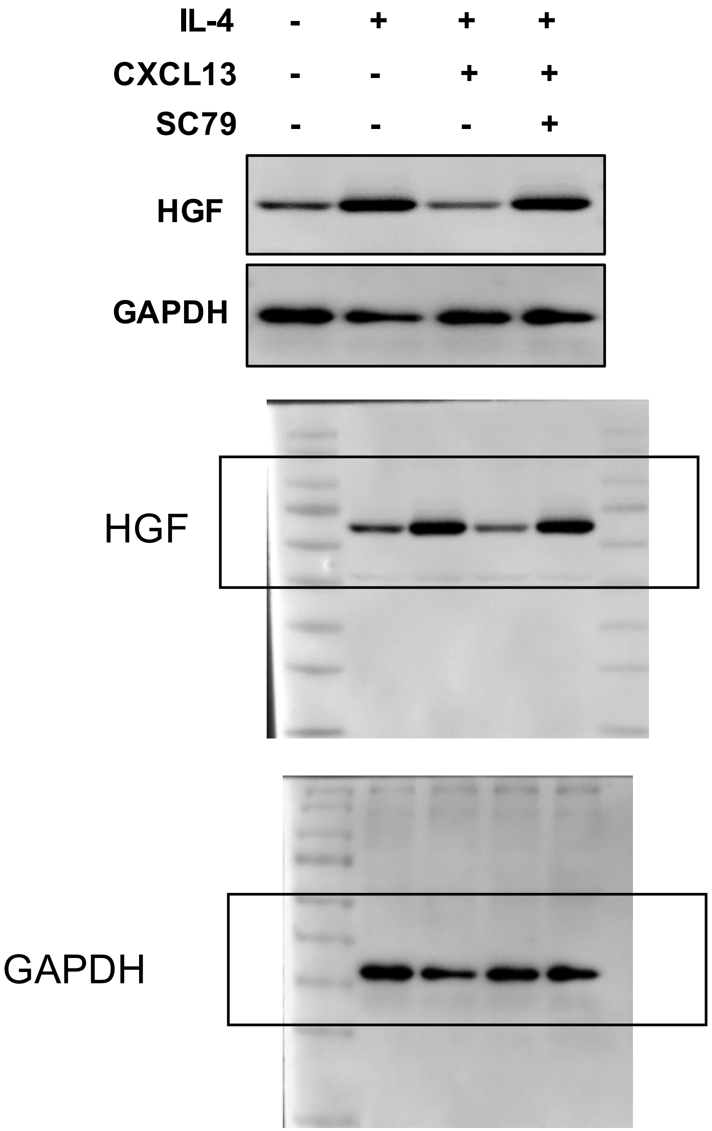
IL-4 - + + +
CXCL13 - - + +
SC79 - - - +
HGF
GAPDH
HGF
GAPDH

Fig 7f

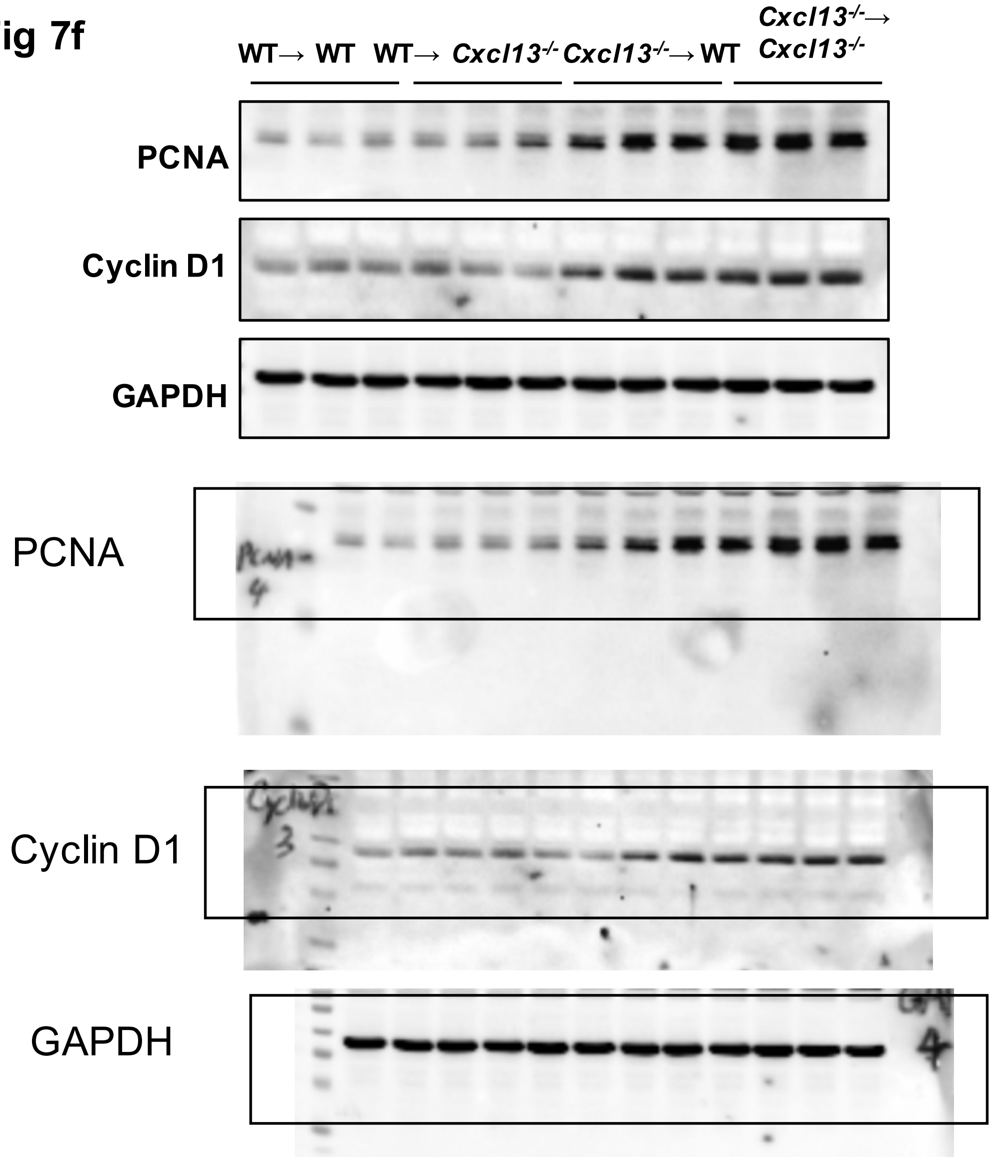

WT→ WT
WT→ *Cxcl13*-/-
Cxcl13-/-→ WT
Cxcl13-/-→ *Cxcl13*-/-
PCNA
Cyclin D1
GAPDH
PCNA
Cyclin D1
GAPDH

Fig 7i

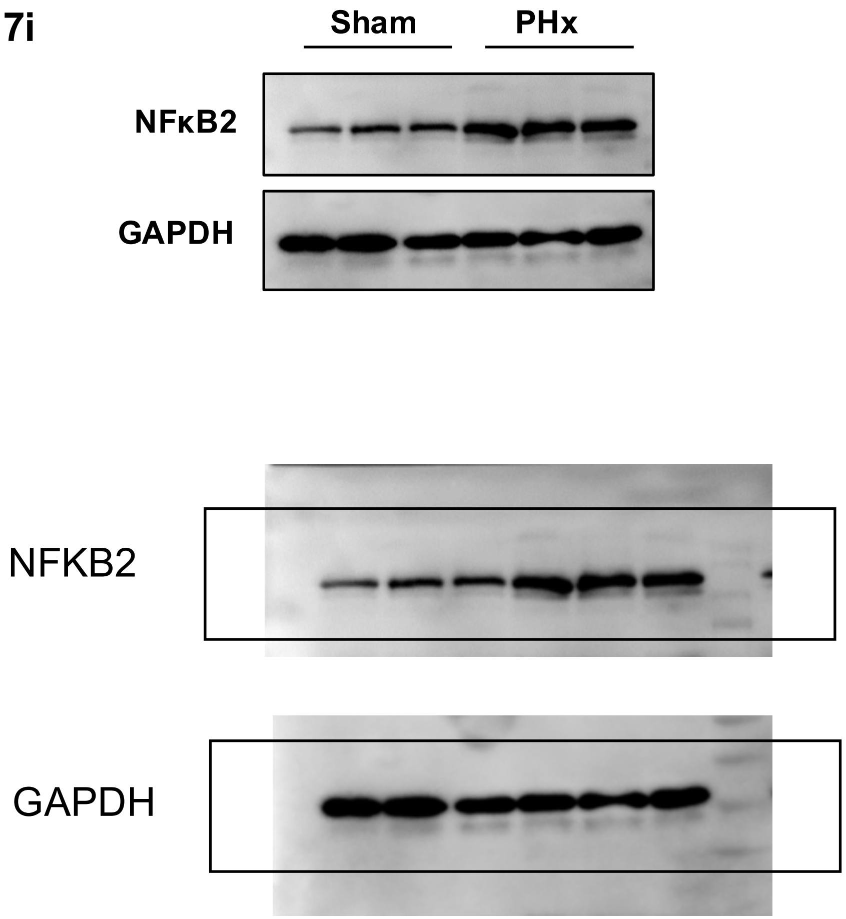

Sham
PHx
NFκB2
GAPDH
NFKB2
GAPDH

Fig 7q

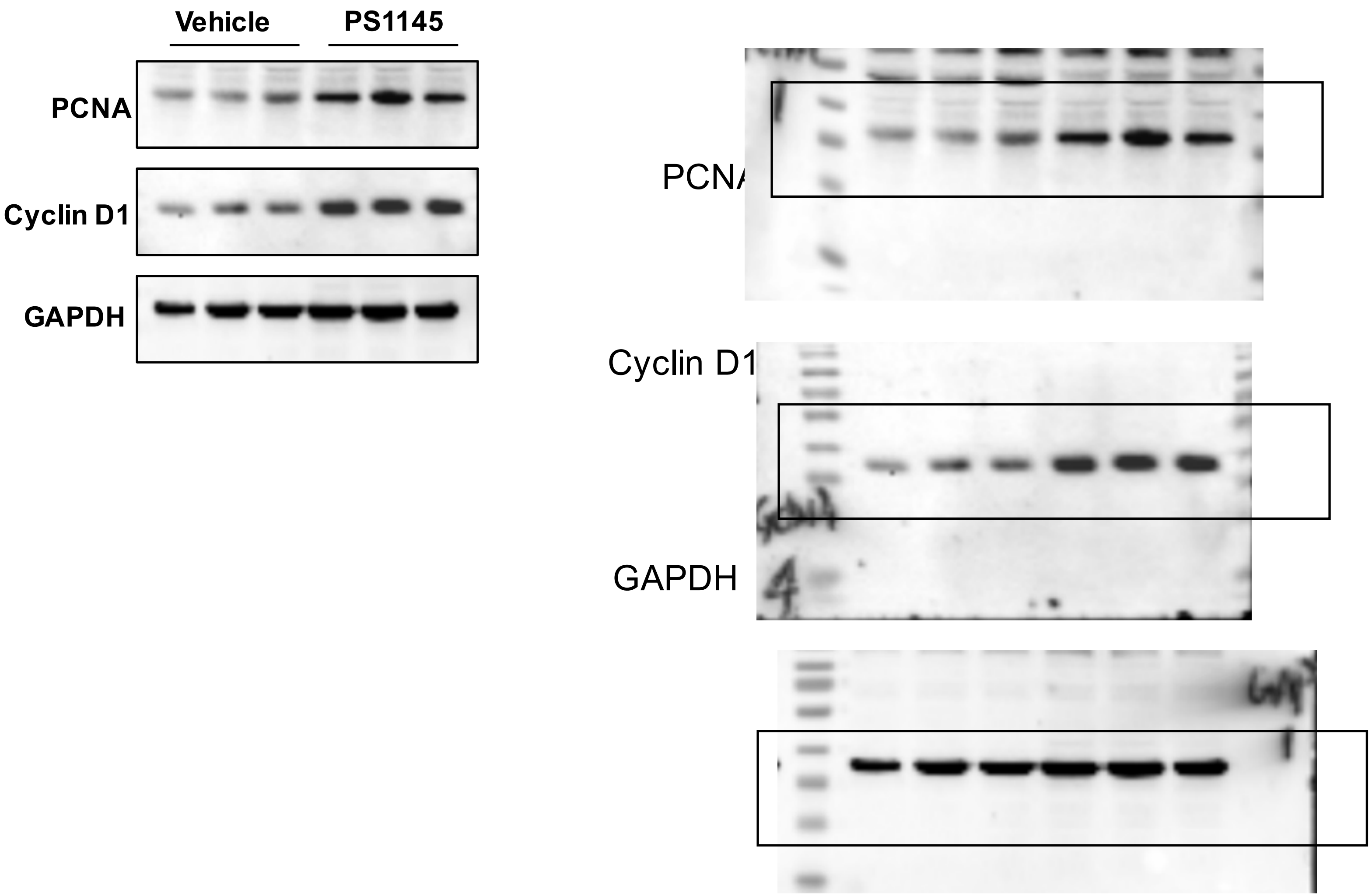

Fig 8g

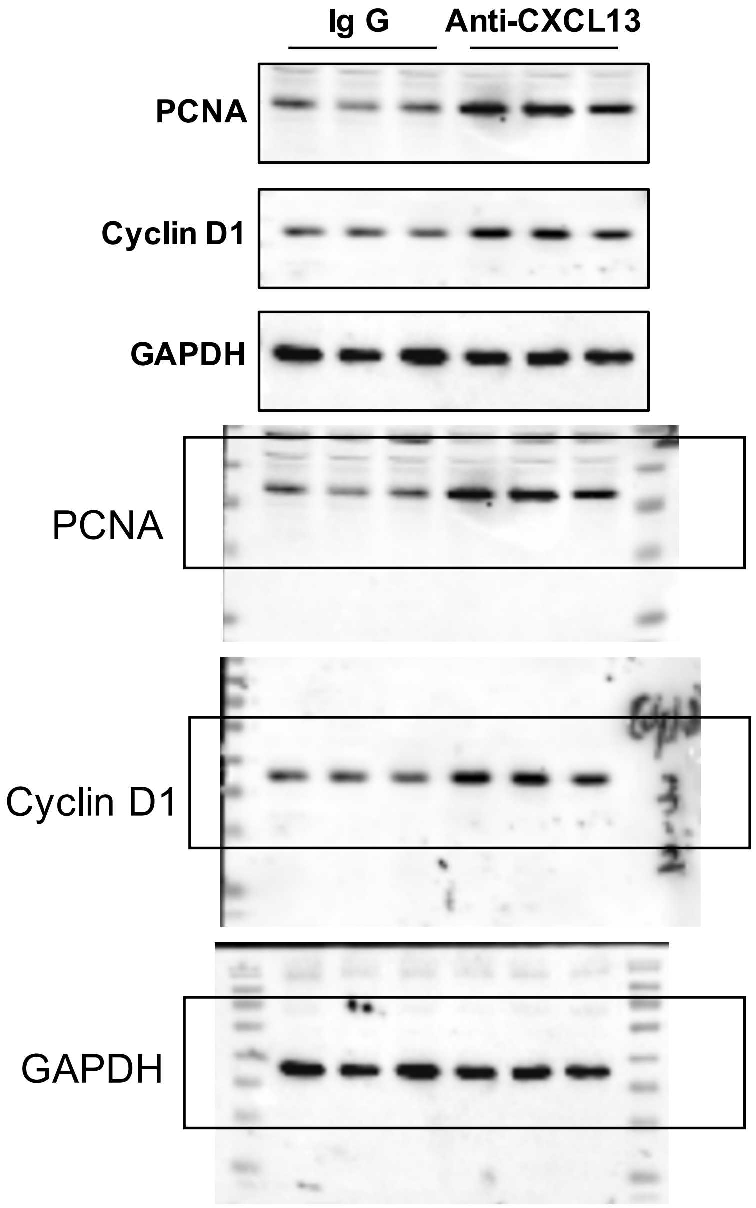
Ig G
Anti-CXCL13
PCNA
Cyclin D1
GAPDH
PCNA
Cyclin D1
GAPDH

Fig 8j

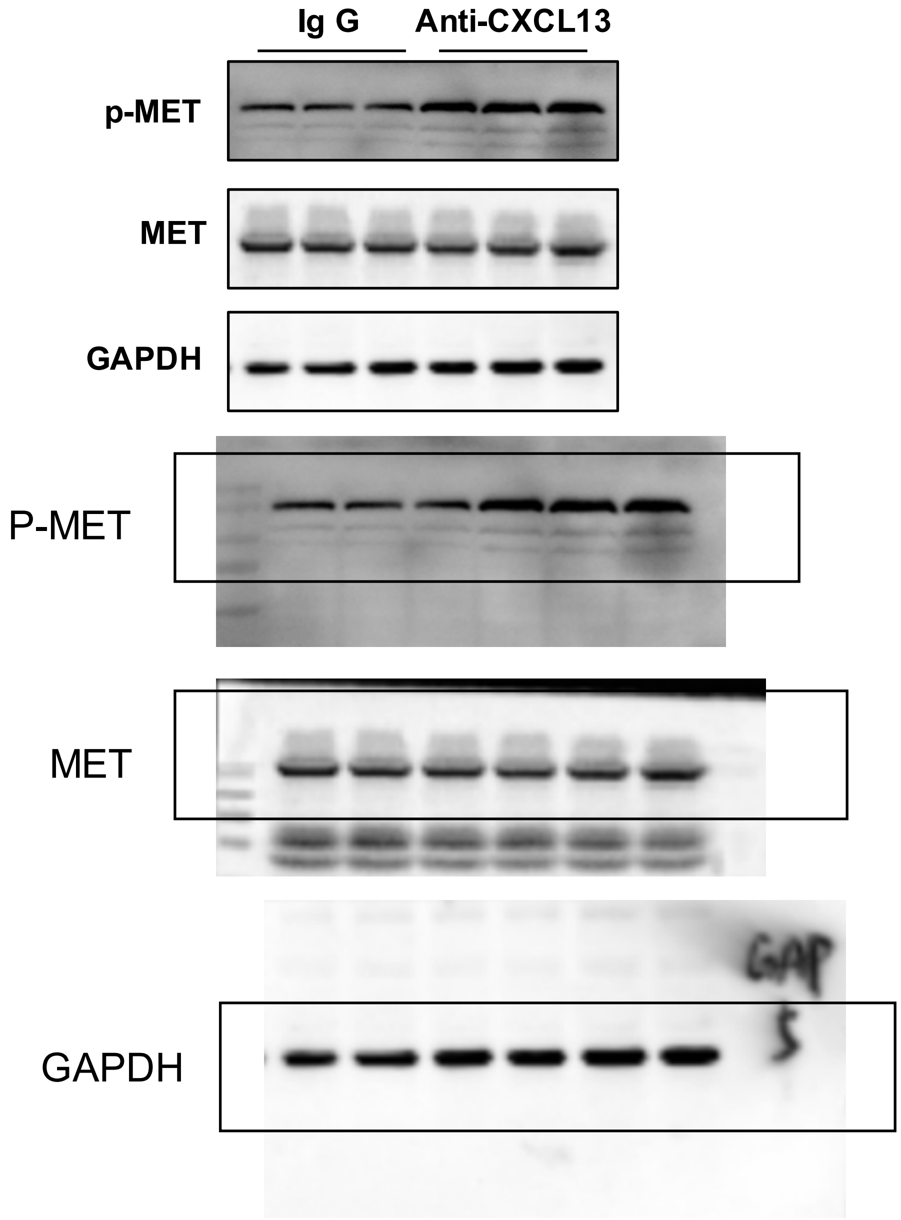
Ig G
Anti-CXCL13
p-MET
MET
GAPDH
P-MET
MET
GAPDH

Fig S2b

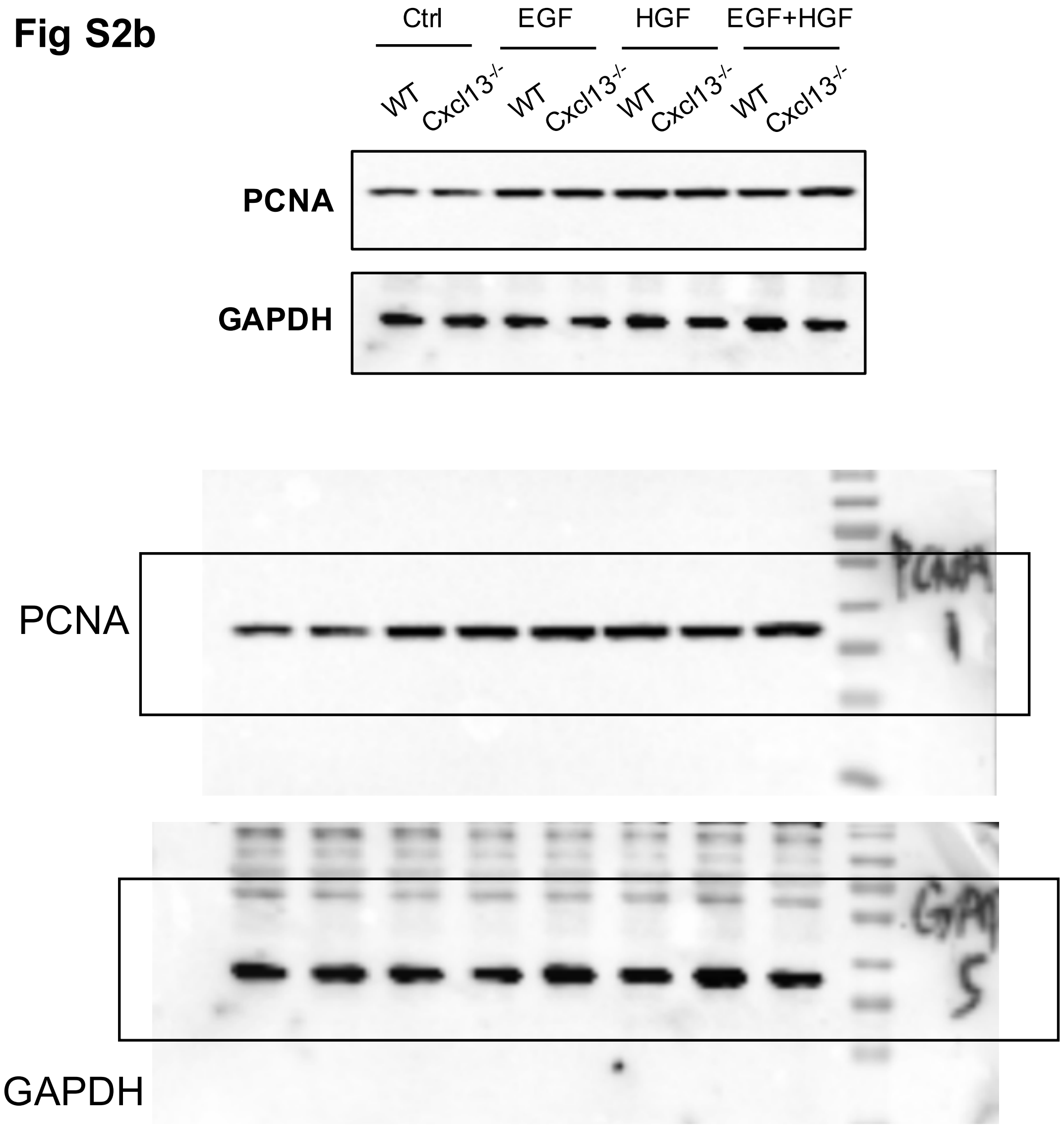

Fig S2c

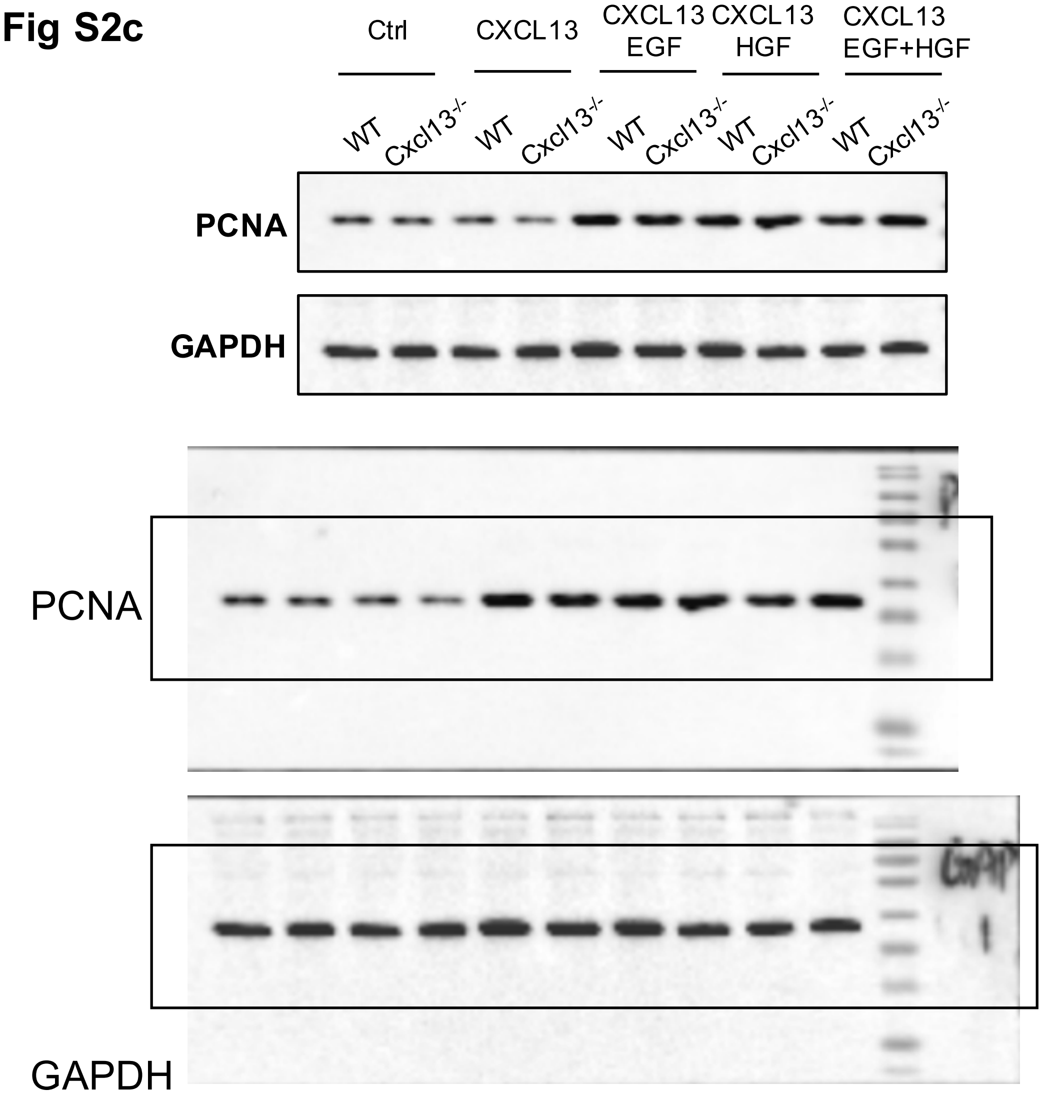

S5d

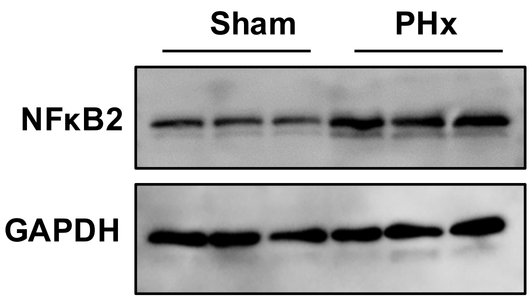

In liver tissues

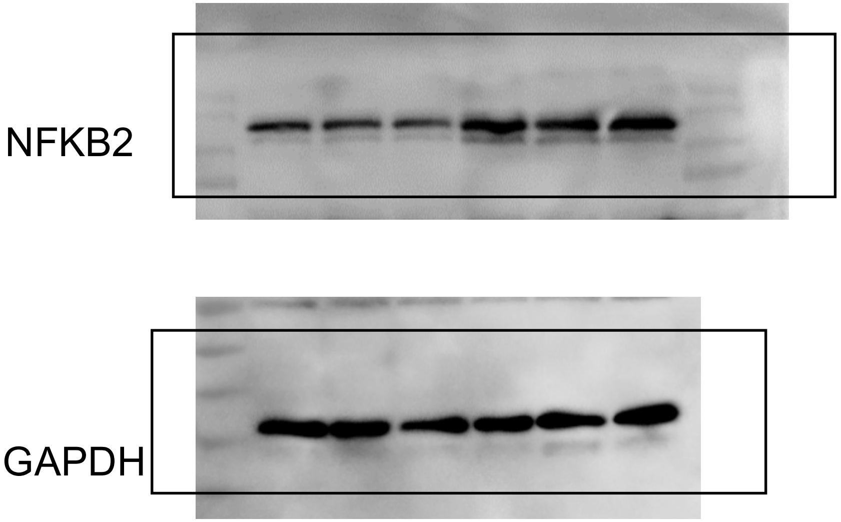

Supplement: Supplementary file 2 — Raw wb images [file 41419_2025_7568_MOESM2_ESM.pdf]
